# Involvement of lncRNA *MIR205HG* in idiopathic pulmonary fibrosis and IL-33 regulation via *Alu* elements

**DOI:** 10.1172/jci.insight.187172

**Published:** 2025-03-10

**Authors:** Tsuyoshi Takashima, Chao Zeng, Eitaro Murakami, Naoko Fujiwara, Masaharu Kohara, Hideki Nagata, Zhaozu Feng, Ayako Sugai, Yasue Harada, Rika Ichijo, Daisuke Okuzaki, Satoshi Nojima, Takahiro Matsui, Yasushi Shintani, Gota Kawai, Michiaki Hamada, Tetsuro Hirose, Kazuhiko Nakatani, Eiichi Morii

**Affiliations:** 1 Department of Pathology, Osaka University Graduate School of Medicine, Osaka, Japan.; 2 Faculty of Science and Engineering, Waseda University, Tokyo, Japan.; 3 Department of Regulatory Bioorganic Chemistry, SANKEN (the Institute of Scientific and Industrial Research), Osaka, Japan.; 4 Graduate School of Frontier Biosciences, Osaka University, Osaka, Japan.; 5 Department of General Thoracic Surgery, Osaka University Graduate School of Medicine, Osaka, Japan.; 6 Department of Life Science, Graduate School of Advanced Engineering, Chiba Institute of Technology, Chiba, Japan.; 7 Laboratory of Human Immunology (Single Cell Genomics), WPI Immunology Frontier Research Center, and; 8 Institute for Open and Transdisciplinary Research Initiatives, Osaka University, Osaka, Japan.; 9 AIST-Waseda University Computational Bio Big-Data Open Innovation Laboratory (CBBD-OIL), National Institute of Advanced Industrial Science and Technology, Tokyo, Japan.

**Keywords:** Inflammation, Pulmonology, Innate immunity, Noncoding RNAs, RNA processing

## Abstract

Idiopathic pulmonary fibrosis (IPF) causes remodeling of the distal lung. Pulmonary remodeling is histologically characterized by fibrosis, as well as appearance of basal cells; however, the involvement of basal cells in IPF remains unclear. Here, we focus on the long noncoding RNA *MIR205HG*, which is highly expressed in basal cells, using RNA sequencing. Through RNA sequencing of genetic manipulations using primary cells and organoids, we discovered that *MIR205HG* regulates IL-33 expression. Mechanistically, the *AluJb* element of *MIR205HG* plays a key role in IL-33 expression. Additionally, we identified a small molecule that targets the *AluJb* element, leading to decreased IL-33 expression. IL-33 is known to induce type 2 innate lymphoid cells (ILC2s), and we observed that *MIR205HG* expression was positively correlated with the number of ILC2s in patients with IPF. Collectively, these findings provide insights into the mechanisms by which basal cells contribute to IPF and suggest potential therapeutic targets.

## Abstract 2

LncRNA MIR205HG was identified as a prognostic factor of idiopathic pulmonary fibrosis and stabilized IL33 via the interaction of their Alu elements in pulmonary epithelium.

## Graphical abstract

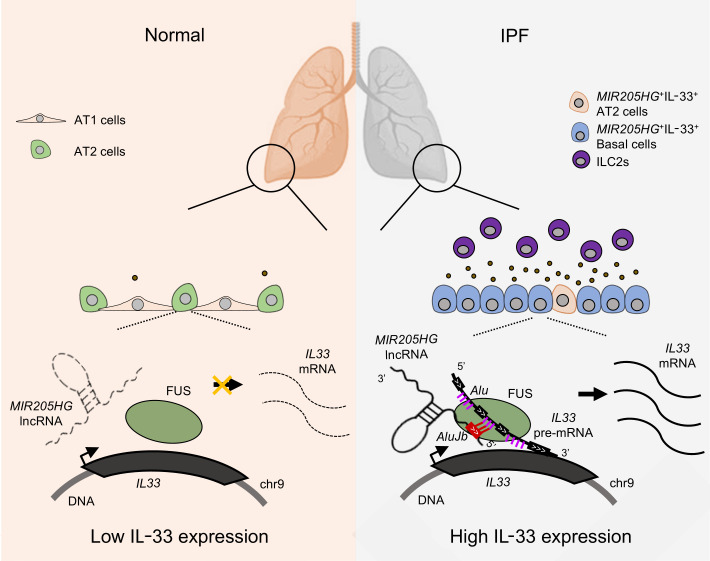

## Introduction

Idiopathic pulmonary fibrosis (IPF) is a progressive lung disease that causes remodeling and fibrosis of the distal lung, ultimately leading to respiratory failure and death (1, 2). The pathogenesis of IPF has not been fully elucidated, but repeated inflammation is thought to cause epithelial cell damage and dysfunction, which lead to fibroblast activation, deposition of extracellular matrix, and subsequent tissue fibrosis (3). Nintedanib and pirfenidone, currently the only US Food and Drug Administration–approved drugs, do not completely halt disease progression (4, 5). Therefore, it is crucial to understand the pathogenesis of IPF and uncover molecular mechanisms to improve the prognosis of IPF.

The distal region of normal lungs is histologically composed of 2 types of epithelial cells: alveolar type I (AT1) cells, which are responsible for gas exchange, and alveolar type II (AT2) cells, which produce surfactant and act as tissue stem cells. However, the distal region of the IPF exhibits histologically characteristic findings termed “honeycombed” cysts, where AT1 and AT2 cells are reduced and replaced by aberrant AT2-like cells and airway epithelial cells, such as basal cells, goblet cells, and ciliated cells (2, 6). This pulmonary remodeling has also been robustly demonstrated in comprehensive single-cell RNA-sequencing (scRNA-Seq) analyses (7–9). Additionally, transcriptomic analysis of bronchoalveolar lavage fluid from patients with IPF has shown that a basal cell signature is associated with enhanced disease progression and mortality (10). However, until recently, it remained uncertain whether this pulmonary remodeling directly contributed to the pathogenesis of IPF or was simply a bystander phenomenon or end-stage finding. Recent studies have revealed that IPF-derived basal cells induced fibroblast proliferation and extracellular matrix deposition both in vitro and in vivo (11, 12). These reports suggest that basal cells directly contribute to the fibrosis of IPF, but insight into the contributions of basal cells to the pathogenesis of IPF remains limited.

lncRNAs, defined as noncoding RNA transcripts longer than 200 nucleotides, are involved in various regulatory steps of gene expression, such as chromatin remodeling, transcription, RNA stability, and translation (13–15). These transcripts are shown to be involved in the biological processes of diseases, including pulmonary fibrosis (15, 16). For example, the lncRNA *DNM3OS* has been identified as a critical downstream effector of TGF-β–induced myofibroblast activation, regulating the lung fibrotic process by producing pro-fibrotic mature miRNAs (17). Furthermore, the lncRNA *FENDRR* can reduce lung fibroblast activation by decreasing cellular iron concentration via interactions with IRP1 and acting as a pro-fibrotic *miR-214* (18). However, most of these findings have focused on fibroblasts and myofibroblasts; little is known regarding the function of lncRNA expressed in basal cells involved in the pathogenesis of IPF. Therefore, we focused on lncRNA to gain insights concerning the involvement of basal cells in the pathogenesis of IPF.

In this work, we first analyzed public scRNA-Seq data from patients with IPF and identified lncRNA *MIR205HG*, which is highly expressed in basal cells. *MIR205HG* was revealed as a prognostic factor in patients with IPF. Through comprehensive analysis of genetic manipulations using primary cells, alveolar epithelial organoids, and airway organoids from IPF patient samples (IPF patient–derived airway organoids), we found that *MIR205HG* is involved in the regulation of IL-33 expression, which is thought to contribute to the pathogenesis of IPF. Intriguingly, the interaction between the *AluJb* element of *MIR205HG* and the *Alu* element of *IL33* was important for these regulatory mechanisms. Additionally, we identified DQzG, a small molecule that reduced IL-33 expression, likely by inhibiting the interaction between *Alu* elements. Furthermore, *MIR205HG* expression was positively correlated with IL-33 expression and the number of type 2 innate lymphoid cells (ILC2s) in tissue samples from patients with IPF. These data highlight the involvement of lncRNA *MIR205HG* in the pathogenesis of IPF and provide important insights into a therapeutic target.

## Results

### lncRNA MIR205HG is highly expressed in basal cells and an independent poor prognostic factor in IPF.

**Figure 1:**
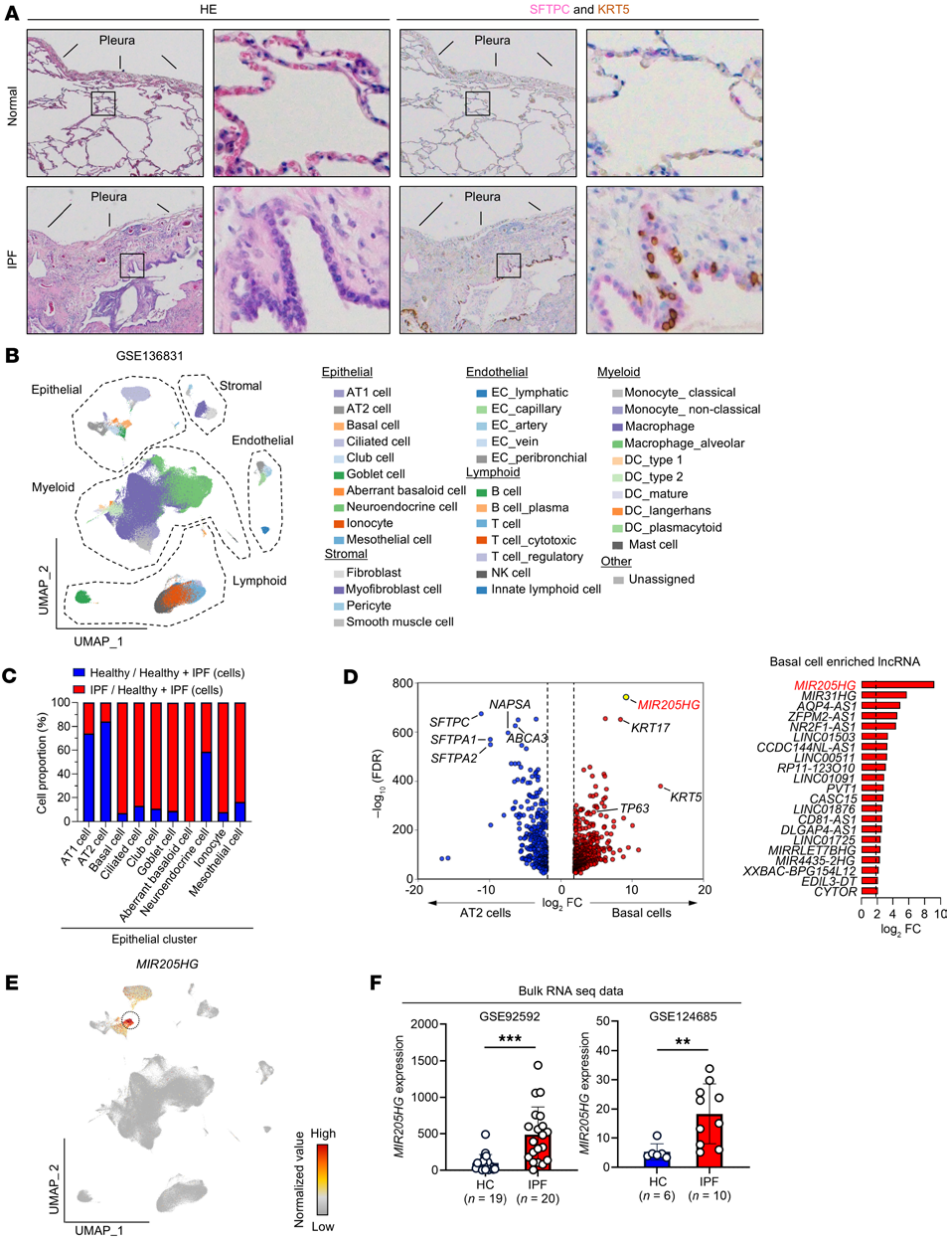
lncRNA *MIR205HG* is upregulated in basal cells. (**A**) Representative images of HE, SFTPC IHC, and KRT5 IHC staining in alveoli beneath the pleura of healthy and IPF lungs. Scale bar: 100 μm. HE, hematoxylin and eosin; SFTPC, surfactant protein C; KRT5, keratin 5. (**B**) Uniform manifold approximation and projection (UMAP) visualization of cell types in healthy and IPF lungs. (**C**) Proportion of epithelial cell type distribution in healthy and IPF lungs. (**D**) Volcano plot and bar graph (basal cell enriched lncRNA) of DEGs in AT2 cells and basal cells. The cutoff values were log_2_FC > 1, FDR < 0.05. (**E**) UMAP visualization of *MIR205HG* expression. (**F**) Expression of *MIR205HG* in healthy control and IPF patients. Public bulk RNA-Seq datasets (GSE92592, ref. 19, and GSE124685, ref. 20) were used. Data represent mean ± SD. **P < 0.01, ***P < 0.001; *P* values were determined by 2-tailed Mann-Whitney *U* test. (**B**–**E**) Public scRNA-Seq data (GSE136831) (7) were used for analysis.

We examined the differences in histological features in alveolar regions between healthy lungs and IPF patient samples. In healthy lungs, alveoli beneath the pleura were lined with AT1 and AT2 cells, whereas in patients with IPF, these alveoli were lined with basophilic bronchial cells due to metaplasia (Figure 1A). The lining cells of normal lungs were positive for SFTPC, whereas those of patients with IPF were positive for KRT5 (Figure 1A). We analyzed public scRNA-Seq data from National Center for Biotechnology Information (NCBI) Gene Expression Omnibus (GEO) (GSE136831) (7), which included samples from healthy lungs (*n* = 28) and patients with IPF (*n* = 32) (Figure 1B and Supplemental Figure 1A; supplemental material available online with this article; https://doi.org/10.1172/jci.insight.187172DS1), and found that the number of alveolar epithelial cells (including AT2 cells) decreased whereas the number of airway epithelial cells (including basal cells) increased in IPF (Figure 1C). These findings, including the histological features, were consistent with the previous report (7).

Next, we searched for lncRNAs preferentially expressed in basal cells by comparing AT2 cells and basal cells in public scRNA-Seq data, identifying *MIR205HG* as the most significant differentially expressed gene (DEG) in basal cells (Figure 1D). Among the lncRNAs expressed in basal cells, *MIR205HG* was localized to the epithelial cluster and was particularly highly expressed in basal cells (Figure 1E and Supplemental Figure 1B). We sorted AT2 cells and basal cells from metaplastic lesions of 2 IPF cases and performed RNA-Seq, which verified that *MIR205HG* was among the DEGs preferentially expressed in basal cells (Supplemental Figure 2, A–G). Bulk RNA-Seq data also showed significantly higher expression of *MIR205HG* in patients with IPF than in healthy controls (GSE92592, ref. 19, and GSE124685, ref. 20) (Figure 1F). These findings indicate that *MIR205HG* is highly expressed in basal cells within metaplastic lesions.

**Figure 2:**
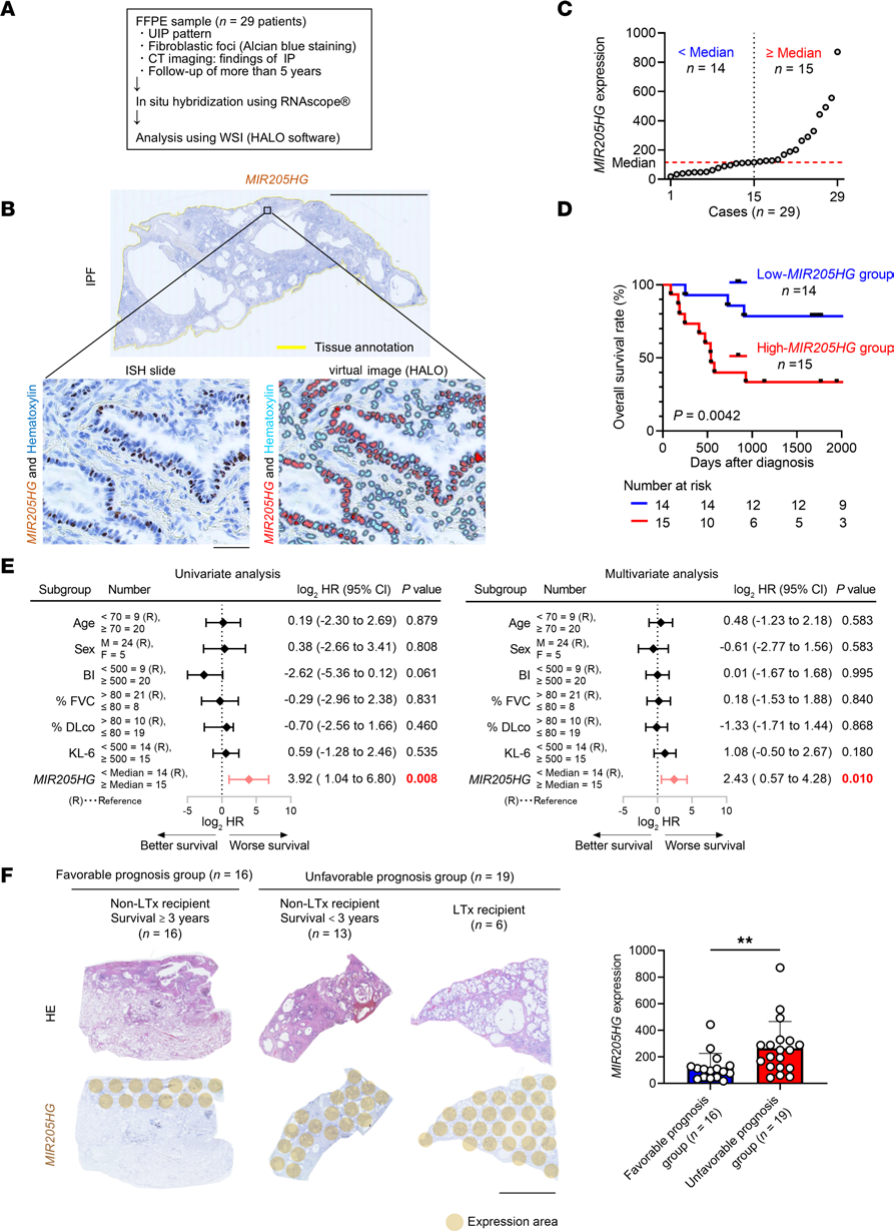
lncRNA *MIR205HG* is an independent poor prognostic factor in patients with IPF. (**A**) Overview of clinical implication assessment based on *MIR205HG* expression in patients with IPF (*n* = 29). UIP, usual interstitial pneumonia. (**B**) Representative images of *MIR205HG* ISH staining in patients with IPF. Scale bar: 10 mm. Zoomed image and virtual composite image after HALO software analysis are shown. Scale bar: 50 μm. (**C**) Plots of *MIR205HG* expression in patients with IPF (*n* = 29). The median was used as a cutoff value. (**D**) Kaplan-Meier curves for OS rate (%) in patients with IPF (*n* = 29) divided into high-*MIR205HG* group (*n* = 15) and low-*MIR205HG* group (*n* = 14). HR, 5.23; 95% CI, 1.80–15.17; *P* = 0.0042. *P* values were determined by log-rank test. (**E**) Forest plots of univariate and multivariate Cox regression analysis in the correlation between *MIR205HG* expression and other clinical factors. *P* values were determined by Cox proportional hazards method. (**F**) Representative of whole image of HE and *MIR205HG* ISH staining. Bar graph of *MIR205HG* expression in patients with IPF of the favorable-prognosis group (survival ≥ 3 years, *n* = 16) and the unfavorable-prognosis group (survival < 3 years, *n* = 13, and lung transplant recipients [LTx recipients], *n* = 6). Scale bar: 10 mm. Data represent mean ± SD. **P < 0.01; *P* values were determined by 2-tailed Mann-Whitney *U* test.

To assess the clinical implications of *MIR205HG* in IPF, we conducted in situ hybridization (ISH) on 29 samples from patients with IPF (Figure 2A). The expression level of *MIR205HG* was scored using HALO software (Figure 2B), and patients were divided into a high-*MIR205HG* group (*n* = 15) and a low-*MIR205HG* group (*n* = 14) based on the median value (Figure 2C). Kaplan-Meier analysis revealed that the high-*MIR205HG* group had a significantly lower overall survival (OS) rate than the low-*MIR205HG* group (HR, 5.23; 95% CI, 1.80–15.17; *P* = 0.0042) (Figure 2D). Univariate and multivariate Cox regression analyses further demonstrated that *MIR205HG* was an independent risk factor affecting the OS of patients with IPF (Figure 2E). To reconfirm the significance of *MIR205HG* in the prognosis of IPF, we compared the expression of *MIR205HG* between 2 groups of IPF patients: the favorable-prognosis group (*n* = 16) (survival ≥ 3 years) and the unfavorable-prognosis group (*n* = 19) (survival < 3 years, *n* = 13, and lung transplant recipients, *n* = 6). The expression level of *MIR205HG* was significantly higher in the unfavorable- than favorable-prognosis group (Figure 2F).

### The MIR205HG^+^IL33^+^ cell subset in the alveolar region increases in IPF.

**Figure 3:**
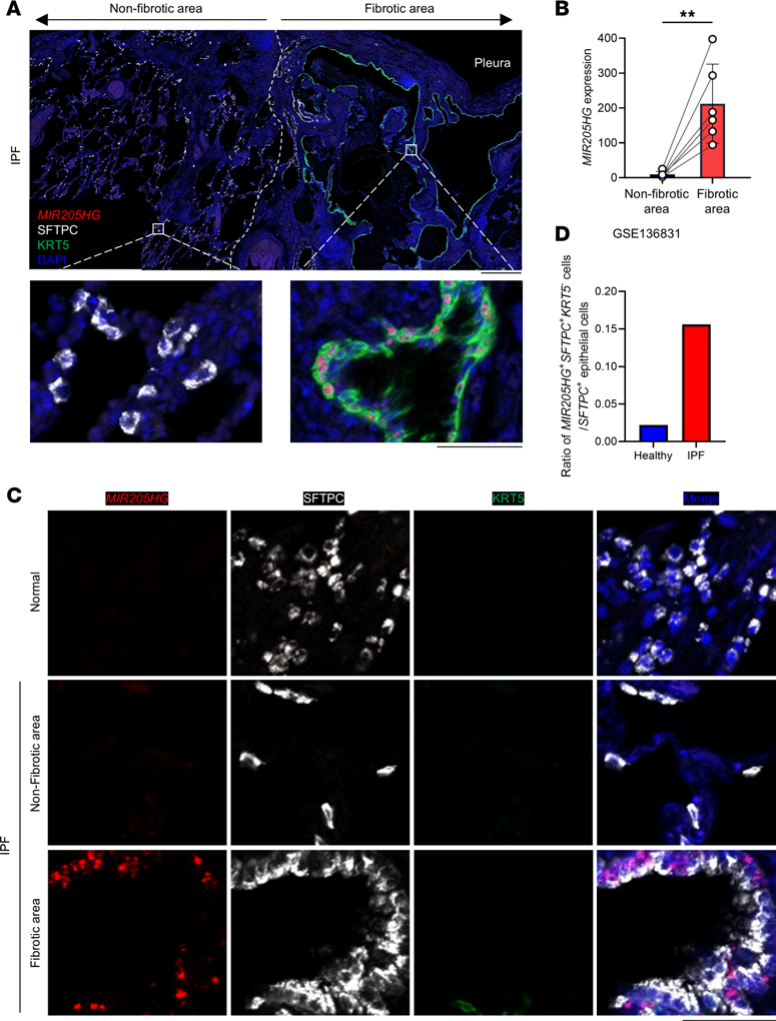
A subset of *MIR205HG*^+^ abnormal AT2 cells is increased in patients with IPF. (**A**) Representative confocal images of *MIR205HG* ISH, SFTPC IHC, and KRT5 IHC staining in nonfibrotic and fibrotic areas of patients with IPF (*n* = 6). Scale bars: 500 μm (upper) and 50 μm (lower). (**B**) Quantification of *MIR205HG* expression in nonfibrotic and fibrotic areas. Data represent mean ± SD. **P < 0.01; *P* values were determined by 2-tailed paired *t* test. (**C**) Representative confocal images of *MIR205HG* ISH, SFTPC IHC, and KRT5 IHC staining in healthy (*n* = 6) and nonfibrotic or fibrotic area of patients with IPF (*n* = 6). Scale bar: 50 μm. (**D**) Ratio of *MIR205HG*^+^*SFTPC*^+^*KRT5*^–^ cells in *SFTPC*^+^ epithelial cell population in healthy and IPF lungs. Public scRNA-Seq data (GSE136831) were used for analysis.

To clarify the detailed spatial expression of *MIR205HG* in IPF, we conducted *MIR205HG* ISH combined with IHC for SFTPC (an AT2 cell marker) and KRT5 (a basal cell marker). The *MIR205HG* ISH signal exhibited a nuclear staining pattern and was predominantly detected in KRT5^+^ cells within fibrotic areas but not in SFTPC^+^ cells of nonfibrotic areas (Figure 3A). The number of *MIR205HG* ISH signal-positive cells was higher in fibrotic areas than in nonfibrotic areas (Figure 3B).

Notably, we found that *MIR205HG*^+^ cells were detected in the alveolar region and that these cells were also positive for SFTPC, indicating that abnormal AT2 cells expressing *MIR205HG* were present in the alveolar region of patients with IPF (Figure 3C). These *MIR205HG*^+^ abnormal AT2 cells were negative for KRT5, and such cells were not detected in the nonfibrotic areas of patients or in alveolar normal lungs (Figure 3, A and C, and Supplemental Figure 3A). Consistent with this finding, public scRNA-Seq data (GSE136831) revealed that the proportion of *MIR205HG*^+^ abnormal AT2 cells was higher in IPF than in healthy lungs (Figure 3D).

**Figure 4:**
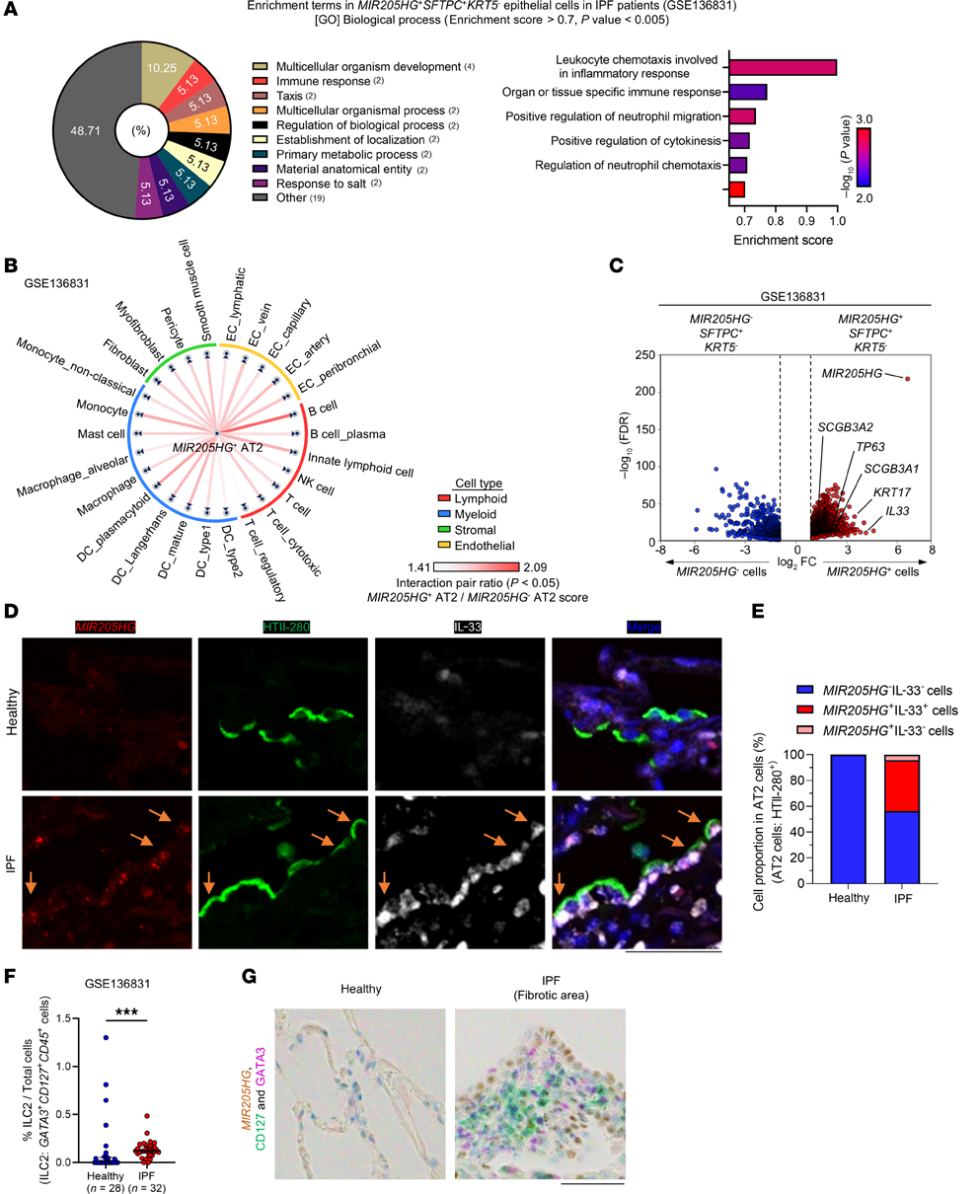
A subset of *MIR205HG*^+^ abnormal AT2 cells express IL-33 and exist in close proximity to ILC2s in patients with IPF. (**A**) GO analysis of biological processes upregulated in *MIR205HG*^+^*SFTPC*^+^*KRT5*^–^ epithelial cells compared with *MIR205HG*^–^*SFTPC*^+^*KRT5*^–^ epithelial cells. Pie chart showing 39 processes with significant differences. The parentheses indicate the number of enrichment terms that belong to the process. Significant differences were determined using an enrichment score > 0.7, *P* < 0.005. Bar graph showing 6 inflammation-related processes among 39 processes. (**B**) Cell–cell communication network showing the ratio of receptor–ligand pairs between the *MIR205HG*^+^*SFTPC*^+^*KRT5*^–^ cells (*MIR205HG*^+^ AT2 cells) and other meta cell types, classified as lymphoid, myeloid, stromal, and endothelial cell clusters (Figure 1B) compared with *MIR205HG*^–^*SFTPC*^+^*KRT5*^–^ cells (*MIR205HG*^–^ AT2 cells). *P* values were determined by the permutation test. (**C**) Volcano plot of DEGs in *MIR205HG*^–^*SFTPC*^+^*KRT5*^–^ cells and *MIR205HG*^+^*SFTPC*^+^*KRT5*^–^ cells. The cutoff values were log_2_FC > 1, FDR < 0.05. (**D**) Representative confocal images of *MIR205HG* ISH, HTII-280 IHC, and IL-33 IHC staining in control (*n* = 3) and IPF patients (*n* = 3). Orange arrows indicate *MIR205HG*^+^HTII-280^+^IL-33^+^ AT2 cells. Scale bar: 50 μm. (**E**) Quantification of AT2 cells expressing *MIR205HG* and IL-33 in healthy and IPF patients in **D**. (**F**) Number of *CD127*^+^*GATA3*^+^*CD45*^+^ cells in healthy (*n* = 28) and IPF (*n* = 32) lungs. Bars represent the median and 95% CI. **P < 0.01; *P* values were determined by 2-tailed Mann-Whitney *U* test. (**G**) Representative images of *MIR205HG* ISH, CD127 IHC, and GATA3 IHC staining in control (*n* = 4) and IPF patients (*n* = 4). Scale bar: 50 μm. (**A**, **B**, **E**, and **F**) Public scRNA-Seq data (GSE136831) were used for analysis.

Gene ontology (GO) analysis indicated that signatures involved in the inflammatory response were mainly enriched in *MIR205HG*^+^ AT2 cells relative to *MIR205HG*^–^ AT2 cells in IPF (Figure 4A). We then constructed a putative cell–cell communication network by mapping known receptor–ligand pairs across cell types. *MIR205HG*^+^ AT2 cells were revealed to interact with a variety of immune cells, including B cells, monocytes, and innate lymphoid cells (Figure 4B and Supplemental Figure 3B). Among the cytokines and chemokines inducing these immune cells, the *IL33* gene was identified as the most DEG, being highly expressed in *MIR205HG*^+^ AT2 cells (Figure 4C). Another scRNA-Seq dataset (GSE159354) (21) also revealed that *MIR205HG*^+^ AT2 cells found in IPF expressed high levels of *IL33* mRNA (Supplemental Figure 3, C and D). Indeed, *MIR205HG*^+^HTII-280^+^ (another AT2 marker) cells expressed IL-33 protein in IPF, whereas such *MIR205HG*^+^HTII-280^+^IL-33^+^ cells were not detected in healthy lungs (Figure 4, D and E). IL-33 is known to induce ILC2s (22–24), and scRNA-Seq data (GSE136831) showed that *CD127*^+^*GATA3*^+^ ILC2s were significantly increased in IPF (Figure 4F). Furthermore, we found *CD127^+^GATA3^+^* ILC2s in proximity to *MIR205HG*^+^ cells (Figure 4G), suggesting that *MIR205HG*^+^ abnormal AT2 cells in IPF may be involved in the regulation of ILC2s via IL-33.

### Overexpression of lncRNA MIR205HG increases IL33 mRNA expression in alveolar organoids.

**Figure 5:**
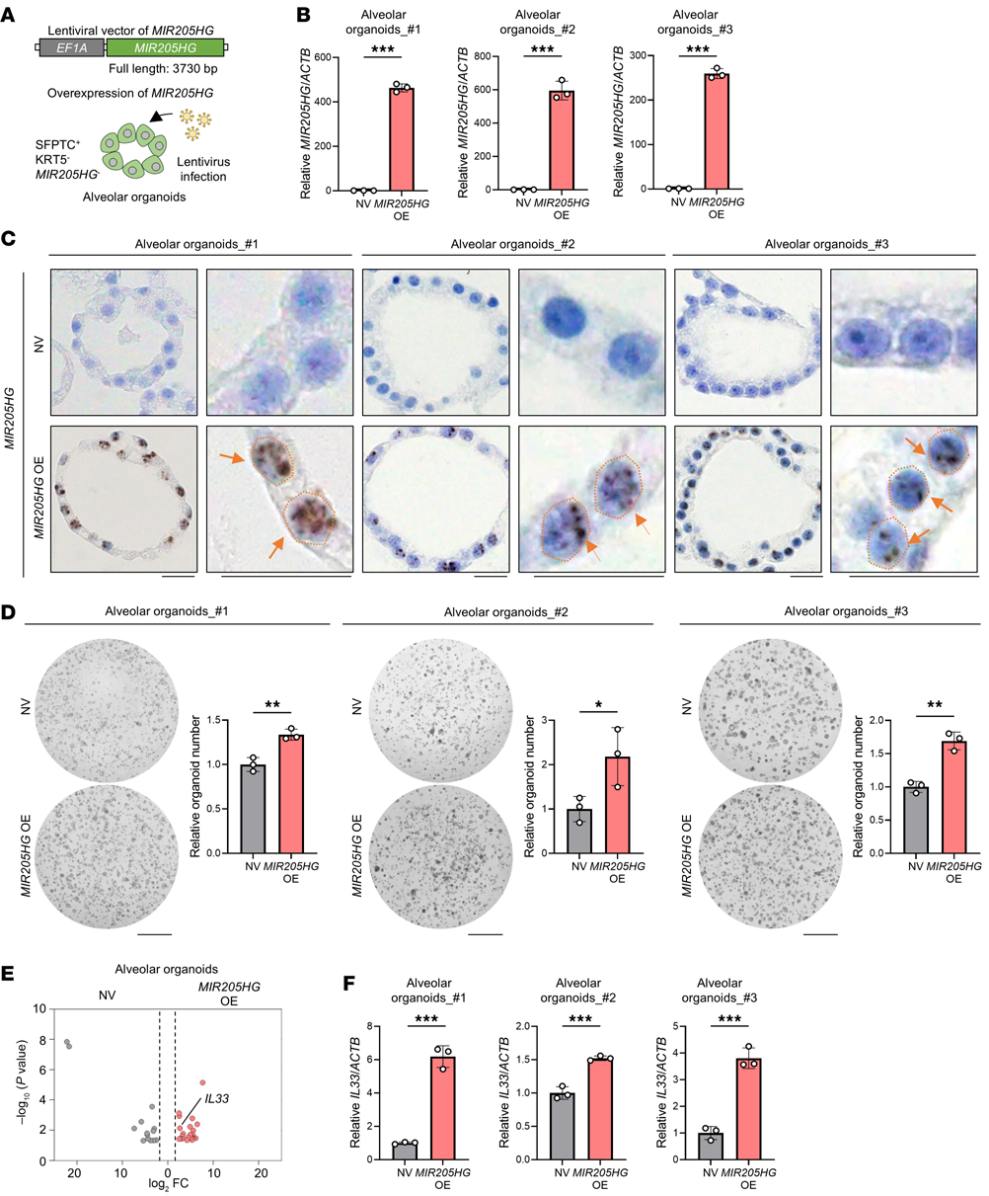
Overexpression of lncRNA *MIR205HG* upregulates *IL33* mRNA in alveolar organoids. (**A**) Schematic of experimental design for identification of *MIR205HG*-regulated genes in *MIR205HG*-OE alveolar organoids. (**B**) qRT-PCR showing *MIR205HG* expression in negative vector (NV) and *MIR205HG*-OE alveolar organoids. (**C**) Representative images of *MIR205HG* ISH staining in NV and *MIR205HG*-OE alveolar organoids. Orange arrows indicate *MIR205HG* signals, which are detected in nuclei (circled by orange dotted line). Scale bar: 20 μm. (**D**) Quantification of organoid number in NV and *MIR205HG*-OE alveolar organoids. Scale bar: 1 mm. (**E**) Volcano plot of DEGs in NV and *MIR205HG*-OE alveolar organoids. Top 40 DEGs are shown. The *IL33* gene is indicated among upregulated genes in *MIR205HG*-OE alveolar organoids. The cutoff values were log_2_FC > 2, *P* < 0.05. (**F**) qRT-PCR showing *IL33* expression in NV and *MIR205HG*-OE alveolar organoids. (**B**, **D**, and **F**) Data represent mean ± SD. *P < 0.05, **P < 0.01, ***P < 0.001; *P* values were determined by 2-tailed *t* test.

To more precisely examine the role of *MIR205HG* in IL-33 expression, we established alveolar organoids from the healthy lungs of 3 independent cases (Supplemental Figure 4A). The established alveolar organoids expressed AT2 cell markers (SFTPC and HTII-280) but not basal cell markers, including *MIR205HG*, as verified by IHC and ISH (Supplemental Figure 4, B and C). We then overexpressed *MIR205HG* in the established alveolar organoids, creating *MIR205HG*-overexpressing (*MIR205HG*-OE) alveolar organoids (Figure 5, A–C). When *MIR205HG* was overexpressed, the number of organoids increased compared with the control (Figure 5D). Next, we performed RNA-Seq (GSE275717) in *MIR205HG*-OE alveolar organoids generated from 3 independent cases. As observed in basal cells (NGFR^+^) cells (Supplemental Figure 2G), we confirmed that *MIR205HG*-OE alveolar organoids have transcripts of *MIR205HG* (Supplemental Figure 5A). GO analysis revealed that processes related to the inflammatory response were enriched in the upregulated genes of the *MIR205HG*-OE alveolar organoids (Supplemental Figure 5B). Among the inflammatory response genes, *IL33* was consistently enriched in *MIR205HG*-OE alveolar organoids (Figure 5E and Supplemental Figure 5C). Indeed, quantitative reverse transcription polymerase chain reaction (qRT-PCR) revealed a significant increase in *IL33* mRNA expression in *MIR205HG*-OE alveolar organoids (Figure 5F). In addition to genetic manipulation such as overexpression, the correlation of *MIR205HG* and *IL33* expression was evaluated by treatment with reagents. We searched for reagents and found that bleomycin treatment increased *MIR205HG* expression. When treated with bleomycin, *IL33* was highly expressed as well as *MIR205HG* (Supplemental Figure 4D). These findings were consistent with the earlier results showing that the *IL33* gene was highly expressed in *MIR205HG*^+^ AT2 cells but not in *MIR205HG*^–^ AT2 cells.

**Figure 6:**
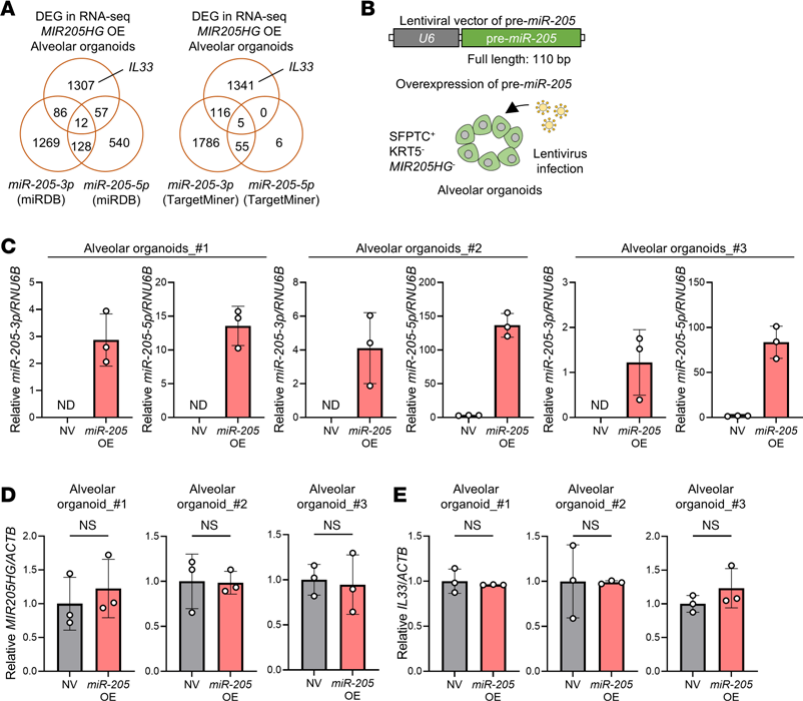
Overexpression of pre–*miR-205* does not upregulate *IL33* mRNA in alveolar organoids. (**A**) Venn diagram showing DEGs of *MIR205HG*-OE alveolar organoids (upper) and target genes of *miR-205* with miRDB (26) (lower left) and TargetMiner (27) (lower right) datasets. (**B**) Schematic of experimental design for identification of pre–*mir-205* regulated genes in *miR-205*–OE alveolar organoids. (**C**) qRT-PCR showing *miR-205-3p* and *miR-205-5p* expression in NV and *miR-205–*OE alveolar organoids. Data represent mean ± SD. N.D., not detected. (**D**) qRT-PCR showing *MIR205HG* expression in NV and *miR-205*–OE alveolar organoids. (**E**) qRT-PCR showing *IL33* expression in NV and *miR-205*–OE alveolar organoids. (**D** and **E**) Data represent mean ± SD. *P* values were determined by 2-tailed *t* test.

Because transcripts of *MIR205HG* contained the region coding *microRNA-205* (*miR-205*) (25), it is possible that *miR-205* but not *MIR205HG* affects *IL33* expression. To exclude this possibility, we investigated public data from miRDB (26) and TargetMiner (27); we did not find *IL33* among the target genes of *miR-205-3p* and *miR-205-5p* (Figure 6A and Supplemental Figure 6). For experimental validation, we overexpressed pre–*miR-205* in alveolar organoids (*miR-205*–OE alveolar organoids) (Figure 6B). Even when *miR-205-3p* and *miR-205-5p* were overexpressed (Figure 6C), the expression levels of *MIR205HG* (Figure 6D) and *IL33* (Figure 6E) were not affected. These results indicated that the lncRNA *MIR205HG* is involved in the increased expression of *IL33* and that *miR-205* is not involved in this process.

### Knockdown of MIR205HG reduces IL33 mRNA and IL33 protein expression levels in basal cells.

**Figure 7:**
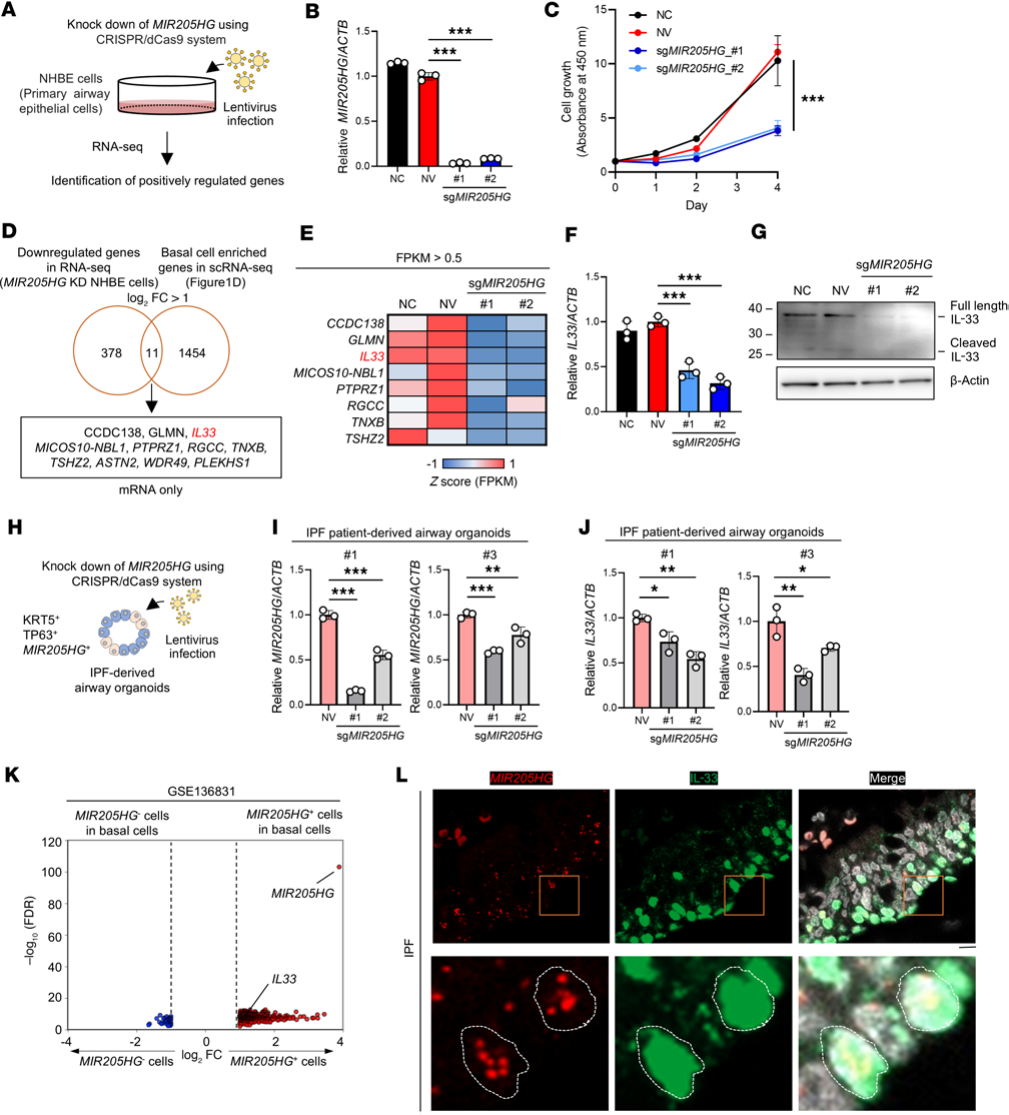
Downregulation of *MIR205HG* decreases *IL33* mRNA and IL-33 protein expression in basal cells. (**A**) Experimental procedure for the identification of genes positively regulated by *MIR205HG* in NHBE cells using the CRISPR interference/dCas9-KRAB (CRISPR/dCas9) system. (**B**) qRT-PCR showing *MIR205HG* expression in NV and *MIR205HG*-KD NHBE cells. NC, negative control. (**C**) Cell growth assay in NV and *MIR205HG*-KD NHBE cells. (**D**) Venn diagram showing downregulated genes in *MIR205HG*-KD NHBE cells (bulk RNA-Seq, left) and basal cell enriched genes in Figure 1D (scRNA-Seq, right). The cutoff values were log_2_FC > 1 (bulk RNA-Seq and scRNA-Seq). Two common mRNAs are listed below. (**E**) Heatmap showing common mRNAs that are downregulated genes in *MIR205HG-*KD NHBE cells and basal cell enriched genes shown in **D**. Fragments per kilobase of exon per million mapped fragment (FPKM) > 0.5 mRNAs were visualized. (**F**) qRT-PCR showing *IL33* mRNA expression in NV and *MIR205HG*-KD NHBE cells. (**G**) Western blot showing IL-33 protein expression in NV and *MIR205HG*-KD NHBE cells. (**H**) Schematic of experimental design for identification of *MIR205HG*-regulated genes in IPF patient–derived airway organoids using the CRISPR/dCas9 system. (**I**) qRT-PCR showing *MIR205HG* expression in NV and *MIR205HG*-KD IPF patient–derived airway organoids. (**J**) qRT-PCR showing *IL33* mRNA expression in NV and *MIR205HG*-KD of IPF patient–derived airway organoids. (**K**) Volcano plot of DEGs in *MIR205HG*^–^ basal cell and *MIR205HG*^+^ basal cell in public scRNA-Seq data (GSE136831). The cutoff values were log_2_FC > 1, FDR < 0.05. (**L**) Representative images of *MIR205HG* ISH and IL-33 IHC staining in patients with IPF. Scale bar: 10 μm. (**B**, **C**, **F**, **I**, and **J**) Data represent mean ± SD. *P < 0.05, **P < 0.01, ***P < 0.001; *P* values were determined by 1-way ANOVA with Holm-Šídák post hoc test.

To clarify the relationship between *MIR205HG* and *IL33* expression, we knocked down *MIR205HG* in human bronchial epithelial (NHBE) primary cells exhibiting the basal cell phenotype using the CRISPR interference/dCas9-KRAB (CRISPR/dCas9) system (Figure 7A and Supplemental Figure 7, A and B). The knockdown of *MIR205HG* (*MIR205HG*-KD) was verified in NHBE cells by qRT-PCR and ISH (Figure 7B and Supplemental Figure 7C). *MIR205HG*-KD significantly reduced cell proliferation in NHBE cells (Figure 7C).

We performed RNA-Seq (GSE275709) on *MIR205HG*-KD NHBE cells. Among 1,465 genes highly expressed in basal cells identified by scRNA-Seq (Figure 1D, GSE136831), 11 genes were downregulated after *MIR205HG*-KD: *CCDC138*, *GLMN*, *IL33*, *MICOS10-NBL1*, *PTPRZ1*, *RGCC*, *TNXB*, *TSHZ2*, *ASTN2*, *WDR49*, and *PLEKHS1* (Figure 7, D and E). Among them, 4 genes (*IL33*, *PLEKHS1*, *PTPRZ1*, and *RGCC*) were upregulated in the *MIR205HG*-OE alveolar organoids. These findings indicate that the expression level of *IL33* was correlated with that of *MIR205HG*. Indeed, the downregulation of *IL33* mRNA and IL-33 protein in *MIR205HG*-KD cells was verified by qRT-PCR, Western blot, and IHC (Figure 7, F and G, and Supplemental Figure 7D).

To validate these results, we established IPF patient–derived airway organoids that expressed basal cell markers (Supplemental Figure 8, A–C), and *MIR205HG*-KD was performed in these organoids using the CRISPR/dCas9 system (Figure 7H). The qRT-PCR results revealed that *IL33* mRNA was downregulated in *MIR205HG*-KD IPF patient–derived airway organoids (Figure 7, I and J). Consistent with this finding, public scRNA-Seq data (GSE136831) showed enrichment of *IL33* expression in *MIR205HG*^+^ basal cells (Figure 7K). Histologically, we found that basal cells and aberrant basaloid cells with high *MIR205HG* expression coexpressed IL-33 protein in patients with IPF (Figure 7L and Supplemental Figure 8D). These results indicate that *MIR205HG* is responsible for regulating IL-33 expression in basal cells as well as alveolar cells affected by IPF.

### Fused in sarcoma RNA-binding protein stabilizes MIR205HG and IL33 mRNA in NHBE cells and IPF patient–derived airway organoids.

**Figure 8:**
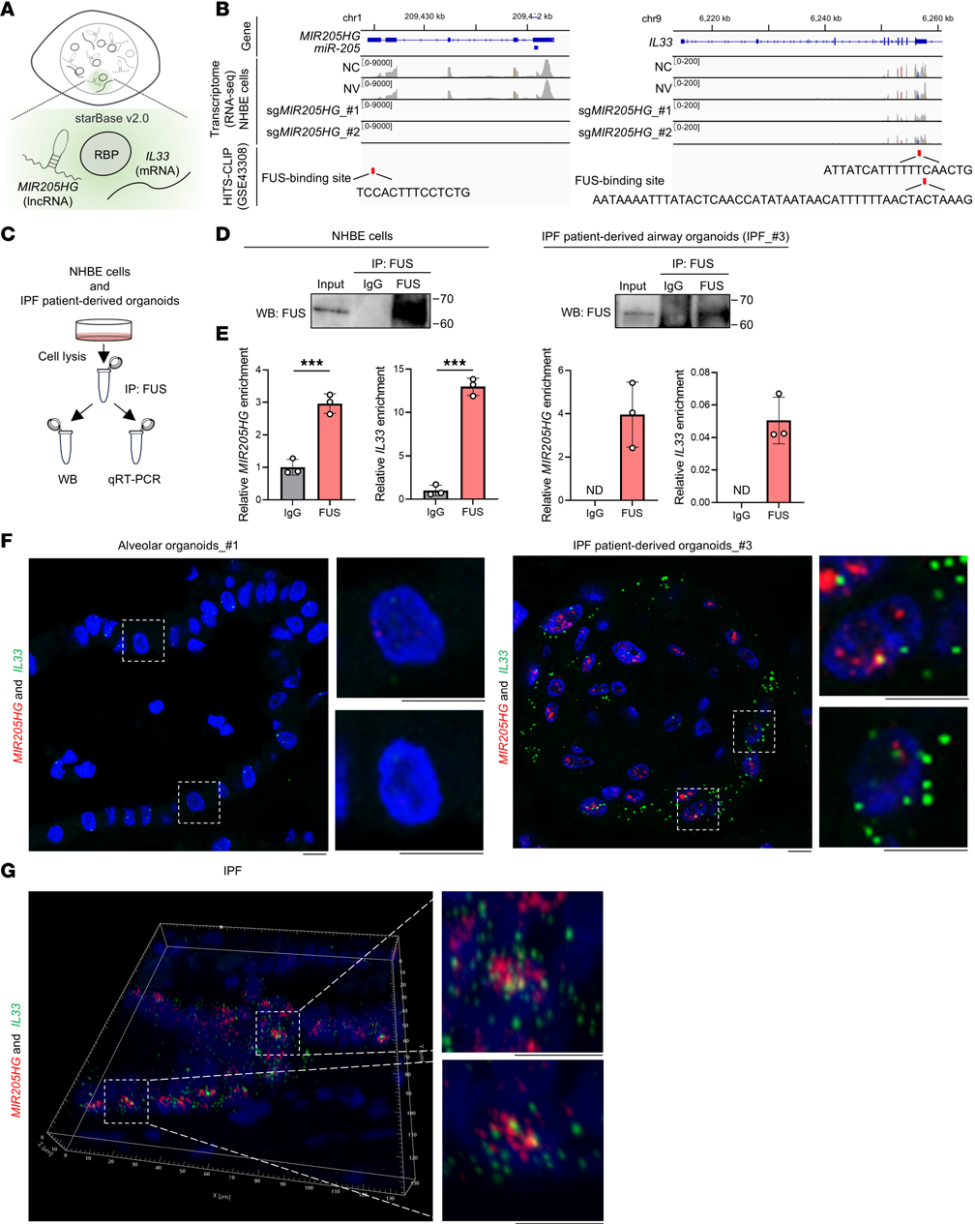
*MIR205HG* and *IL33* mRNA binds to fused in sarcoma RBP in NHBE cells and IPF patient–derived airway organoids. (**A**) Schematic illustration for the identification of RBPs common to *MIR205HG* and *IL33* using starBase v2.0 (32). (**B**) Integrative Genomics Viewer (IGV) showing *MIR205HG* and *IL33* loci in NV and *MIR205HG-*KD NHBE cells. The motif sequences of the respective *MIR205HG* and *IL33* recognized by the FUS protein are shown. Motif sequences were obtained from public HITS-CLIP dataset (GSE43308) (33). (**C**) RIP workflow to examine the binding of the respective *MIR205HG* and *IL33* to the FUS protein. Immunoprecipitation (IP) was performed using an FUS antibody and an IgG antibody as control. RNA enrichment in the FUS antibody was calculated using the IgG antibody as control. (**D**) Western blot showing FUS protein expression in FUS IP using NHBE cells and IPF patient–derived airway organoids. (**E**) qRT-PCR showing *MIR205HG* and *IL33* in FUS RIP using NHBE cells and IPF patient–derived airway organoids. N.D., not detected. Data represent mean ± SD. ***P < 0.001; *P* values were determined by 2-tailed *t* test. (**F**) Representative images of *MIR205HG* and *IL33* double ISH staining in alveolar organoids and IPF patient–derived airway organoids. Scale bar: 10 μm. (**G**) Representative images of *MIR205HG* and *IL33* double ISH staining in IPF tissue samples. Scale bar: 10 μm.

We next investigated the molecular mechanisms by which *MIR205HG* regulates IL-33 expression. Because several known lncRNAs cooperate with RNA-binding proteins (RBPs) (28–31), we searched for RBPs commonly bound by *MIR205HG* and *IL33* RNA using starBase v2.0 (32) (Figure 8A). In the public HITS-CLIP dataset (GSE43308) (33), fused in sarcoma (FUS) was identified as an RBP that binds to both *MIR205HG* and *IL33* mRNA (Figure 8B). The FUS-binding sequence on *MIR205HG* did not contain the *miR-205* locus (Figure 8B).

We then performed RNA-immunoprecipitation (RIP) in NHBE cells and IPF patient–derived airway organoids (Figure 8C). FUS protein–enriched samples contained significantly higher amounts of *MIR205HG* and *IL33* mRNA relative to control IgG samples in both NHBE cells and IPF patient–derived airway organoids (Figure 8, D and E, and Supplemental Figure 9, A and B). In IPF patient–derived airway organoids, *MIR205HG* and *IL33* double ISH staining revealed that *MIR205HG* was localized in proximity to *IL33* mRNA (Figure 8F). This observation was also found in patients with IPF (Figure 8G).

**Figure 9:**
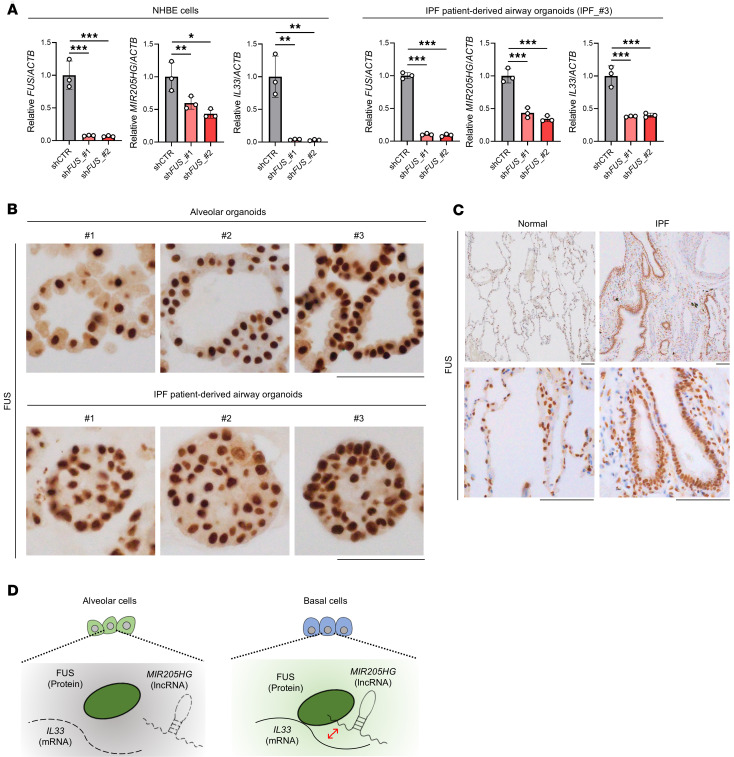
Knockdown of FUS protein reduces expression of *MIR205HG* and *IL33* mRNA. (**A**) qRT-PCR showing *FUS*, *MIR205HG*, and *IL33* expression of *FUS*-KD NHBE cells and IPF patient–derived airway organoids using shRNA. Data represent mean ± SD. *P < 0.05, **P < 0.01, ***P < 0.001; *P* values were determined by 1-way ANOVA with Holm-Šídák post hoc test. (**B**) Representative images of FUS IHC staining in alveolar organoids and IPF patient–derived airway organoids. Scale bar: 100 μm. (**C**) Representative images of FUS IHC staining in healthy and IPF tissue samples. Scale bar: 100 μm. (**D**) Schematic illustration showing predictions from the experimental results.

Because FUS protein contributes to RNA stabilization (34–36), we hypothesized that FUS-KD could affect the amounts of *MIR205HG* and *IL33* mRNA. As expected, FUS-KD significantly decreased *MIR205HG* and *IL33* mRNA amounts in NHBE cells and IPF patient–derived airway organoids, indicating the role of FUS in stabilizing *MIR205HG* and *IL33* mRNA (Figure 9A and Supplemental Figure 9C). We then hypothesized that *MIR205HG* regulates *IL33* mRNA expression by affecting FUS expression. To examine this hypothesis, we investigated the relationship between FUS expression and the amounts of *MIR205HG* and *IL33* mRNA. However, *FUS* mRNA and FUS protein expression were not affected in *MIR205HG*-KD NHBE cells (Supplemental Figure 9, D and E). Moreover, there was no difference in *FUS* mRNA and FUS protein expression in IPF patient–derived airway organoids with high *MIR205HG* and IL-33 expression relative to alveolar organoids with low *MIR205HG* and IL-33 expression (Figure 9B and Supplemental Figure 9, F and G). Additionally, IHC showed that FUS protein was expressed in almost all cells, including the alveolar and airway epithelium, with no obvious difference in FUS expression between healthy lungs and patients with IPF (Figure 9C). Therefore, it is unlikely that *MIR205HG* affects *IL33* mRNA expression by modulating FUS expression. Instead, FUS protein itself stabilized *MIR205HG* and *IL33* mRNA, pointing to another mechanism such as a direct interaction between *MIR205HG* and *IL33* mRNA (Figure 9D).

### The AluJb element of MIR205HG is essential for regulation of IL33 expression.

**Figure 10:**
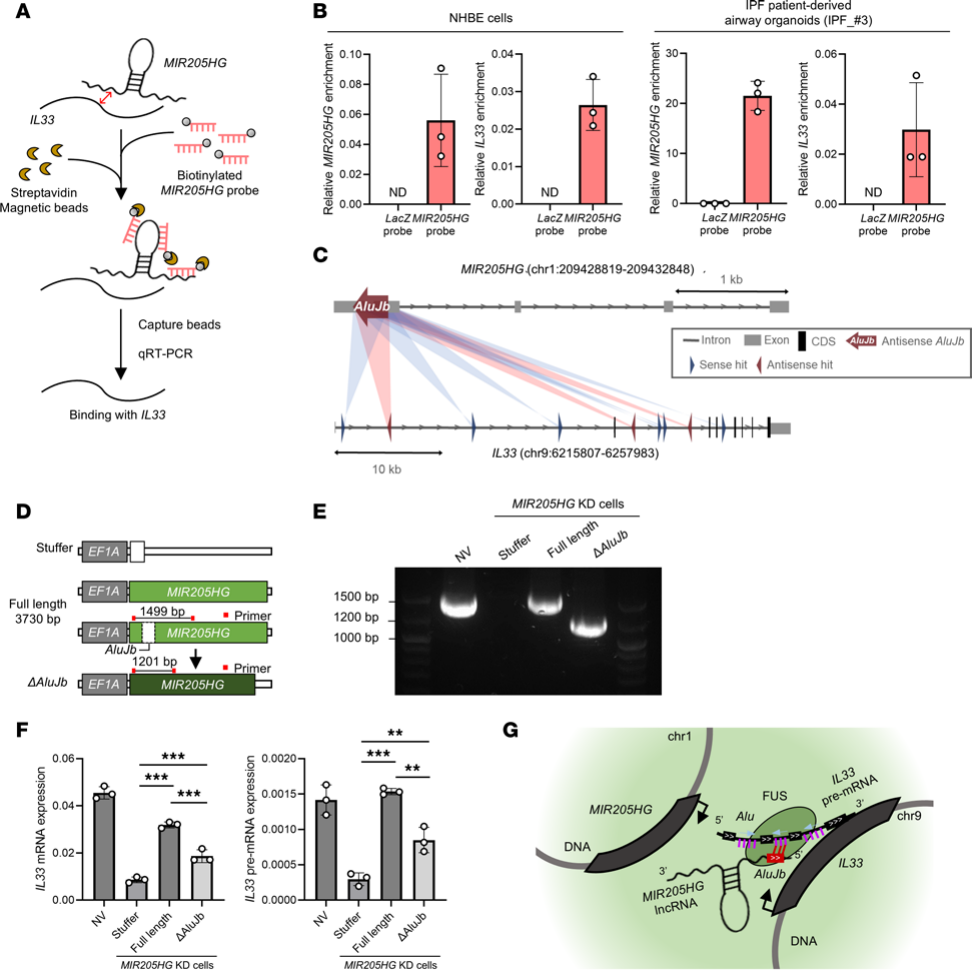
*AluJb* element of *MIR205HG* regulates *IL33* expression. (**A**) Workflow of ChIRP for binding of *IL33* using the *MIR205HG* probe. (**B**) qRT-PCR of *MIR205HG* and *IL33* in ChIRP for *MIR205HG* enrichment in NHBE cells and IPF patient–derived airway organoids. ChIRP was performed using *MIR205HG* probe and *LacZ* probe as control. Data represent mean ± SD. N.D., not detected. (**C**) Predicted binding sites of the *AluJb* element of *MIR205HG* and the *Alu* elements (intron) of *IL33*. A total of 9 sites with similarity to *AluJb* or *Alu* elements were found in the *Alu* element of *IL33*. Blue and red indicate sequence similarity between the *AluJb* element of *MIR205HG* and the sense/antisense strand of the *Alu* elements of *IL33*, respectively. (**D**) Vector design for functional analysis of the *AluJb* element of *MIR205HG*. (**E**) RT-PCR products obtained by transfection of NHBE cells with primers shown in **D**. Deletion of the *AluJb* element (Δ*AluJb*) of *MIR205HG* was confirmed. (**F**) qRT-PCR showing *IL33* mRNA and *IL33* pre-mRNA expression under **E** conditions. Data represent mean ± SD. **P < 0.01, ***P < 0.001; *P* values were determined by 1-way ANOVA with Holm-Šídák post hoc test. (**G**) Schematic illustration of the experimental results.

To investigate the direct interaction between *MIR205HG* and *IL33* mRNA, we performed chromatin isolation via RNA precipitation (ChIRP) experiments (Figure 10A). In these ChIRP experiments, the *MIR205HG* probe coprecipitated more *IL33* mRNA compared with the control *LacZ* probe in NHBE cells and IPF patient–derived airway organoids (Figure 10B). We next examined sequence similarity between *MIR205HG* and *IL33* in genes enriched in basal cells (1,465 genes). The sequence similarity between these 2 is among the top 20% of the 1,465 genes (Supplemental Figure 10, A and B). When searching for motifs of sequence similarity, the *AluJb* element of *MIR205HG* was found to possess similarity to 9 (6 sense and 3 antisense) sites of *Alu* elements located in the intron region of the *IL33* gene (Figure 10C and Supplemental Figure 11A). These *Alu* elements were not found in the exon regions of the *IL33* gene, indicating that *IL33* pre-mRNA but not *IL33* mRNA possessed these elements. Because *Alu* elements have been reported to interact with each other (37–39), it is possible that the *AluJb* element of *MIR205HG* may be responsible for the interaction between *MIR205HG* and *IL33* pre-mRNA.

We then investigated whether the *AluJb* element of *MIR205HG* affected *IL33* mRNA amount (Figure 10, D and E). When the full-length *MIR205HG* vector was transfected into *MIR205HG*-KD NHBE cells, it restored *IL33* mRNA and pre-mRNA amounts (Figure 10F). In contrast, the Δ*AluJb* element vector, in which the *AluJb* element of *MIR205HG* was deleted, did not upregulate *IL33* mRNA and pre-mRNA amounts compared to full-length vector (Figure 10F), indicating that the *AluJb* element of *MIR205HG* was responsible for regulating IL-33 mRNA amount. Hi-C public data showed that *MIR205HG* (located on chromosome 1) and *IL33* gene (located on chromosome 9) were in close genomic proximity in cells expressing *MIR205HG* and *IL33* mRNA (Supplemental Figure 11B). These results suggest that *MIR205HG* might stabilize *IL33* pre-mRNA through its *AluJb* element immediately after the transcription of the *IL33* gene, thereby upregulating *IL33* mRNA amounts (Figure 10G).

### Small molecule DQzG targets the AluJb element of MIR205HG and reduces IL-33 expression.

**Figure 11:**
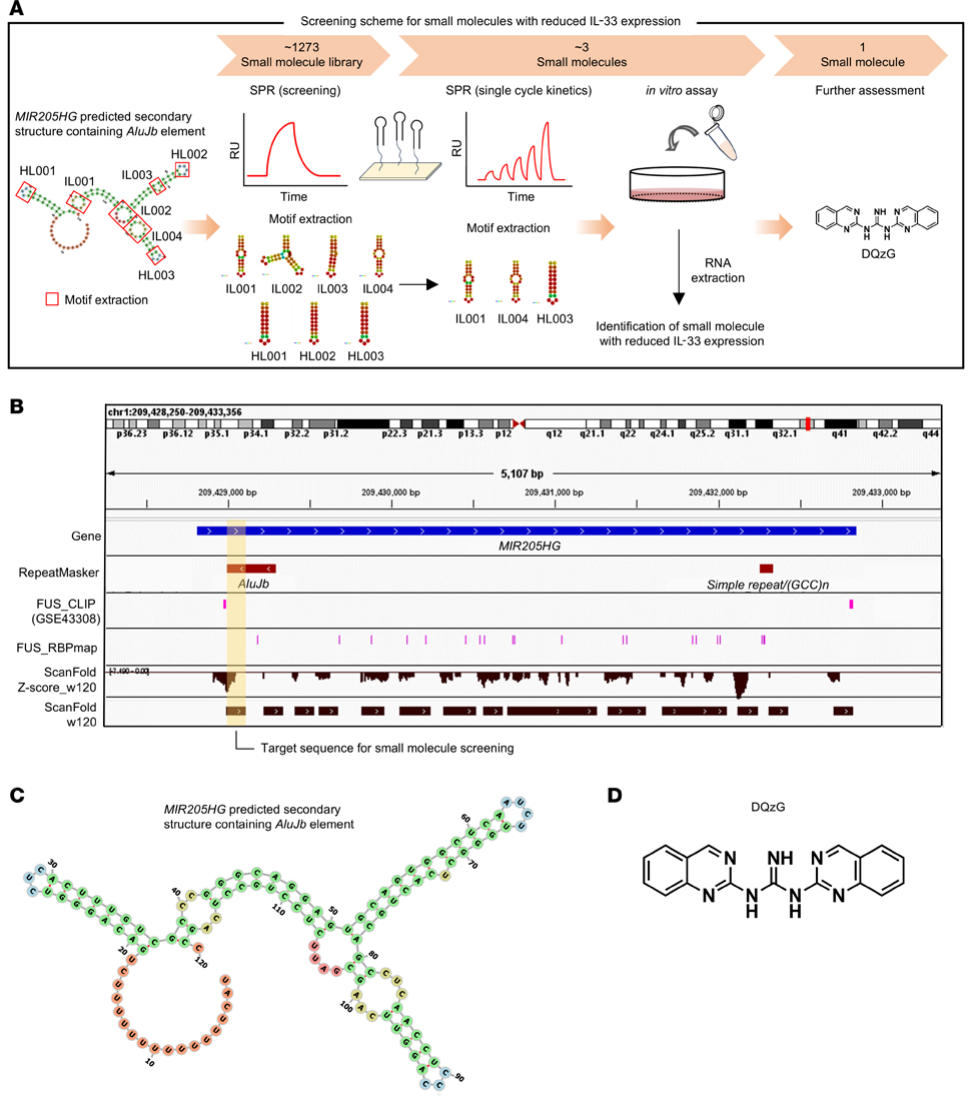
Screening of small molecules that reduce IL-33 expression by targeting the *AluJb* element. (**A**) Overview of the screening scheme for the identification of small molecules that reduce *IL33* expression. surface plasmon resonance (SPR) experiments were carried out on 7 motifs from the *MIR205HG* predicted secondary structure containing the *AluJb* element. The SPR experiments identified 3 small molecules (ANP77, DQzG, and TO239) from a library of 1,273 small molecules. These 3 small molecules were assessed for *IL33* expression in NHBE cells. (**B**) IGV plot showing the target sequence region for small molecule screening. The target sequence was selected as the yellow background region containing the *AluJb* element. (**C**) Predicted secondary structure containing *AluJb* element of *MIR205HG*. RNA secondary structure was predicted with RNAfold and visualized with forna. (**D**) The structure of the small molecule DQzG.

Furthermore, to complement the relevance of the *AluJb* element of *MIR205HG*, we explored small molecules that could target the *AluJb* element to inhibit IL-33 expression (Figure 11A). To identify binding molecules using SPR, we selected 7 internal and hairpin loop motifs from the secondary structure predicted by CentroidFold (40) and designed SPR-measurable RNA sequences (Figure 11, B and C, and Supplemental Figure 12A). SPR-based screening with a library of 1,273 small molecules identified ANP77 (41), DQzG (42), and TO239 as promising candidates that showed strong binding to the *AluJb* element (Supplemental Figure 12, A and B). These molecules were then screened for their suppressive effect on *IL33* expression in NHBE cells; DQzG exhibited the greatest inhibitory effect on *IL33* mRNA expression (Figure 11D and Supplemental Figure 12C). Nuclear magnetic resonance (NMR) and SPR analysis at multiple concentrations further supported the ability of DQzG to bind to the *AluJb* element (Supplemental Figure 12, B and D).

**Figure 12:**
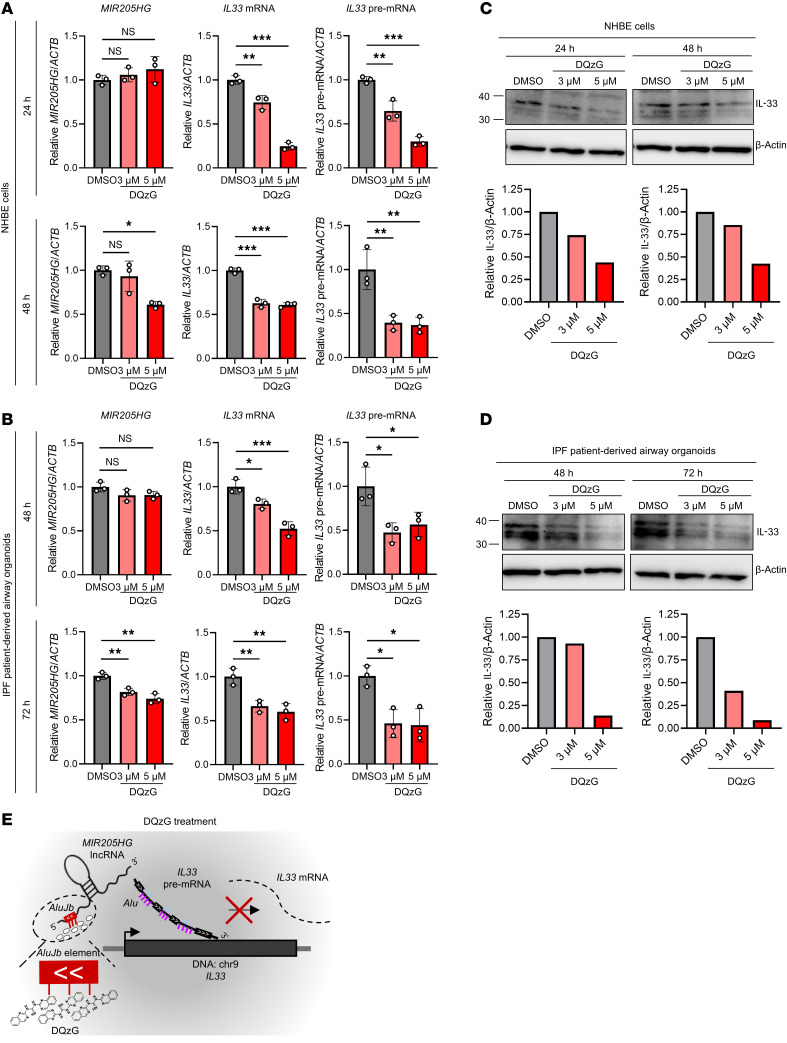
Small molecule DQzG targets the *AluJb* element of *MIR205HG* and reduces IL-33 expression. (**A** and **B**) qRT-PCR showing *MIR205HG*, *IL33* mRNA, and *IL33* pre-mRNA expression in NHBE cells (24 hours and 48 hours) and IPF patient–derived airway organoids (48 hours and 72 hours) after 3 μM and 5 μM DQzG treatment. Data represent mean ± SD. *P < 0.05, **P < 0.01, ***P < 0.001; *P* values were determined by 1-way ANOVA with Holm-Šídák post hoc test. (**C** and **D**) Western blot showing IL-33 protein expression in NHBE cells (24 hours and 48 hours) and IPF patient–derived airway organoids (48 hours and 72 hours) after 3 μM and 5 μM DQzG treatment. IL-33 expression levels in the western blot were quantified using ImageJ software (NIH). (**E**) Schematic illustration of the experimental results.

We comprehensively examined DEGs in NHBE cells and IPF patient–derived airway organoids treated with DQzG using RNA-Seq analysis. DQzG treatment identified several DEGs and identified *IL33* as one of the genes common to both NHBE cells and IPF patient–derived airway organoids (Supplemental Figure 13A). We verified time- and concentration-dependent suppression of *IL33* mRNA, *IL33* pre-mRNA, and IL-33 protein in NHBE cells and IPF patient–derived airway organoids (Figure 12, A–D). Interestingly, *MIR205HG* expression was also slightly reduced in NHBE cells and IPF patient–derived airway organoids following DQzG treatment (Figure 12, A and B). We verified that cell numbers were not affected under these conditions (Supplemental Figure 13B). As a control experiment, we treated NHBE cells and IPF patient–derived airway organoids with DNpG, a small molecule without binding that does not bind to the sequence in the *AluJb* element recognized by DQzG. Treatment with DNpG had no effect on *IL33* mRNA, *IL33* pre-mRNA, or *MIR205HG* expression (Supplemental Figure 14, A–C). Taken together, these results highlight the important role of the *AluJb* element of *MIR205HG* in the regulation of *IL33* expression (Figure 12E).

### The high-MIR205HG group shows high IL-33 expression and increased number of ILC2s compared with the low-MIR205HG group in IPF.

We investigated the clinical significance of elevated IL-33 expression in IPF. IL-33 IHC was performed on the same healthy lungs (*n* = 15) and patients with IPF (*n* = 29) as used in Figure 2A (Supplemental Figure 15A). The results revealed that IL-33 protein expression was significantly higher in IPF than in normal lungs (Supplemental Figure 15B). However, IL-33 protein expression was comparable between the low-*MIR205HG* and high-*MIR205HG* groups used in Figure 2C (Supplemental Figure 15C). Additionally, Kaplan-Meier analysis showed that IL-33 expression was not associated with the OS rate when the low–IL-33 group was compared with the high–IL-33 group (Supplemental Figure 15, D and E).

**Figure 13:**
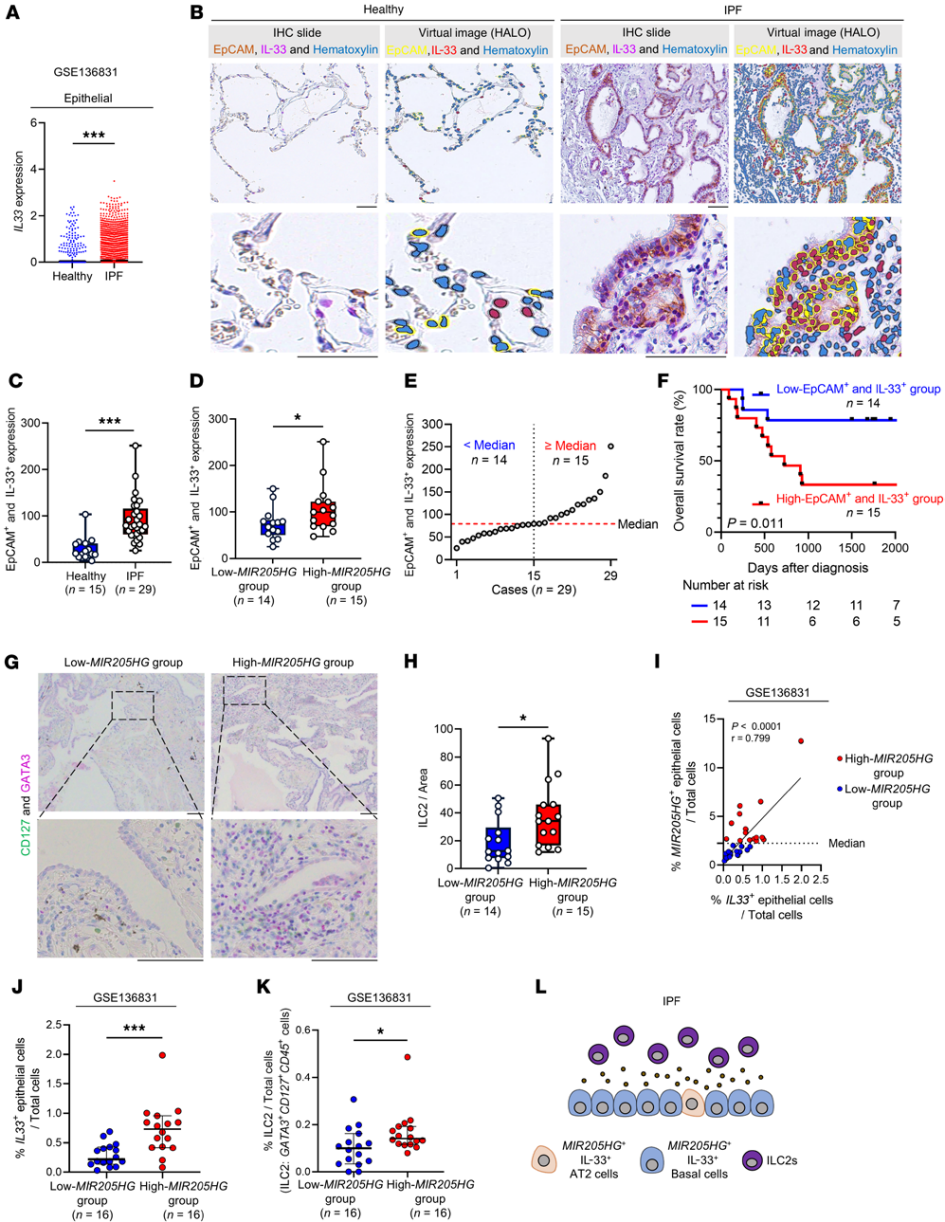
The high-*MIR205HG* group exhibits high IL-33 expression and increased number of ILC2s compared with the low-*MIR205HG* group in patients with IPF. (**A**) Plot of *IL33* expression for healthy lungs (*n* = 28) and patients with IPF (*n* = 32) in epithelial cell cluster. (**B**) Representative images of EpCAM IHC and IL-33 IHC staining in healthy lungs (*n* = 15) and patients with IPF (*n* = 29) in the cohort from Supplemental Figure 15A. Scale bar: 100 μm. (**C**) Plot of EpCAM^+^ and IL-33^+^ expression in **B**. (**D**) Plot of EpCAM^+^ and IL-33^+^ expression in high-*MIR205HG* group (*n* = 15) and low-*MIR205HG* group (*n* = 14). The 2 groups based on expression of *MIR205HG* in Figure 1I were used. (**E**) Plots of EpCAM^+^ and IL-33^+^ expression in patients with IPF (*n* = 29). The median was used as the cutoff value of EpCAM^+^ and IL-33^+^ expression. (**F**) Kaplan-Meier curves for OS rate (%) in patients with IPF (*n* = 29) divided into high-EpCAM^+^ and IL-33^+^ group (*n* = 15) and low-EpCAM^+^ and IL-33^+^ group (*n* = 14). HR, 4.49; 95% CI, 1.57–12.86; *P* = 0.0011; *P* values determined by log-rank test. (**G**) Representative images of CD127 IHC and GATA3 IHC staining in high-*MIR205HG* group (*n* = 15) and low-*MIR205HG* group (*n* = 14). Scale bar: 100 μm. (**H**) Plot of number of ILC2s (CD127^+^ and GATA3^+^) in **G**. *P < 0.05; *P* values determined by 2-tailed Student’s *t* test. (**I**) Correlation analysis of *MIR205HG*^+^ and *IL33*^+^ epithelial cells using patients with IPF (*n* = 32). Pearson’s *r* = 0.799 and *P* < 0.0001; *P* values are 2 sided. (**J**) Plot of *IL33*^+^ epithelial cells in high-*MIR205HG* group (*n* = 16) and low-*MIR205HG* group (*n* = 16). (**K**) Number of ILC2s (*CD127*^+^*GATA3*^+^*CD45*^+^ cells) in high-*MIR205HG* group (*n* = 16) and low-*MIR205HG* group (*n* = 16). (**L**) Schematic illustration of the experimental results. (**A**, **C**, **D**, **J**, and **K**) *P < 0.05, ***P < 0.001; *P* values were determined by 2-tailed Mann-Whitney *U* test. (**J** and **K**) Bars represent the median and 95% CI. (**A** and **I**–**K**) Public scRNA-Seq data (GSE136831) were used for analysis.

UMAP analysis of scRNA-Seq data (GSE136831) showed that *IL33* mRNA expression was detected in various cells, such as epithelial cells, fibroblasts, and endothelial cells (Supplemental Figure 15, F–H). Previous studies have reported that increased IL-33 expression in the epithelium contributes to long-term innate immune activity and pathogenesis in diseases such as chronic obstructive pulmonary disease and emphysema (43–46). When healthy lungs were compared with IPF patient scRNA-Seq data (GSE136831), the difference in the *IL33* expression level was most pronounced in epithelial clusters (Figure 13A and Supplemental Figure 15I). Consequently, we focused on epithelial IL-33 expression. In the same samples as in Supplemental Figure 15A, double IHC staining with EpCAM (an epithelial marker) and IL-33 was performed (Figure 13B). The number of EpCAM^+^ and IL-33^+^ cells was significantly higher in IPF than in normal lungs (Figure 13C). Furthermore, the number of EpCAM^+^ and IL-33^+^ cells was higher in the high-*MIR205HG* group than in the low-*MIR205HG* group (Figure 13D). We divided the cases into 2 groups: those with high EpCAM^+^ and IL-33^+^ epithelial cells, and those with low EpCAM^+^ and IL-33^+^ epithelial cells (Figure 13E). Kaplan-Meier analysis showed that the former group had significantly lower OS rates than the latter group (HR, 4.49; 95% CI, 1.57–12.86; *P* = 0.011) (Figure 13F).

Finally, we examined the relationship between *MIR205HG*-expressing cells and ILC2s. The number of CD127^+^GATA3^+^ ILC2s was immunohistochemically examined in the low-*MIR205HG* group and the high-MIR205HG group (Figure 13G). The results revealed that the number of CD127^+^GATA3^+^ ILC2s was significantly higher in the high-*MIR205HG* group than in the low-*MIR205HG* group (Figure 13H). Additionally, the expression of *MIR205HG* and *IL33* in epithelial cells was plotted for patients with IPF (*n* = 32), demonstrating a positive correlation between *MIR205HG* and *IL33* expression in epithelial cells using public scRNA-Seq data (GSE136931) (Figure 13I). When these 32 patients with IPF were divided into low-*MIR205HG* (*n* = 16) and high-*MIR205HG* (*n* = 16) groups, the high-*MIR205HG* group showed significantly increased expression of *IL33* in epithelial cells and increased numbers of ILC2s (Figure 13, J–L). In summary, we revealed that *MIR205HG* plays a crucial role in regulating epithelial IL-33 expression and is involved in the pathogenesis of IPF.

## Discussion

IPF is histologically characterized by the appearance of basal cells in alveolar regions, but evidence on their involvement in the pathogenesis of IPF is limited. This paper demonstrates that *MIR205HG*, a lncRNA highly expressed in basal cells, contributes to the pathogenesis of IPF. *MIR205HG* was upregulated in IPF compared with healthy lungs and was identified as an independent poor prognostic factor in IPF. Through overexpression and knockdown of *MIR205HG* using primary cells and organoids, we demonstrated that *MIR205HG* regulates IL-33 expression. As for the regulatory mechanism, we clarified that the *AluJb* element of *MIR205HG* impacts *IL33* expression through direct interaction. Moreover, we showed that DQzG, a small molecule targeting the *AluJb* element of *MIR205HG*, suppressed IL-33 expression. IL-33 is known to strongly induce ILC2s, which are important cells in the pathogenesis of IPF (47). Indeed, a positive correlation between *MIR205HG*-regulated IL-33 expression and the number of ILC2s was observed in patients with IPF. This work provides insight that *MIR205HG* contributes to IPF progression through IL-33 expression. However, this study did not evaluate in vivo whether *MIR205HG* expression enhances fibrosis by affecting IL-33 expression and the number of ILC2s. We investigated whether *Mir205hg* is upregulated in bleomycin treatment, a commonly used model of pulmonary fibrosis. However, we did not detect *Mir205hg* expression by ISH in control and bleomycin-treated mice. Moreover, public RNA-Seq data analysis showed that *Mir205hg* expression was not induced under different conditions of bleomycin treatment compared to controls (Supplemental Figure 16, A and B). Additionally, an *AluJb* element was identified in human *MIR205HG* but not in mouse *Mir205hg* because the insertion of the *AluJb* element into the *MIR205HG* gene occurred in a common ancestor of the Haplorhini (Supplemental Figure 16C). It has been reported that mouse models of bleomycin-induced fibrosis do not resemble human pathogenesis, which may explain why our findings cannot be validated in the mouse (48, 49). In the future, we should explore animal models of pulmonary fibrosis that reflect human pathogenesis and validate our proposed mechanism.

The pathology of IPF is characterized by spatially and temporally heterogeneous pulmonary remodeling (2). From our detailed histological analysis of IPF samples, we found cells with high *MIR205HG* expression levels in fibrotic regions of IPF. Most *MIR205HG*^+^ cells were positive for the basal cell marker KRT5, whereas a limited number of *MIR205HG*^+^ cells were positive for the AT2 markers SFTPC and HTII-280. *MIR205HG*^+^ AT2 cells were in close proximity to *MIR205HG*^–^ AT2 cells in fibrotic regions, which may represent an early stage of IPF pathogenesis. Previous reports have demonstrated that abnormally differentiated AT2 cells promote lung fibrosis (50) and are involved in the pathogenesis of IPF (7, 51–54). According to public scRNA-Seq data, *MIR205HG*^+^ AT2 cells highly expressed *KRT17*, *TP63*, *SCGB3A1*, and *SCGB3A2* compared with *MIR205HG*^–^ AT2 cells (Figure 4C). These findings suggest that *MIR205HG*^+^ AT2 cells may represent abnormally differentiated AT2 cells during transformation to basal cells, referred to as alveolar-basal intermediates (53), aberrant basaloid cells (7), AT0 (54), and terminal and respiratory bronchiole secretory cells (54). Unfortunately, *MIR205HG* overexpression did not increase the expression of basal cell markers, such as *KRT17* and *TP63*, in established alveolar organoids, indicating that the transformation to basal cells is a more complex molecular process. Although the proportion of *MIR205HG*^+^ AT2 cells was low in the IPF sample, these cells expressed IL-33, and ILC2s were found around them. In support of reports that abnormal AT2 cells contribute to pathogenesis, we may have uncovered a role for *MIR205HG*^+^ abnormal AT2 cells that induce various inflammatory responses and contribute to the progression of IPF.

IL-33 is expressed in various cell types other than the epithelium, such as fibroblasts and vascular endothelial cells (55). Consistent with this pattern, our IHC analysis demonstrated that IL-33 was expressed in various cell types, including epithelial cells. We divided IPF cases into high– and low–IL-33 groups but detected no difference in prognosis between these 2 groups. Public scRNA-Seq data revealed that the difference in *IL33* expression levels between healthy lungs and patients with IPF was most pronounced in the epithelial cell cluster. We then counted IL-33–positive epithelial cells (EpCAM^+^ cells) and divided patients with IPF into cases with highly IL-33–expressing epithelial cells and lowly IL-33–expressing epithelial cells. Cases with high–IL-33–expressing epithelial cells showed a worse prognosis relative to cases with low–IL-33–expressing epithelial cells. The relationship between IL-33 and lung fibrosis has been reported in mice, in which fibroblast-derived IL-33 is important in fibrosis (47). The controversy over the cell types responsible for fibrosis as the IL-33 source might be due to species differences. Indeed, the expression pattern of IL-33 differs between humans and mice; for example, IL-33 is detected in mouse alveolar epithelium (56, 57) but not in human alveolar epithelium.

Overexpression of *MIR205HG* increased *IL33* expression, and its knockdown reduced *IL33* expression. The transfection of full-length *MIR205HG* vector increased *IL33* expression in *MIR205HG*-KD NHBE cells. When *MIR205HG* deleting the *AluJb* element (Δ*AluJb* element) vector was transfected, the increase in *IL33* expression was attenuated. These findings indicate that the *AluJb* element of *MIR205HG* was responsible for *IL33* expression. Although the function of *Alu* elements is not fully understood, recent analyses have revealed that *Alu* elements are involved in various transcriptional regulations (38). The *AluJb* element of *MIR205HG* is essential for transcriptional regulation of genes specific for luminal cells of the prostate (37). *MIR205HG* binds to the *Alu* element in the promoter region of luminal genes via its *AluJb* element and interferes with the binding of interferon-regulatory factor (IRF) that transactivates luminal specific genes. Deletion of the *AluJb* element of *MIR205HG* abolishes the occupation of *MIR205HG*, allowing IRF to bind to the promoter region of luminal genes (37). These reports suggest the importance of direct interaction between *Alu* elements in transcriptional regulation. With respect to the *IL33* gene, there are 9 *Alu* elements possessing sequence similarity to the *AluJb* element of *MIR205HG*. In this context, our results suggest that the *AluJb* element of *MIR205HG* plays a functional role through direct interaction with the *Alu* element of *IL33* in the regulation of IL-33 expression.

The *AluJb* element of *MIR205HG* is present in both genomic DNA and the lncRNA itself. When *MIR205HG*-KD was carried out, we utilized the CRISPR/dCas9 system but not the CRISPR/Cas9 system. In this method, *MIR205HG*-coding genomic DNA remains, but transcription from the locus is abolished. Because the downregulation of *IL33* mRNA amount was detected in this knockdown, the *AluJb* element of *MIR205HG* lncRNA, but not of genomic DNA, was essential for *IL33* transcriptional regulation.

Analysis of the public HITS-CLIP data identified FUS as an RBP that binds to both *MIR205HG* and *IL33*. Knockdown of FUS downregulated the amounts of *MIR205HG* and *IL33* mRNA. However, FUS is also present in cells without *MIR205HG* and *IL33* mRNA, indicating that FUS itself does not transactivate *MIR205HG* and *IL33* mRNA. RBPs, including FUS, contribute to RNA stabilization (34–36). These findings suggest that FUS stabilizes *MIR205HG* and *IL33* mRNA. The *Alu* elements were located in the intron of the *IL33* gene but not in the exon. Therefore, *IL33* pre-mRNA possessed *Alu* elements, whereas *IL33* mRNA did not. The Hi-C analysis showed a close genomic distance between the *MIR205HG* and *IL33* loci. *IL33* pre-mRNA, immediately after transcription from the *IL33* locus, may interact with *MIR205HG* via *Alu* elements. As an RBP binding to both *MIR205HG* and *IL33*, FUS may provide a niche enabling interactions with both RNAs.

Finally, we attempted to find a small molecule that could reduce IL-33 expression by disrupting the interaction between *Alu* elements. ANP77, DQzG, and TO239 were selected as small molecules binding to the *AluJb* element through SPR experiments. Among the 3, DQzG exhibited the most inhibitory effect on IL-33 expression. In contrast, DNpG, a small molecule that does not bind to the *AluJb* element recognized by DQzG, did not inhibit *IL33* expression, suggesting that DQzG reduced *IL33* expression by inhibiting the interaction between *Alu* elements.

## Methods

### Sex as a biological variable.

Sex was not considered as a biological variable.

### Patient tissue samples.

IPF tissues were obtained from 29 patients who underwent surgery or video-assisted thoracoscopic surgery at Osaka University Hospital between 2012 and 2017. Lung transplant tissues for IPF were obtained from 6 patients at Osaka University Hospital between 2012 and 2020. Pathologically, all IPF samples showed a usual interstitial pneumonia pattern with fibroblastic foci positive for Alcian blue staining (catalog 4085-2, Muto Pure Chemicals). Patients who were followed up for at least 5 years were included. The OS time of enrolled patients ranged from 3 to 103 months (median, 56 months). Normal lung tissues were obtained from 14 patients with normal background lungs undergoing tumor resection.

### Antibodies.

Details of the information concerning antibodies (clone, catalog, dilution, source) are summarized in Supplemental Table 1.

### IHC staining.

FFPE tissues and organoids were cut at a thickness of 4 μm, deparaffinized in xylene, dehydrated in 100% ethanol, and then rinsed in water. IHC staining was conducted using the Dako Autostainer Link 48 (Agilent Technologies), in accordance with the manufacturer’s instructions. The slides were incubated with the indicated primary antibody for 1 hour, followed by the indicated secondary antibody for 1 hour. Details of the antibodies are described in Supplemental Table 1. Signals were detected by staining with DAB (catalog SK005, Agilent Technologies), Stayright Purple (catalog 45906, AAT Bioquest), Ventana DISCOVERY Purple Kit (catalog 760-229, Roche), or Ventana DISCOVERY Green Kit (catalog 760-271, Roche), followed by counterstaining with hematoxylin.

### RNA ISH staining.

FFPE tissue and organoid sections were cut at a thickness of 4 μm, then subjected to ISH staining using the RNAscope 2.5 HD Detection Reagents-BROWN Kit (catalog 322300, Advanced Cell Diagnostics), in accordance with the manufacturer’s instructions. Detailed methods are described in the Supplemental Methods.

### Scoring of IHC and ISH staining.

Stained slides were scanned and imaged with the NanoZoomer 2.0-HT (Hamamatsu Photonics) at ×20 or ×40 original magnification. Whole-slide images were analyzed using HALO v3.5 software (Indica Labs). The expression scores for *MIR205HG* ISH staining, IL-33 single IHC staining, EpCAM and IL-33 double IHC staining, and CD127 and GATA3 (ILC2s) double IHC staining were calculated by dividing the number of positive cells by the tissue area (mm^2^).

### Public data analysis of scRNA-Seq and bulk RNA-Seq.

Public scRNA-Seq data were downloaded from GSE136831 (7) and GSE159354 (21) in the NCBI GEO public database. The datasets were reanalyzed and visualized using BioTuring Single Cell Browser (BBrowser) software. For cell–cell interaction analysis, we used the raw gene count data from GSE136831 and applied the statistical analysis method of CellPhoneDB v2.0 (58) with the CellPhoneDB-data v5.0 database to predict enriched receptor–ligand interactions. Bulk RNA-Seq datasets were downloaded from the GEO datasets GSE92592 (19) and GSE124685 (20); *MIR205HG* expression levels were compared between healthy controls and IPF patient samples.

### Establishment of human lung organoids.

Human lung organoids (alveolar organoids and IPF patient–derived airway organoids) were obtained from patients who underwent surgery at the Department of Thoracic Surgery at Osaka University Hospital. All organoids were established and maintained at 37°C in a 5% CO_2_ atmosphere as previously described, with modifications (59). Clinical data for all organoids are summarized in Supplemental Table 3. Detailed methods are described in the Supplemental Methods. To isolate AT2 and basal cells, the cells were sorted using a FACSAria III (BD Biosciences). Isolated AT2 cells and basal cells were embedded in Matrigel (catalog 356231, Corning), seeded at 10,000 cells per well (48-well plate, catalog 677180, Greiner Bio-One), and cultured in 250 μL of lung organoid medium per well. The organoid media are summarized in Supplemental Table 4.

### Generation of MIR205HG or pre–miR-205 overexpression in alveolar organoids.

Lentiviral production of the following vectors was carried out according to the lentiviral production method, using the plasmids pLV[ncRNA]-EGFP-EF1A>{hMIR205HG[NC_000001.11]}_whole sequencing (catalog VB220908-1312ftg, Vector Builder), pLV-EGFP-EF1A>ORF_stuffer (referred to as “NV”) (catalog VB900124-3812qdj, Vector Builder), and pre–*miR-205* (catalog VB230626-1545krg, Vector Builder). Single-cell pellets of alveolar organoid were prepared; the cells were suspended with 10 μL of lentiviral suspension and incubated for 10 minutes. Matrigel was then added to the tubes at a volume of 20 μL/well and spread onto a 48-well plate.

### Generation of MIR205HG-KD NHBE cells and IPF patient–derived airway organoids using the CRISPR/dCas9 system.

sgRNAs targeting human *MIR205HG* were designed using the CRISPR design website CRISPick (https://portals.broadinstitute.org/gppx/crispick/public). The following *MIR205HG*-targeting sequences were used in this study: sg*MIR205HG*_*#*1: 5′-GGACTCAGCCCATTTCAAGG-3′ and sg*MIR205HG*_#2: 5′-GCAAGTCAAGGGTGAGCAAGA-3′. sgRNAs were inserted into the BsmBI/Esp3I (catalog FD0454, Thermo Fisher Scientific) sites of the pLV hU6-sgRNA hUbC-dCas9-KRAB-T2a-Puro vector (catalog 71236, Addgene). Each vector plasmid was transformed into STBL3 (catalog C737303, Thermo Fisher Scientific) and spread on Luria-Bertani agar plates. After 20 hours of incubation, a single colony was selected and propagated in Luria-Bertani medium (catalog 20068-75, Nacalai Tesque) for 24 hours. The plasmid DNA was then purified using the NucleoBond Xtra Maxi EF kit (catalog 2403002M, Takara), in accordance with the manufacturer’s recommendations. Knockdown of *MIR205HG* was verified by qRT-PCR.

### Generation of FUS-KD NHBE cells and IPF patient–derived airway organoids using the shRNA system.

The following 2 shRNA plasmids targeting the human *FUS* gene were provided by the Center for Medical Research and Education, Graduate School of Medicine, Osaka University: MISSION shRNA #1 (catalog TRCN0000001134, target sequence: CCGGGCCTGGGTGAGAATGTTACAACTCGAGTTGTAACATTCTCACCCAGGCTTTTT, Clone ID: NM_008599.4-276s21c1, referred to as “sh*FUS*_#1”) and MISSION shRNA #2 (catalog TRCN0000001133, target sequence: CCGGCGTGGTGGCTTCAATAAATTTCTCGAGAAATTTATTGAAGCCACCACGTTTTT, Clone ID: NM_008599.4-276s21c1, referred to as “sh*FUS*_#2”) (Merck). The nontarget shRNA control plasmid (referred to as “shCTR”), MISSION pLKO.1-puro (catalog SHC002, Merck), was used. Knockdown of *FUS* was verified by qRT-PCR.

### Generation of plasmid cells with MIR205HG full-length or AluJb element deletion (ΔAluJb) in MIR205HG-KD NHBE cells.

Lentiviral production of the following vectors was carried out according to the lentiviral production method described above, using the plasmids pLV[ncRNA]-EGFP-EF1A>{h*MIR205HG*[NC_000001.11]}_whole sequencing (referred to as “Full length”) (Vector Builder), pLV-EGFP-EF1A>ORF_stuffer (referred to as “Stuffer”) (Vector Builder), and pLV[ncRNA]-EGFP-EF1A>{h*MIR205HG*[NC_000001.11]}_*AluJb* element deletion (−298 bp) (referred to as “*ΔAluJb*”) (catalog VB231012-1778ges, Vector Builder). *MIR205HG*-KD NHBE cells were infected with the above lentiviral vectors. Target sequences were confirmed by RT-PCR or qRT-PCR.

### Analysis of RBP–RNA interaction.

We queried the starBase v2.0 (32) database (RBP–Target, type = lncRNA, database = hg19) and found that the HITS-CLIP data for FUS (GSE43308) (33) support interactions between FUS and *MIR205HG*, as well as between FUS and *IL33*. The data were retrieved on June 12, 2023.

### RIP.

RIP assays were performed with 0.6 μg/μL anti-FUS and 0.6 μg/μL rabbit IgG whole molecule antibody (catalog 011-000-003, Jackson ImmunoResearch). Briefly, 2.0 × 10^7^ cells were collected and lysed in ice-cold lysis buffer (1% NP-40, 20 mM Tris-HCl, 150 mM NaCl, and 1 mM EDTA) containing cOmplete Protease Inhibitor Cocktail (Roche) and PhosSTOP (Roche). The lysate was sonicated for 10 seconds and incubated for 30 minutes. The cell lysate was harvested by centrifugation at 10,000*g*, followed by incubation in reaction buffer (50 mM Tris-HCl pH 7.5, 5 M NaCl, and TBS-Tween) with the indicated antibodies and Dynabeads Protein G (catalog DB10003, Veritas) at 4°C overnight. The next day, the FUS–RNA complexes were washed 3 times with PBS. Proteins were denatured for Western blot. RNA was extracted and quantified by qRT-PCR. *ACTB* was used as an internal control.

### ChIRP.

ChIRP was performed as previously described (60). Approximately 2.0 × 10^7^ cells were immediately cross-linked in 1% paraformaldehyde/PBS (catalog 09154-85, Nacalai Tesque) for 15 minutes at room temperature, and the cross-linking reaction was stopped by adding 1.5 M glycine (catalog 17109-35, Nacalai Tesque). The cross-linked cells were then washed twice with ice-cold PBS. After PBS removal, the cells were resuspended in 350 μL of lysis buffer (50 mM Tris-HCl pH 7.0, 10 mM EDTA, 1% SDS, 1 mM PMSF [catalog 10837091001, Roche], cOmplete Protease Inhibitor Cocktail from Roche, and RNAse inhibitor [catalog 10777019, Thermo Fisher Scientific]) and disrupted using a Bioruptor II Type 12 (Sonicbio) (30 seconds ON, 30 seconds OFF, 120 cycles). Subsequently, the insoluble fraction was removed by centrifugation at 10,000*g* for 10 minutes at 15°C, and the supernatant was used for ChIRP. Two volumes of hybridization buffer (750 mM NaCl, 1% SDS, 50 mM Tris-HCl pH 7.0, 1 mM EDTA, 15% v/v formamide, 1 mM PMSF, cOmplete Protease Inhibitor Cocktail, and RNAse inhibitor) and the 500 nM *MIR205HG* ChIRP probe (Supplemental Table 5) (LGC Biosearch Technologies) or 500 nM *LacZ* control probe (catalog 03-307, Merck) were added. The mixture was incubated at 37°C for 4 hours and then at 4°C overnight, mixed with Dynabeads MyOne Streptavidin C1 (catalog 65801D, Thermo Fisher Scientific), and incubated for 60 minutes. The beads were then washed 5 times for 5 minutes at 37°C with 0.8 mL of wash buffer (2× saline sodium citrate and 0.5% SDS). The bound proteins were eluted and reverse–cross-linked by boiling in SDS sample buffer (95°C for 30 minutes). The bound RNAs were eluted by protease K treatment (50°C for 45 minutes) followed by heating (95°C for 10 minutes). RNA was then extracted and analyzed by qRT-PCR. *ACTB* was used as an internal control.

### Small molecule treatments for NHBE cells and IPF patient–derived airway organoids.

NHBE cells and IPF patient–derived airway organoids were seeded with the indicated number of cells 2 days prior to small molecule treatment. ANP77, DQzG, TO239, and DNpG (in-house small molecules) were administered at concentrations of 3 μM and 5 μM. The control was a 0.05% DMSO solution. After small molecule treatment, cells were collected at the indicated time points, and samples were prepared for RNA and protein extraction.

### RNA-Seq.

Reverse transcription to cDNA was performed using the GenNext RamDA-Seq Single Cell Kit (catalog RMD-101, TOYOBO), and the library was prepared using the Nextera XT DNA Library Preparation Kit (catalog FC-131-1096, Illumina). Sequencing was performed on the NovaSeq 6000 platform in a 101+101 base paired-end mode (Illumina). Generated reads were mapped to the human (hg38) reference genome using HISAT2 ver.2.1.0 (https://github.com/DaehwanKimLab/hisat2; commit ID 7e01700). FPKMs were calculated using Cuffdiff 2.2.1 (http://cole-trapnell-lab.github.io/cufflinks/cuffdiff/).

### Sequence similarity analysis between MIR205HG and IL33.

Local sequence alignment was performed to evaluate sequence similarity. *MIR205HG* (hg38, chr1:209428819-209432848) and *IL33* (hg38, chr9:6215807-6257983) were used as the query and subject sequences, respectively, for sequence alignment using the discontinuous MegaBLAST program of the BLAST web (61) with default parameters.

### Statistics.

Statistical differences were evaluated using GraphPad Prism 9 and JMP Pro software version 16.0 (SAS Institute). OS rates were analyzed using a Kaplan-Meier curve, log-rank test, and multivariate analysis based on the Cox proportional hazards method using GraphPad Prism 9. Results are expressed as the mean ± SD of triplicate replicates. *P* < 0.05 were considered statistically significant.

### Study approval.

All experiments were approved by the Ethical Review Board of the Graduate School of Medicine, Osaka University (approval no. 15234), and were conducted in accordance with relevant institutional guidelines and regulations. All animal experimental protocols were approved by the Animal Research Committee of Osaka University (approval no. 05-039-004). This study was performed in accordance with the ethical guidelines of the Declaration of Helsinki.

### Data availability.

Our original RNA-Seq data in this study are available at NCBI GEO under the accession numbers GSE275700 (alveolar organoids and IPF-derived airway organoids), GSE275709 (*MIR205HG*-KD NHBE cells), GSE275717 (*MIR205HG*-OE alveolar organoids), GSE275720 (HTII-280^+^ cells and NGFR^+^ cells) and GSE283175 (DQzG-treated NHBE cells and airway organoids from patients with IPF). Source data are provided with this paper. Previously published sequencing data that were reanalyzed are available under the accession numbers GSE136831 (7), GSE159354 (21), GSE92592 (19), GSE124685 (20), and GSE43308 (33). Raw data are also provided in the Supporting Data Values file for this study.

## Author contributions

TT and E Morii designed the study. TT, CZ, DO, MH, and E Morii analyzed the public data. TT performed most of the in vitro experiments and IHC and ISH staining with assistance from CZ, E Murakami, NF, MK, and ZF. TT, HN, YS, and E Morii collected and analyzed the patient clinical data. HN collected the samples from healthy lungs and patients with IPF, and TT established organoids. TT, DO, and E Morii performed and analyzed the RNA-Seq. E Murakami, AS, YH, and KN performed and analyzed the SPR experiments. RI and GK performed and analyzed the NMR experiments. CZ, E Murakami, NF, MK, SN, TM, GK, MH, TH, and KN provided advice regarding experimental techniques and data interpretation. E Morii supervised the project. TT, CZ, E Murakami, NF, and E Morii wrote the initial draft and edited the paper. All the authors read and approved the final paper.

## Supplementary Material

Supplemental data

Unedited blot and gel images

Supporting data values

## Version 1 03/10/2025

Electronic publication
