# Supplementary material for: Involvement of lncRNA MIR205HG in idiopathic pulmonary fibrosis and IL-33 regulation via Alu elements
Source: JCI Insight. 2025 Mar 10;10(5):e187172. doi: 10.1172/jci.insight.187172 (PMC11949018; doi:10.1172/jci.insight.187172)
Supplement: Supplemental data [file jciinsight-10-187172-s026.pdf]

Supplemental data

Involvement of lncRNA *MIR205HG* in idiopathic pulmonary fibrosis and IL-33 regulation via *Alu* elements

Tsuyoshi Takashima[1], Chao Zeng[2], Eitaro Murakami[3], Naoko Fujiwara[4], Masaharu Kohara[1], Hideki Nagata[5], Zhaozu Feng[1], Ayako Sugai[3], Yasue Harada[3], Rika Ichijo[6], Daisuke Okuzaki[7,8], Satoshi Nojima[1], Takahiro Matsui[1], Yasushi Shintani[5], Gota Kawai[6], Michiaki Hamada[2,9], Tetsuro Hirose[4,8], Kazuhiko Nakatani[3,8] and Eiichi Morii[1,8*]

1. Department of Pathology, Osaka University Graduate School of Medicine, Osaka, Japan
2. Faculty of Science and Engineering, Waseda University, Tokyo, Japan
3. Department of Regulatory Bioorganic Chemistry, SANKEN (the Institute of Scientific and Industrial Research)
4. Graduate School of Frontier Biosciences, Osaka University, Osaka, Japan
5. Department of General Thoracic Surgery, Osaka University Graduate School of Medicine, Osaka, Japan
6. Department of Life Science, Graduate School of Advanced Engineering, Chiba Institute of Technology, Chiba, Japan
7. Laboratory of Human Immunology (Single Cell Genomics), WPI Immunology Frontier Research Center, Osaka University, Osaka, Japan
8. Institute for Open and Transdisciplinary Research Initiatives, Osaka University, Osaka, Japan
9. AIST-Waseda University Computational Bio Big-Data Open Innovation Laboratory (CBBD-OIL), National Institute of Advanced Industrial Science and Technology, Tokyo, Japan

* Correspondence: Eiichi Morii, Department of Pathology, Osaka University Graduate School

of Medicine. 2-2 Yamada-Oka, Suita, Osaka 565-0871, Japan. Phone: +81-6-6879-3871;

E-mail: morii@molpath.med.osaka-u.ac.jp

Methods

RNA ISH staining

Probes targeting Hs-*MIR205HG* (Cat. #588711, Advanced Cell Diagnostics), Mm-*Mir205hg* (Cat. #1277451, Advanced Cell Diagnostics) and Hs-*PPIB* (Cat. #313901, Advanced Cell Diagnostics) were used. To confirm sample quality, all FFPE samples were verified for a *PPIB* signal (>3–5 dots/cell). FFPE sections were deparaffinised in xylene, and dehydrated in 100% ethanol. After air-drying, the sections were treated with RNAscope® Hydrogen Peroxide solution (Cat. #322335, Advanced Cell Diagnostics) for 10 min at room temperature (RT) and washed with distilled water. The sections were incubated in RNAscope® Target Retrieval reagent (Cat. #322000, Advanced Cell Diagnostics) at 95°C using a Decloaking Chamber NxGen (Cat. #DC2012, Biocare Medical, Pacheco, CA, USA) for 15 min, then washed with distilled water. The sections were treated with RNAscope® Protease Plus reagent (Cat. #322331, Advanced Cell Diagnostics) for 30 min at 40°C in a HybEZ II oven (Advanced Cell Diagnostics). *MIR205HG* or *PPIB* probes ere then applied for 2 h at 40°C and incubated with reagents AMP1 (30 min at 40°C), AMP2 (15 min at 40°C), AMP3 (30 min at 40°C), AMP4 (15 min at 40°C), AMP5 (30 min at RT), and AMP6 (15 min at RT). The reagents were applied in amounts sufficient to cover the tissue. Slides were rinsed with RNAscope® Wash Buffer (Cat. #310091, Advanced Cell Diagnostics) (2 × 2 min) between each AMP incubation. Hybridisation signals were detected by staining with DAB (Agilent Technologies) for 10 min at RT, followed by counterstaining with hematoxylin.

ISH combined with IHC staining

The ISH and FISH combined with IF staining conditions described above were used as a reference. FFPE tissue sections were subjected to ISH staining using the RNAscope® 2.5 HD Detection Reagents-BROWN Kit (Advanced Cell Diagnostics), in accordance with the manufacturer’s instructions. Hybridization signals were detected by DAB (Agilent

Technologies) staining for 10 min at RT. HRP activity was inactivated overnight with 10 mM sodium azide (Nacalai Tesque). The slides were then processed for IHC staining, by reacting with the indicated primary antibody for 1 h and then with the indicated secondary antibody for 1 h. Signals were detected using the Ventana DISCOVERY Purple Kit (Roche) and Ventana DISCOVERY Green Kit (Roche) staining, followed by counterstaining with hematoxylin.

Fluorescence in situ hybridization (FISH) combined with immunofluorescence (IF) staining and scoring

FFPE tissue sections were subjected to for FISH staining using the RNAscope® Multiplex Fluorescent Reagent Kit v2 (Cat. #323100, Advanced Cell Diagnostics) combined with IF staining. FISH staining was performed in accordance with manufacturer's instructions. Briefly, the sections were incubated in EnVision FLEX Target Retrieval Solution, High pH (50x) (Cat. #K8000, Agilent Technologies) at 110°C using a Decloaking Chamber NxGen (Biocare Medical) for 15 min. The sections were treated with RNAscope® Protease Plus reagent (Advanced Cell Diagnostics) for 30 min at 40°C in a HybEZ II oven (Advanced Cell Diagnostics). A probe targeting Hs-*MIR205HG* (Cat. #588711, Advanced Cell Diagnostics) was then applied for 2 h at 40°C and incubated with reagents AMP1 (30 min at 40°C), AMP2 (30 min at 40°C), AMP3 (15 min at 40°C), and horseradish peroxidase (HRP)-C1 (15 min at 40°C). The reagents were applied in amounts sufficient to cover the tissue. The slides were rinsed with RNAscope® Wash Buffer (Advanced Cell Diagnostics) (2 × 2 min) between each AMP incubation. Hybridization signals were detected using the Opal 4-Color (Cat. #NEL810000KT, Akoya Biosciences, Marlborough, MA, USA) or Opal 7-Color (Cat. #NEL811001KT, Akoya Biosciences) Manual IHC kit for 30 min at 40°C. HRP activity was inactivated overnight with 10 mM sodium azide (Cat. #31233-42, Nacalai Tesque). The slides were then subjected to IF staining, reacted with the indicated primary antibody for 1 h, and reacted with the indicated secondary antibody for 1 h. The antibodies were stripped by

VectaPlex™ Antibody Removal Kit (Cat. #VRK-1000, Vector Laboratories, Newark, CA USA). Details of the antibodies are described in Supplemental Table 1. DAPI (Cat. #FP1490, Akoya Biosciences) nuclear staining was performed for 10 min and the slide were then coverslipped using Fluoro-KEEPER Antifade Reagent, Non-Hardening Type without DAPI (Cat. #12593-64, Nacalai Tesque). Fluorescent images were obtained using an LSM880 and LSM980 Confocal Microscope (Carl Zeiss, Oberkochen, Germany) and analyzed using NIS-Elements v. 5.21 software (Nikon, Tokyo, Japan).

Double ISH staining

The FISH staining conditions described above were used as a reference. Probes targeting Hs-*MIR205HG*-C1 (Advanced Cell Diagnostics) and Hs-*IL33*-C2 (Cat. #400111-C2, Advanced Cell Diagnostics) were used. HRP activity was inactivated with HRP blocker (Cat. #323107, Advanced Cell Diagnostics) when transitioning from the C1 probe reaction to the C2 probe reaction. Fluorescent images were obtained using an LSM880 Confocal Microscope (Carl Zeiss).

Cell culture

Primary NHBE cells were obtained from LONZA (Cat. #CC-2540, LONZA, Walkersville, MD, USA). The cells were cultured in BEGM™ Bronchial Epithelial Cell Growth Medium BulletKit™ (Cat. #CC-3170, LONZA). BEAS-2B (Cat. #CRL-9609, ATCC, Manassas, VA, USA) and Lenti-X 293T cells (Cat. #632180, Takara) were cultured in DMEM/Ham's F-12 (Cat. #08460-95, Nacalai Tesque) supplemented with 10% fetal bovine serum (FBS) (Cat. #35-010-CV, Corning), penicillin (100 IU/mL, Cat. #022-18132, FUJIFILM Wako Pure Chemical, Osaka, Japan), and streptomycin (100 µg/mL, Cat. #32777-44, Nacalai Tesque). Cells were maintained at 37°C in a 5% CO_2 atmosphere.

Culture of human lung organoids

Alveolar organoids were passaged approximately every 2 weeks, and IPF patient-derived airway organoids were passaged approximately every week. For passages, organoids were dissected using trypsin, and the digestive enzymes were inactivated with DMEM/Ham's F-12 (Nacalai Tesque) supplemented with 10% FBS (Corning). At passages, cells were seeded at 5,000 cells per well.

Bleomycin stimulation in alveolar organoids

Alveolar organoids were seeded with the indicated number of cells and cultured for 9 days. The organoids were then treated with bleomycin (Cat. #4234400D5039, Nippon Kayaku, Tokyo, Japan) at concentrations of 10 μM and 100 μM for 3 days. Bleomycin treated cells were harvested at the indicated time points and samples were prepared for RNA extraction.

Bleomycin-induced pulmonary fibrosis mouse model

Eight-week-old mice (C57BL/6J strain, CLEA Japan, Shizuoka, Japan) were anesthetized with isoflurane and administered a single intratracheal dose of bleomycin (5 mg/kg, 60 μl) (Nippon Kayaku) on day 0. These mice were sacrificed after 21 days and fixed intratracheally with 10 % formalin neutral buffer solution (Cat. # 37152-51, Nacalai Tesque).

Public data analysis of bulk RNA-seq (Bleomycin-induced pulmonary fibrosis mouse model)

Bulk RNA-seq datasets were downloaded from the GEO datasets GSE173523 (1) and GSE217816 (2).

Lentivirus production

Lenti-X 293T cells (Takara) were plated in 10 cm dishes at 80% confluence the day before transfection. Lentiviral particles were generated using the TransIT-293 Transfection Reagent (Cat. #MIR2705, Takara) and the Lentiviral High Titer Packaging Mix (Cat. #6194, Takara), in accordance with the manufacturer's instructions. Supernatants were harvested 48 h after transfection. The collected lentiviral supernatants were passed through a 0.45 μm filter (Cat. #16555, Sartorius, Göttingen, Germany), concentrated 10-fold using Lenti-X™ Concentrator (Cat. #631231, Takara), and stored at −80°C until further use.

Preparation of cell blocks for NHBE cells and organoids

For organoids, Matrigel® was dissolved using Cell Recovery Solution (Cat. #354253, Corning). NHBE cells and organoids were fixed in 10% formaldehyde neutral buffer solution for 2 days. The NHBE cells and organoids were then washed with phosphate-buffered saline (PBS), centrifuged, and pre-embedded in agarose gel (Agarose L03 [TAKARA], Cat. #5003, Takara), before undergoing paraffin embedding with the same method used for tissue sample processing.

Establishment of human lung organoids

Briefly, normal lung or IPF patient tissues were sampled from a macroscopically peripheral area within 1–2 cm of the region with gross margins from the tumor. For IPF patients, tissue was sampled from fibrotic areas. The tissues were washed with HBSS (Cat. #14170112, Thermo Fisher Scientific, Waltham, MA, USA) and cut into small pieces. The fragments were digested with 1 mg/mL Collagenase/Dispase® (Cat. #11097113001, Roche), 10 μM Y-232632 (Cat. #036-24023, Abmole), and 0.1％ DNase I (Cat. #04716728001, Roche) at

37°C for 60 min. The cells were then treated with trypsin (Cat. #32777-44, Nacalai Tesque, Kyoto, Japan) for 30 min to dissociate them. Collected cells were washed with DMEM/Ham's F-12 (Nacalai Tesque) supplemented with 10% FBS (Corning, Corning, NY, USA) to inactivate the digestive enzymes.

Quantification of organoid number

Alveolar organoids were dissociated into single cells using trypsin (Nacalai Tesque) and filtered through a FALCON® strainer (Cat. #E352235, Corning). Cells were seeded at 5,000 cells per well (48-well plate, Greiner Bio-One). Organoid numbers were measured 12–14 days after passaging. Images of each well were captured using a BZ-X800 digital microscope (Keyence, Osaka, Japan). The sphere area was automatically calculated using the BZ-X800 Analyzer (Keyence). Colonies with areas greater than 2,500 mm^2 were used for area measurement.

Proliferation assay

NHBE cells were seeded at 5,000 cells per well in 96-well plate (Greiner Bio-One). Cell numbers were monitored at 0, 1, 2 and 4 days using the Cell Counting Kit-8 (Cat. #TN654, Dojindo Laboratories, Kumamoto, Japan), in accordance with manufacturer's instructions. The absorbance at 450 nm was measured using an SH-9000 Lab microplate reader (Hitachi, Tokyo, Japan) after 60 min of incubation.

RNA extraction and qRT-PCR analysis

Total RNA was isolated from primary cells and organoids using RNAiso Plus (Cat. #9109, Takara). A quantity of 100–500 ng of total RNA was converted to complementary DNA (cDNA) using the SuperScript III First-Strand Synthesis System (Cat. #18080051, Thermo

Fisher Scientific), in accordance with the manufacturer's instructions. Quantitative PCR assays were performed using TaqMan® Fast Advanced Master Mix (Cat. #4444965, Thermo Fisher Scientific) on the StepOnePlus System (Thermo Fisher Scientific). The primer sequences are listed in Supplemental Table 2. The *ACTB* housekeeping gene was used for normalization. For miRNA detection, 10 ng of RNA were reverse-transcribed using the TaqMan™ miRNA Reverse Transcription Kit (Cat. #4366596, Thermo Fisher Scientific). The *RNU6B* housekeeping gene was used for normalization. Forty cycles of amplification were performed for each sample. Cycle quantification values were calculated using StepOnePlus software ver. 2.3 (Thermo Fisher Scientific). Relative quantification was determined by the ΔΔCt method.

Western blotting

Cells were rinsed with ice-cold PBS and lysed in ice-cold lysis buffer [1% NP-40, (Cat. #25223-04, Nacalai Tesque), 20 mM Tris–HCl, 150 mM NaCl, and 1 mM EDTA] containing cOmplete™ Protease Inhibitor Cocktail (Cat. #04693159001, Roche) and PhosSTOP (Cat. #4906837001, Roche). The cells were sonicated for 10 s. Soluble fractions from the cell lysates were isolated by centrifugation at 13,000 rpm for 10 min in a microcentrifuge. Next, 2× SDS buffer (4% SDS, 125 mM Tris–HCl pH 6.8, 20% glycerol, 0.01% bromophenol blue, and 10% 2-mercaptoethanol) was added to the cell lysates and boiled for 5 min at 95°C. Proteins were analyzed by SDS-polyacrylamide gel electrophoresis (ATTO, Tokyo, Japan). Proteins were semi-dry transferred onto polyvinylidene fluoride membranes. The membranes were incubated with the indicated primary antibody for 1 h, followed by and then with the indicated secondary antibody for 1 h. Details of the antibodies are described in Supplemental Table 1. The HRP signals were visualized with Chemi-Lumi One Super reagents (Cat. #02230-30, Nacalai Tesque). Images were obtained with an imaging system (ChemiDoc Touch; Bio-Rad, Hercules, CA, USA). ImageJ software (National Institutes of Health, Bethesda, MD, USA) was used to quantify the results.

RT-PCR

The steps from RNA extraction to cDNA synthesis were performed with reference to the qRT-PCR protocol described above. cDNA samples were amplified using KOD FX Neo (Cat. #KFX-201, TOYOBO). The primer sequences for *MIR205HG* (Hokkaido System Sciences, Hokkaido, Japan) were as follows:

(Forward) 5′-CTGAGTCCCTCTTGCTCACC-3′

(Reverse) 5′-GCTGCTACAGGCAGAGACAA-3′

PCR was performed on the T100™ Thermal Cycler (Bio-Rad). PCR amplification consisted of an initial denaturation for 2 min at 94°C, followed by 35 cycles of denaturation (10 s, 98°C), annealing (30 s, 74°C), and extension (30 s, 72°C). PCR products were separated by 1.0% agarose gel electrophoresis and visualized using the ChemiDoc Touch (Bio-Rad).

Sequence similarity analysis

Sequence similarity between *MIR205HG* and basal cell enriched gene (1465 genes, Figure 1D and 7D, GSE136831 (3)) was evaluated with BLASTn (4) using the following parameters: -task dc-megablast-evalue 0.001. We quantified the sequence similarity between *MIR205HG* and other genes by selecting the hit with the lowest E value. A lower E value indicates a higher degree of local similarity between the two genes. Additionally, we evaluated *MIR205HG*'s preference within the given gene set by analyzing the cumulative distribution function (CDF) of sequence similarity, where a higher CDF value reflects a stronger preference of *MIR205HG* for the corresponding gene.

Analysis of miRDB and TargetMiner dataset

miRNA target genes were analyzed using two publicly available algorithms: miRDB (https://mirdb.org/mirdb/index.html) (5) and TargetMiner (www.isical.ac.in/~bioinfo_miu) (6). Target genes were extracted from each database and merged with the DEGs of *MIR205HG*-OE alveolar organoids.

Analysis of 3D Genome Browser dataset

The distance between the genomic *MIR205HG* and *IL33* was analyzed using Hi-C public data (http://3dgenome.fsm.northwestern.edu/) (7).

Analysis of functional RNA structural element in *MIR205HG*

We used scanFold (8) with default parameters to perform a window (120 nt) scan on the *MIR205HG* RNA sequence and calculated the MFE Z-score in the shuffled sequences. A lower Z-score indicates a more stable secondary structure for the corresponding subsequence in that window. After extraction of extracting the window corresponding to the local minimum Z-score, we focused on the subsequence (hg38, chr1:209428995-209429115) overlapping with the *AluJb* repeat in *MIR205HG* and predicted its optimal secondary structure using RNAfold (9) (with default parameters), followed by visualization with forna (10).

Target RNA

The internal loop (IL) and hairpin loop (HL) motifs, which are the binding sites of the molecule, were extracted from the secondary structure of the *AluJb* sequence that was assumed in the interaction mechanism with *IL33* according to *MIR205HG* sequence data. Four RNAs (IL001-004) were used for the IL motif, which was incorporated into 5′-GUAC XXX CAUG UUU CAUG XXX GUAC-3′; three RNAs (HL001-003) were used for the HL motif, which was incorporated into 5′-GUAC CAUG XXX CAUG GUAC-3′. Each of the

seven motif sequences is summarized in Supplemental Table 6.

SPR (screening)

5′-Biotinylated RNAs IL001-004 and HL001-003 (GeneDesign, Osaka, Japan) were immobilized on the streptavidin-coated surface of a Series S Sensor Chip SA (Cytiva, Tokyo, Japan). All immobilization reactions were performed in HBS-EP^{+} buffer [0.01 mM HEPES, pH 7.4, 0.15 mM NaCl, 3 mM EDTA, and 0.05% (v/v) surfactant P20] using a Biacore 8K+ instrument (Cytiva) at 25ºC. The average amounts of immobilised IL001-004 and HL001-003 on the chip surface were 3496, 3775, 3231, 3608, 2920, 3068, and 3024 response units (RU), respectively. Sensorgrams were obtained at ligand concentrations of 50 μM (flow rate 60 μM/min, contact time 15 s, and dissociation time 15 s). All sensorgrams were corrected by reference subtraction of blank flow cell response and buffer injection response. Hit small molecules were evaluated by scoring the sensorgrams. Briefly, this score was calculated by considering the rate of change upon association and dissociation.

SPR (single cycle kinetics)

5′-Biotinylated RNAs IL001, HL003, and IL004 were immobilised on the streptavidin-coated surface of a Series S Sensor Chip SA (Cytiva). All immobilization reactions were performed in HBS-EP+buffer using a T200 instrument (Cytiva) at 25 ºC. The approximate amounts of immobilised IL001, HL003 and IL004 on the chip surface were 557, 2765, and 303 RU, respectively. Sensorgrams were obtained at ligand concentrations of 3.13, 6.25, 12.5, 25, and 50 μM in single-cycle mode (flow rate 60 μM/min, contact time 30 s, and dissociation time 120 s). All sensorgrams were corrected by reference subtraction of blank flow cell response and buffer injection response.

NMR

RNA samples (Hokkaido System Sciences) were dissolved in 20 mM sodium phosphate buffer (pH 6.4) with 50 mM sodium chloride and 5% D_2O. For NMR experiments, the RNA concentration was 0.38 mM for *MIR205HG*_HL003. All NMR spectra were measured using the AvanceNeo 600 spectrometer (Bruker Biospin, Ettlingen, Germany) at 288 K. For the measurements of imino proton spectra, the water signal was suppressed by a jump-and-return pulse (11). NMR spectra were processed with TopSpin (Bruker Biospin). Titrations of RNAs were performed by adding a small amount of 10 mM DQzG in DMSO-d_6.

Supplemental Figure

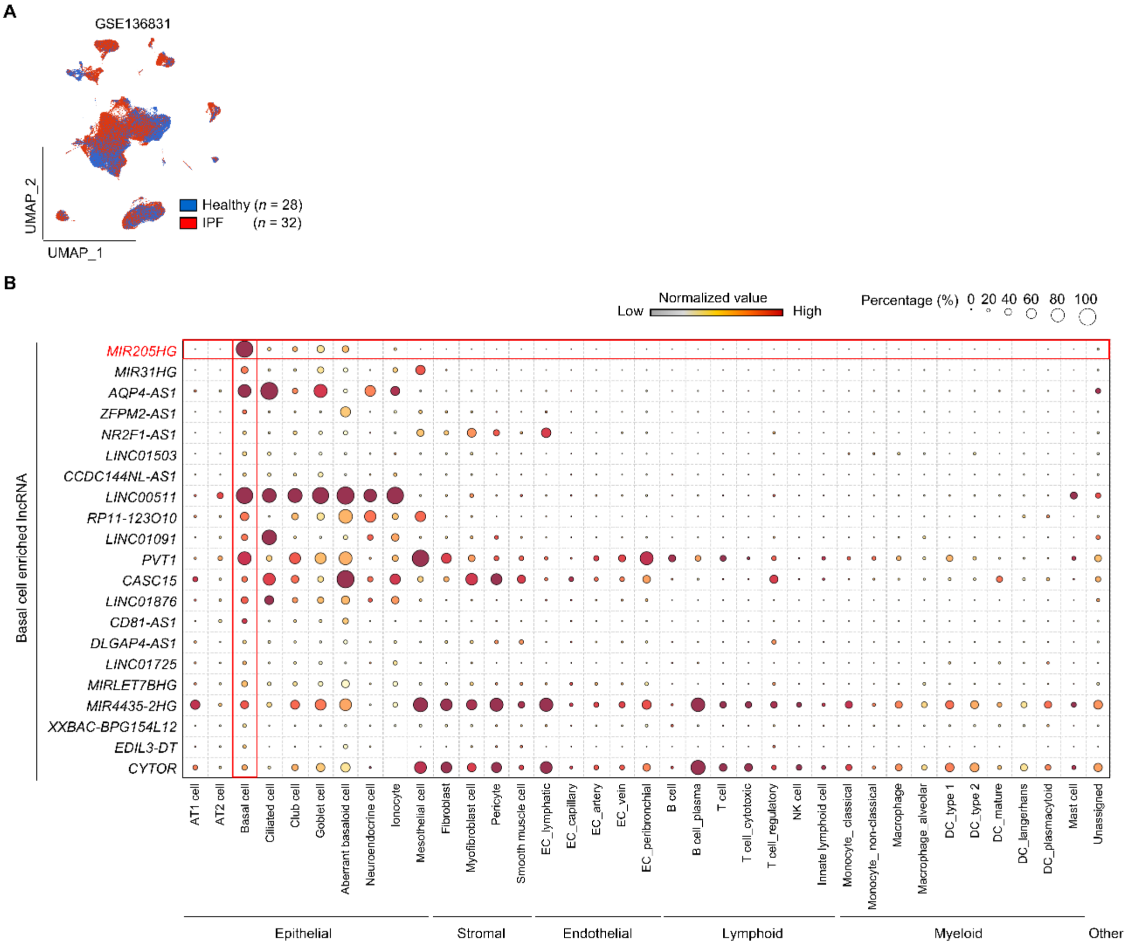

Supplemental Figure 1

Analysis of public scRNA-seq data (related to Figure 1)

(A) UMAP visualization of disease state in public scRNA-seq data (GSE136831 (3)) of healthy (*n* = 28) and IPF (*n* = 32) lungs. BBrowser software was used for disease state annotation and visualization.

(B) Bubble heatmap showing the gene score of a basal cell enriched lncRNAs (Figure1D, right) in the total cell subset identified in Figure 1B (GSE136831).

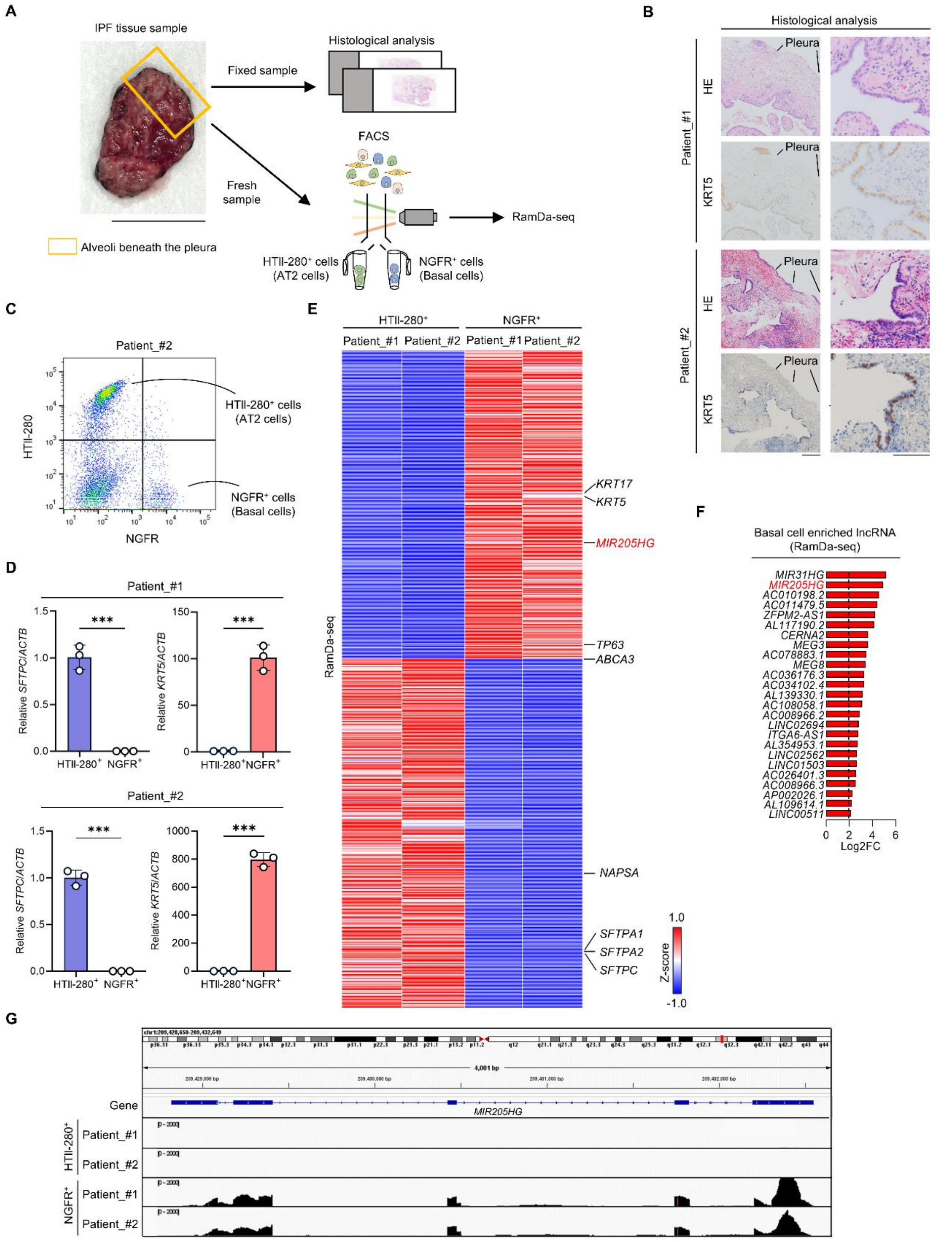

Supplemental Figure 2

Analysis of RNA-seq sorted AT2 cells and basal cells in IPF patients (related to Figure 1)

(A) Overview of experiments to identify lncRNAs highly expressed in basal cells in IPF patients.

(B) Representative images of HE and KRT5 IHC staining. Scale bar: 100 μm.

(C) Representative gating image for sorting HTII-280$^+$ cells (AT2 cells) and NGFR$^+$ cells (basal cells).

(D) qRT-PCR showing *SFTPC* and *KRT5* expression in HTII-280$^+$ cells (AT2 cells) and NGFR$^+$ cells (basal cells). Data represent mean ± SD. ***$P < 0.001$; P values were determined by two-tailed Student's *t*-test.

(E) Heatmap showing DEGs in sorted HTII-280$^+$ cells (AT2 cells) and NGFR$^+$ cells (basal cells) from two IPF patients. Genes were extracted with log2FC > 2 and FPKM > 5 as threshold values.

(F) Bar graph of lncRNAs with differential expression in NGFR$^+$ cells (basal cells).

(G) IGV plot showing *MIR205HG* transcripts in HTII-280$^+$ cells (AT2 cells) and NGFR$^+$ cells (basal cells).

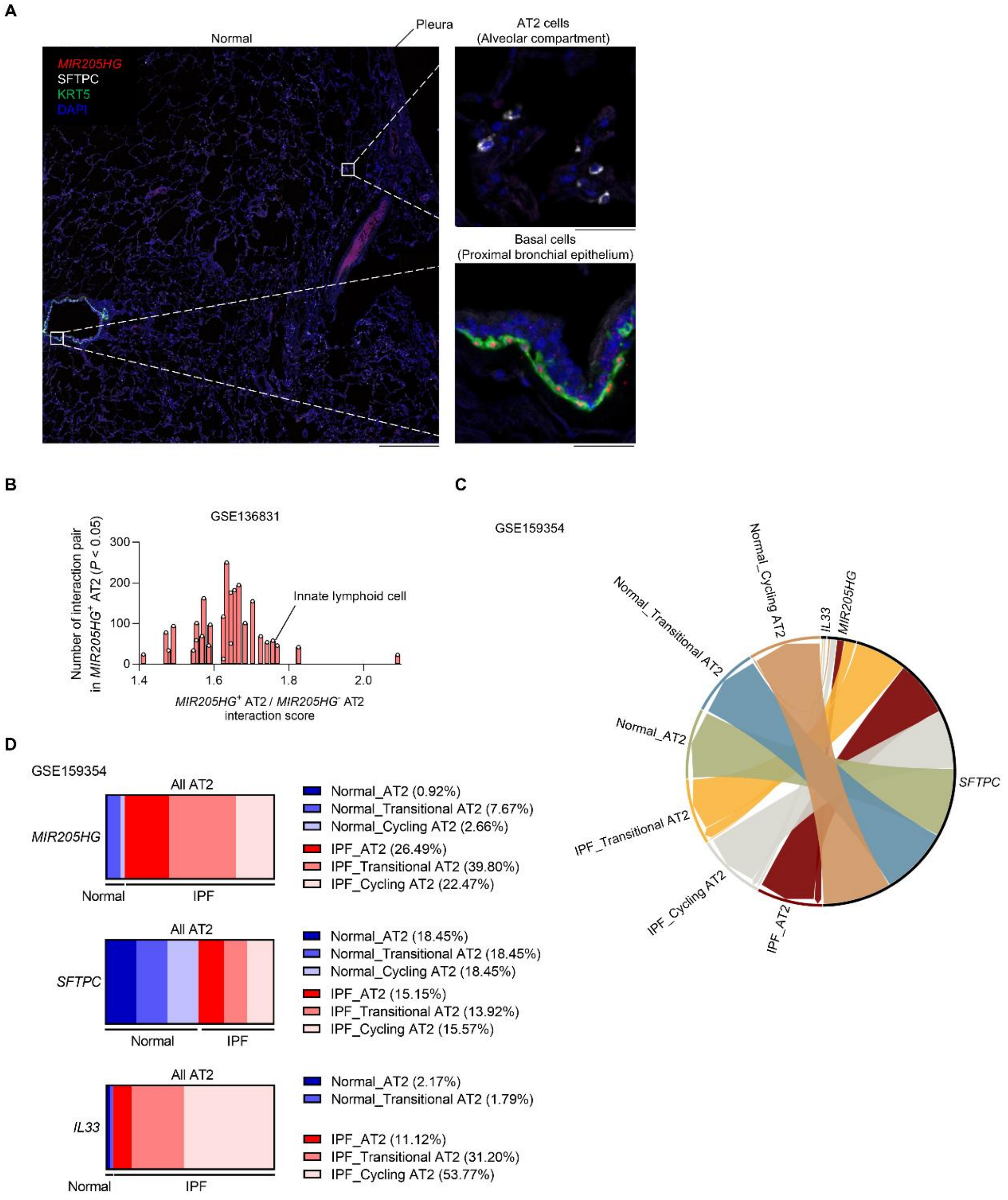

Supplemental Figure 3

Analysis of *MIR205HG*$^+$ AT2 cells using public scRNA-seq data (related to Figure 3 and 4)

(A) Representative confocal images of *MIR205HG* ISH (red), SFTPC IHC (white) and KRT5 IHC (green) staining in normal lung ($n = 6$). Scale bars: 1 mm (left) and 100 μm (right).

(B) Bar graph of cell–cell communication scores in the *MIR205HG*$^{+}$ AT2 cells versus *MIR205HG*$^{-}$ AT2 cells. Scores indicate values used in Figure 4B (GSE136831). *P* values were determined by the permutation test.

(C) Circos plot showing the relationship between AT2 cells of normal and IPF lungs and *IL33*, *MIR205HG*, and *SFTPC* genes using public scRNA-seq data (GSE159354 (12)). The size of each element indicates the ratio of each gene expression.

(D) Proportion of AT2 cells of normal and IPF lungs in *MIR205HG*, *SFTPC*, and *IL33* genes using public scRNA-seq data (GSE159354).

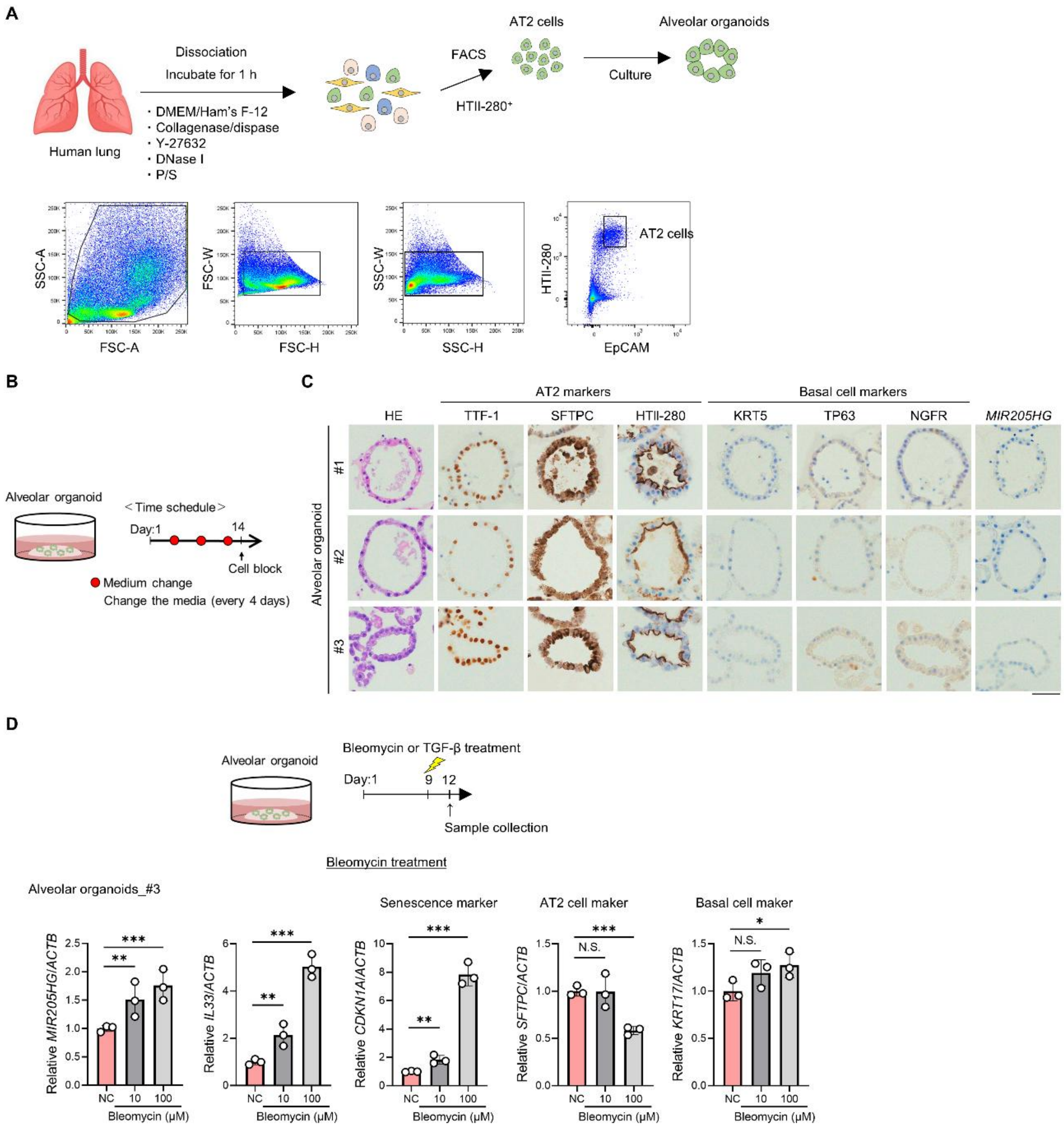

Supplemental Figure 4

Establishment of alveolar organoids from human normal lung (related to Figure 5)

(A) Schematic of experimental design for the isolation of AT2 cells from human lung(upper). Representative gating strategy for sorting HTII-280⁺EPCAM⁺ human AT2 cells (lower).

(B) Culture schedule for standard alveolar organoid cultures.

(C) Representative images of HE, IHC (TTF-1, SFTPC, HTII-280, KRT5, TP63, and

NGFR) and ISH (*MIR205HG*) staining in alveolar organoids. Scale bar: 50 µm.

(D) qRT-PCR showing expression of *MIR205HG*, *IL33*, *CDKN1A*, *SFTPC* and *KRT17* in 10 µM and 100 µM bleomycin treatment using alveolar organoids (#３). Data represent mean ± SD. N.S., not significant, **P* < 0.05, ***P* < 0.01, ****P* < 0.001; *P* values were determined by two-tailed Student's t-test.

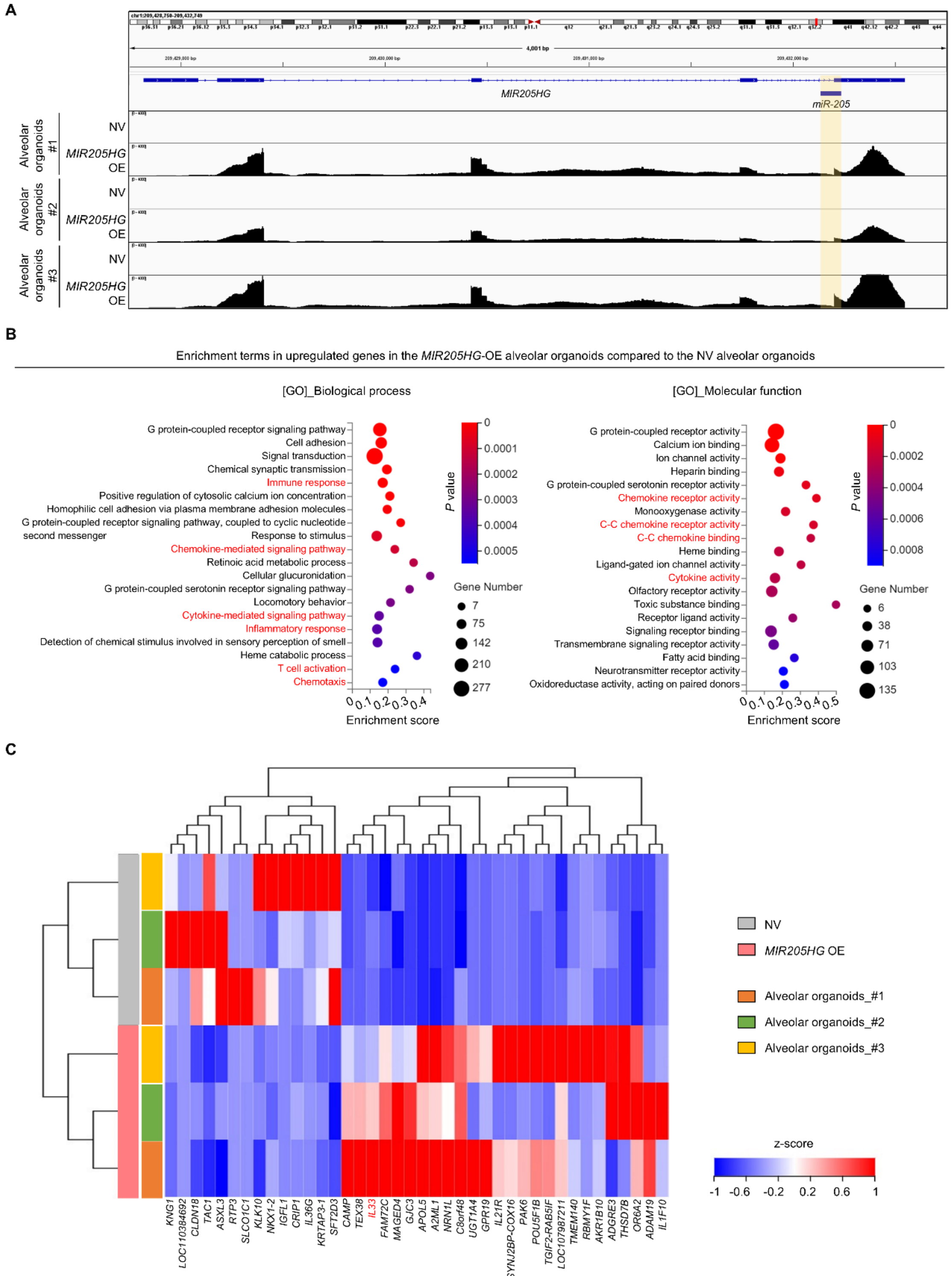

Supplemental Figure 5

Bulk RNA-seq analysis in *MIR205HG*-OE alveolar organoids (related to Figure 5)

(A) IGV plot showing *MIR205HG* transcripts in NV and *MIR205HG*-OE alveolar organoids. The *miR-205* was highlighted as a yellow background.

(B) Bubble plot showing enriched processes in upregulated genes in *MIR205HG*-OE alveolar organoids. Red indicates enriched processes related to inflammation.

(C) Heatmap showing top 40 genes with DEGs in *MIR205HG*-OE alveolar organoids compared with NV alveolar organoids. *IL33* gene is indicated in red among the upregulation genes in *MIR205HG*-OE alveolar organoids.

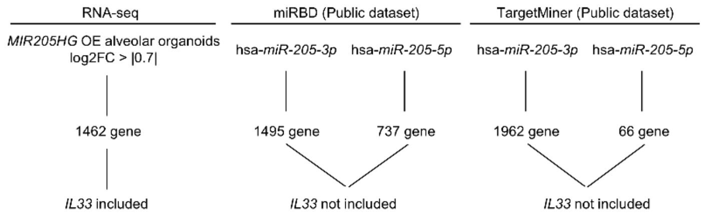

Supplemental Figure 6

Analysis of DEGs in *MIR205HG*-OE alveolar organoids and the target genes of *miR-205* with miRDB and TargetMiner public datasets (related to Figure 6)

Number of DEGs in *MIR205HG*-OE alveolar organoids (left) and target genes of *miR-205* with miRDB (5) (middle) and TargetMiner (6) (right) datasets. The results indicate whether the *IL33* gene is included in each condition.

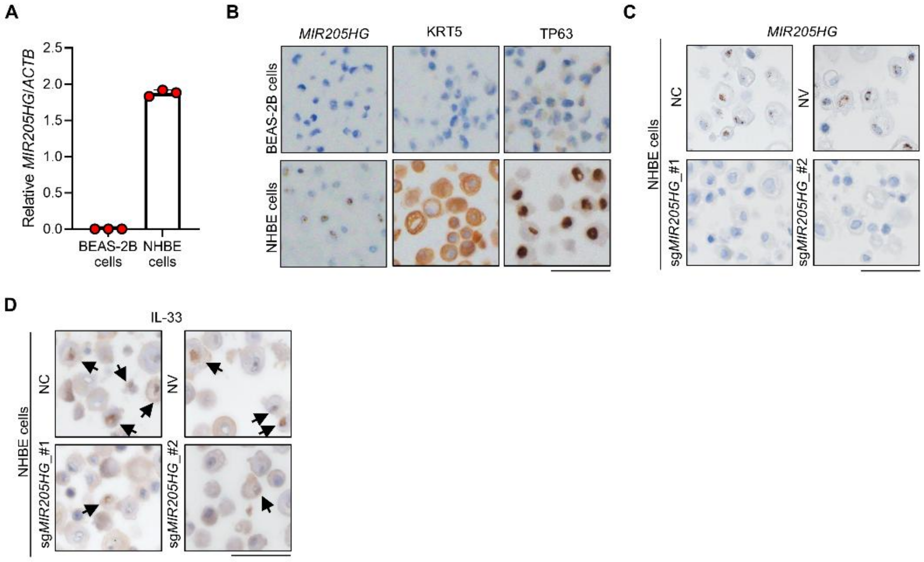

Supplemental Figure 7

Effect of *MIR205HG* knockdown in NHBE cells (related to Figure 7)

(A) qRT-PCR showing *MIR205HG* expression in BEAS-2B and NHBE cells. Data represent mean ± SD.

(B) Representative images of IHC (KRT5 and TP63) and ISH staining (*MIR205HG*) in BEAS-2B and NHBE cells. Scale bar: 50 µm.

(C) Representative images of *MIR205HG* ISH staining in *MIR205HG*-KD NHBE cells. Scale bar: 50 µm.

(D) Representative images of IL-33 IHC staining in *MIR205HG*-KD NHBE cells. Scale bar: 50 µm.

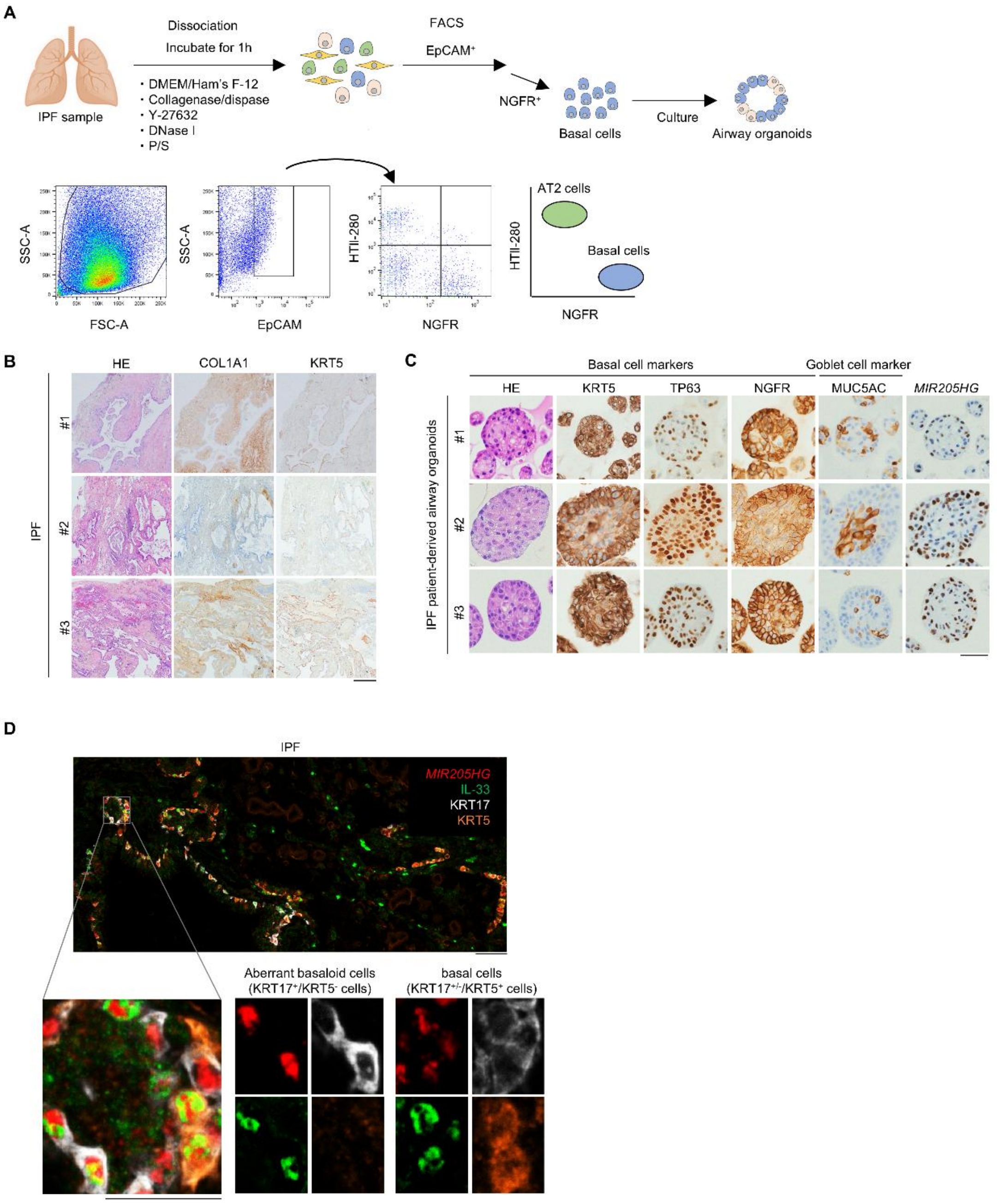

Supplemental Figure 8

Establishment of IPF patient-derived airway organoids and identification of *MIR205HG* positive cells in IPF patients (related to Figure 7)

(A) Schematic experimental design for isolation of basal cells from IPF patient samples (upper). Representative gating strategy for sorting EPCAM$^+$, NGFR$^+$, and HTII-280$^-$

human basal cells (lower).

(B) Representative images of HE and IHC staining (COL1A1 and KRT5) in IPF patient samples. Scale bar: 500 μm.

(C) Representative images of HE, KRT5, TP63, NGFR, MUC5AC, and *MIR205HG* staining in IPF patient-derived airway organoids. Scale bar: 50 μm.

(D) Representative confocal images of *MIR205HG* ISH (red), IL-33 IHC (green), KRT17 IHC (white) and KRT5 IHC (orange) staining in IPF patients (n = 3). Scale bars: 50 μm (upper) and 25 μm (lower).

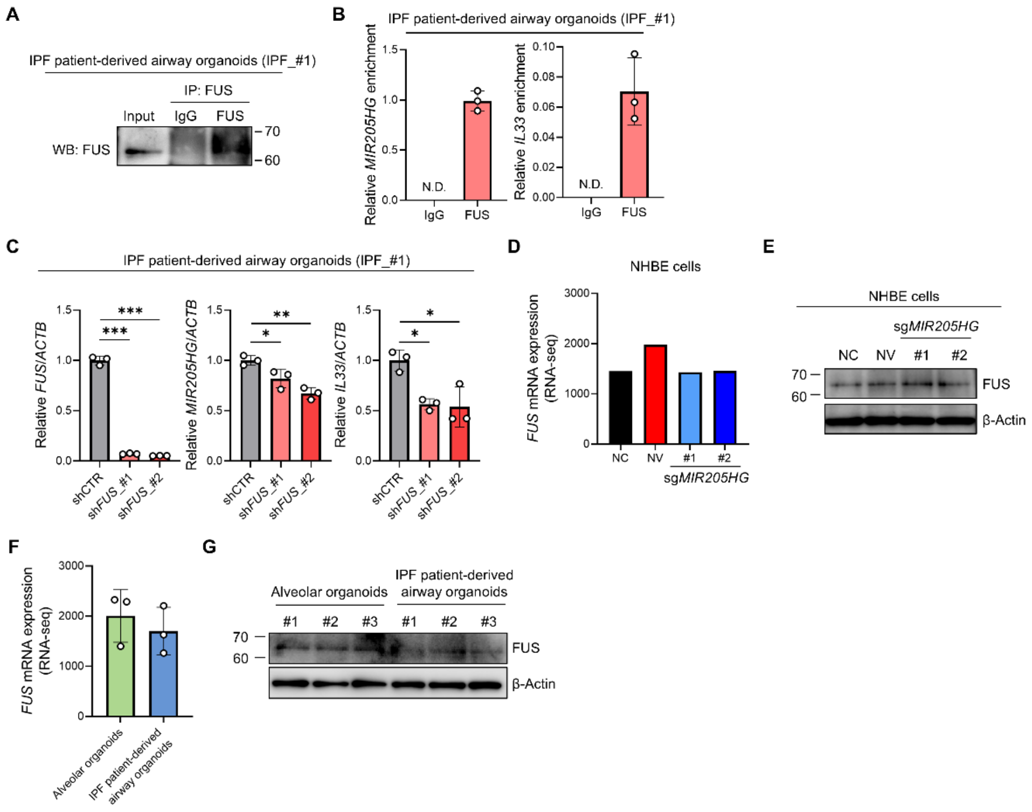

Supplemental Figure 9

FUS binding of *MIR205HG* and *IL33* mRNA in IPF patient-derived airway organoids, and FUS expression in *MIR205HG*-KD NHBE cells, alveolar organoids, and IPF patient-derived airway organoids (related to Figure 8 and 9)

(A) Western blot showing FUS protein expression in FUS IP using IPF patient-derived airway organoids.

(B) qRT-PCR showing *MIR205HG* and *IL33* in FUS RIP using IPF patient-derived airway organoids.

(C) qRT-PCR showing *FUS*, *MIR205HG,* and *IL33* expression of *FUS*-KD NHBE cells using shRNA. Data represent mean ± SD. $*P < 0.05$, $**P < 0.01$, $***P < 0.001$; P values were determined by ANOVA with Holm–Sidak's post-hoc test.

(D) RNA-seq showing *FUS* mRNA expression in NV and *MIR205HG*-KD NHBE cells.

(E) Western blot showing FUS protein expression in NV and *MIR205HG*-KD NHBE cells.

(F) qRT-PCR showing *FUS* mRNA expression in alveolar organoids and IPF patient-derived airway organoids.

(G) Western blot showing FUS protein expression in alveolar organoids and IPF patient-derived airway organoids.

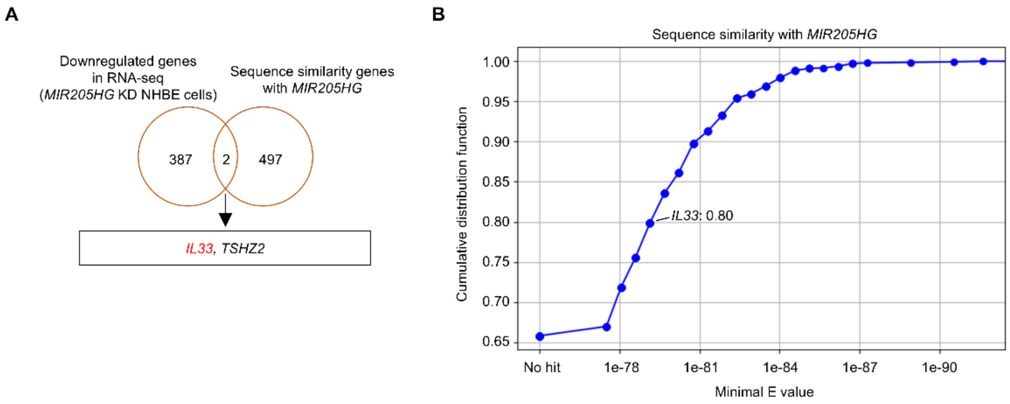

Supplemental Figure 10

Search for genes with sequence similarity to *MIR205HG* in basal cell enriched genes (related to Figure 10)

(A) Venn diagram showing downregulated genes in *MIR205HG*-KD NHBE cells (Figure 4E) and genes with sequence similarity to *MIR205HG*.

(B) Cumulative distribution function (CDF) of sequence similarity between the *MIR205HG* gene and other genes enriched in basal cells (1465 genes) in Figure1D and Figure 7D. Sequence similarity was assessed using the E value from BLASTn alignment, where a lower E value indicates a higher similarity of subsequences between two genes. The CDF value for the sequence similarity between the *MIR205HG* and *IL33* genes is 0.8, demonstrating that the *IL33* gene is among the top 20% of all enriched genes in terms of sequence similarity to the *MIR205HG* gene.

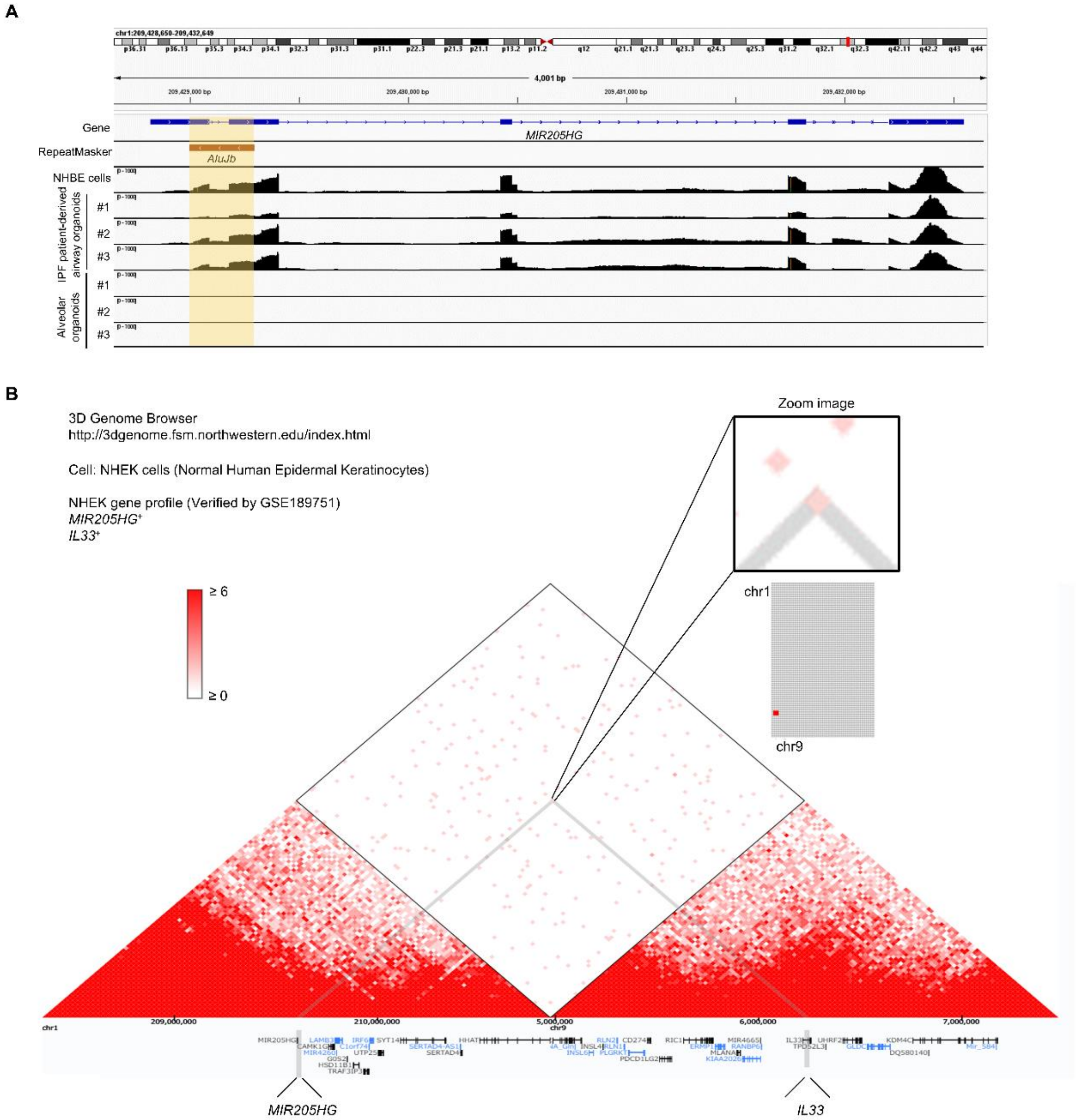

Supplemental Figure 11

Analysis of regulatory mechanism of *IL33* expression by *MIR205HG* (related to Figure 10)

(A) IGV plot showing *MIR205HG* transcripts. The *AluJb* element was highlighted as a yellow background. *AluJb* element transcripts were identified in NHBE cells and IPF patient-derived airway organoids, but not in alveolar organoids.

(B) Analysis of public Hi-C data using the 3D Genome Browser. Heatmap showing the

distance between *MIR205HG* (chr1) and *IL33* (chr9) in NHEK cells expressing *MIR205HG* and *IL33*.

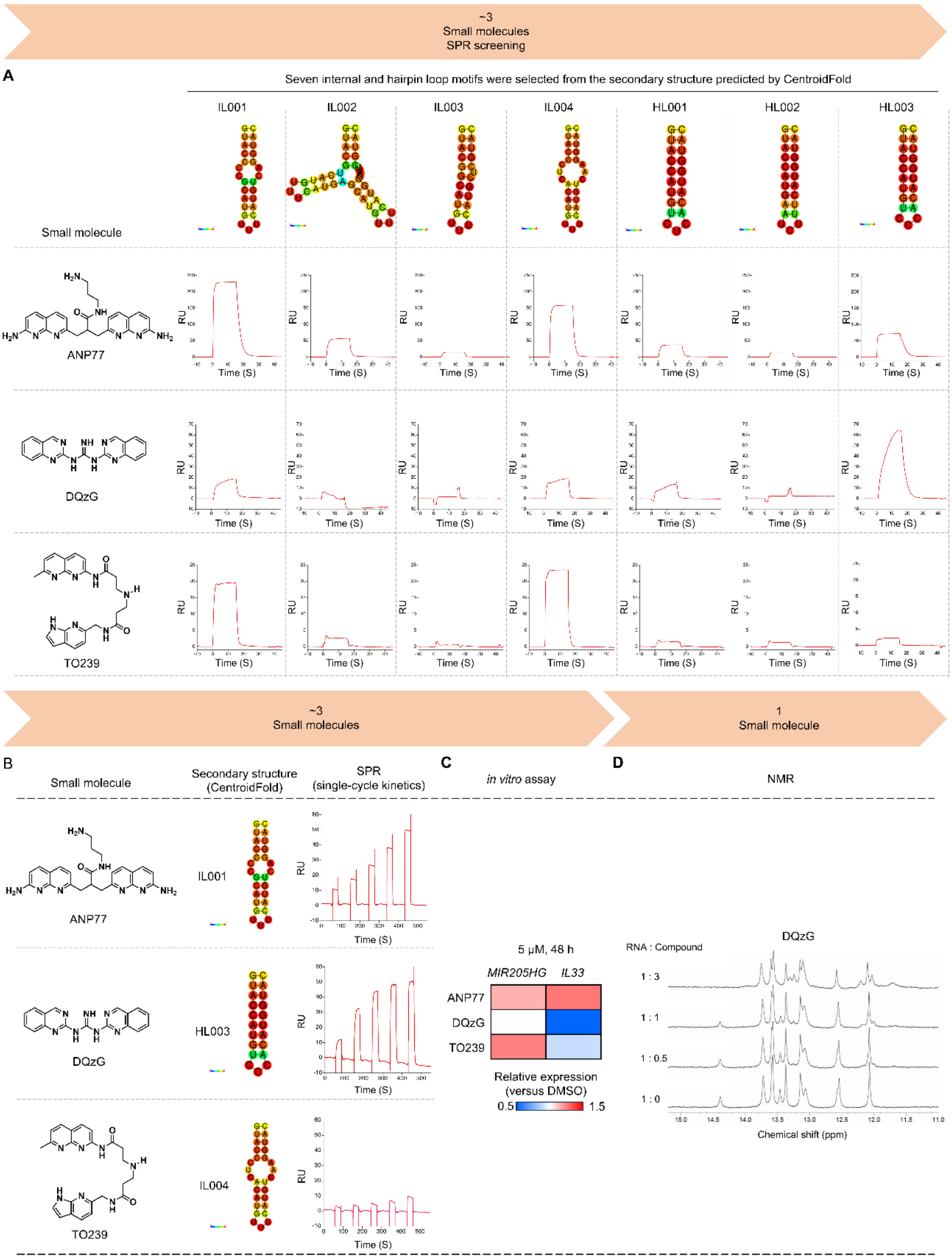

Supplemental Figure 12

Identification of small molecules binding to *AluJb* element of *MIR205HG* (related to Figure 11 and 12)

(A) Seven SPR-measurable internal and hairpin loop motifs were selected from the secondary structural *AluJb* element of *MIR205HG* by CentroidFold. Structures of three small molecules (ANP77, DQzG, and TO239) binding to *MIR205HG* predicted secondary structure containing the *AluJb* element. Sensorgrams for the binding to seven *MIR205HG* motif RNAs are shown for each small molecule.

(B) Small molecules identified the affinity to *MIR205HG* motif RNA showing an association and dissociation phase. Sensorgrams for the binding to each *MIR205HG* motif RNA are shown for ANP77 (*MIR205HG*_IL001), DQzG (*MIR205HG*_HL003), and TO239 (*MIR205HG*_IL004).

(C) Heatmap showing *MIR205HG* and *IL33* expression in NHBE cells. Expression levels after treatment with each small molecule treatment at 5 μM for 48 h, relative to DMSO (control), were determined by qRT-PCR.

(D) NMR analysis of DQzG binding to *MIR205HG*_HL003. Titration of RNA with DQzG at molar ratios of 1:0 to 1:3.

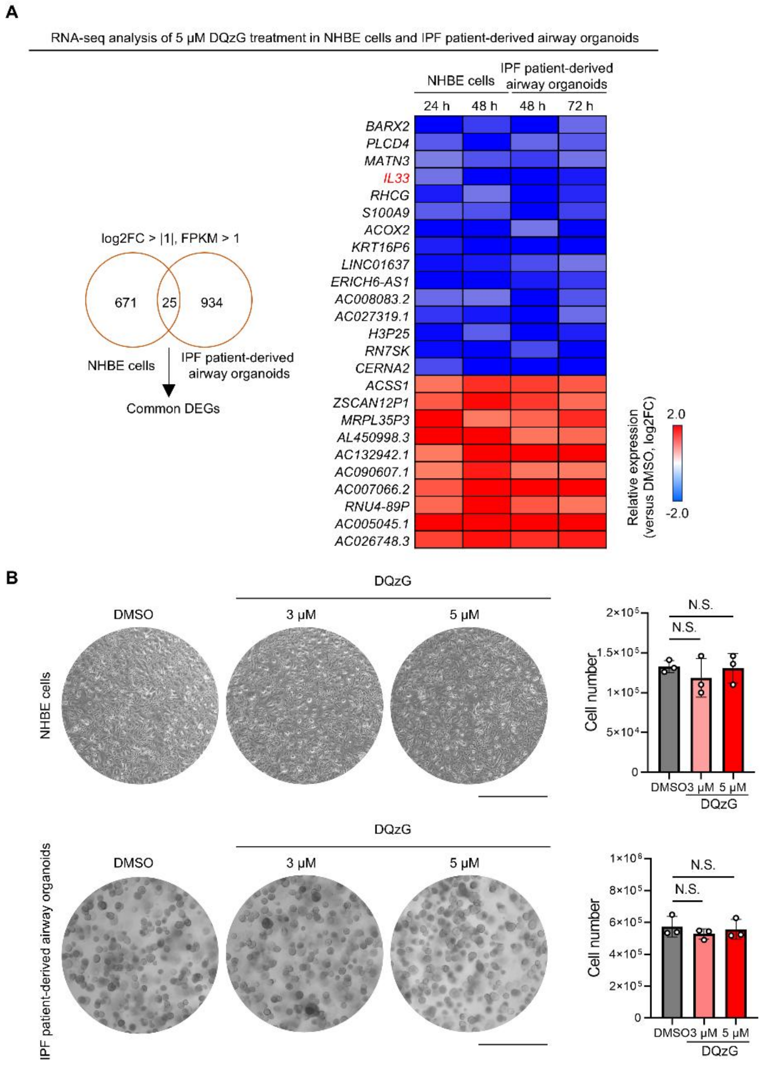

Supplemental Figure 13

Analysis of RNA-seq and cell numbers for DQzG treatment (related to Figure 11 and 12)

(A) Heatmap showing common DEGs in NHBE cells (24 h and 48 h) and IPF patient-derived airway organoids (48 h and 72 h) after 5 μM DQzG treatment. The common DEGs were extracted with log2FC > |1| and FPKM > 1 as threshold values.

(B) Cell numbers after 3 μM and 5 μM DQzG treatment in NHBE cells (after 48 h) and IPF patient-derived airway organoids (after 72 h). Scale bar: 1 mm. Data represent mean ± SD. N.S., not significant; *P* values were determined by 1-way ANOVA with Holm–Sidak's post-hoc test.

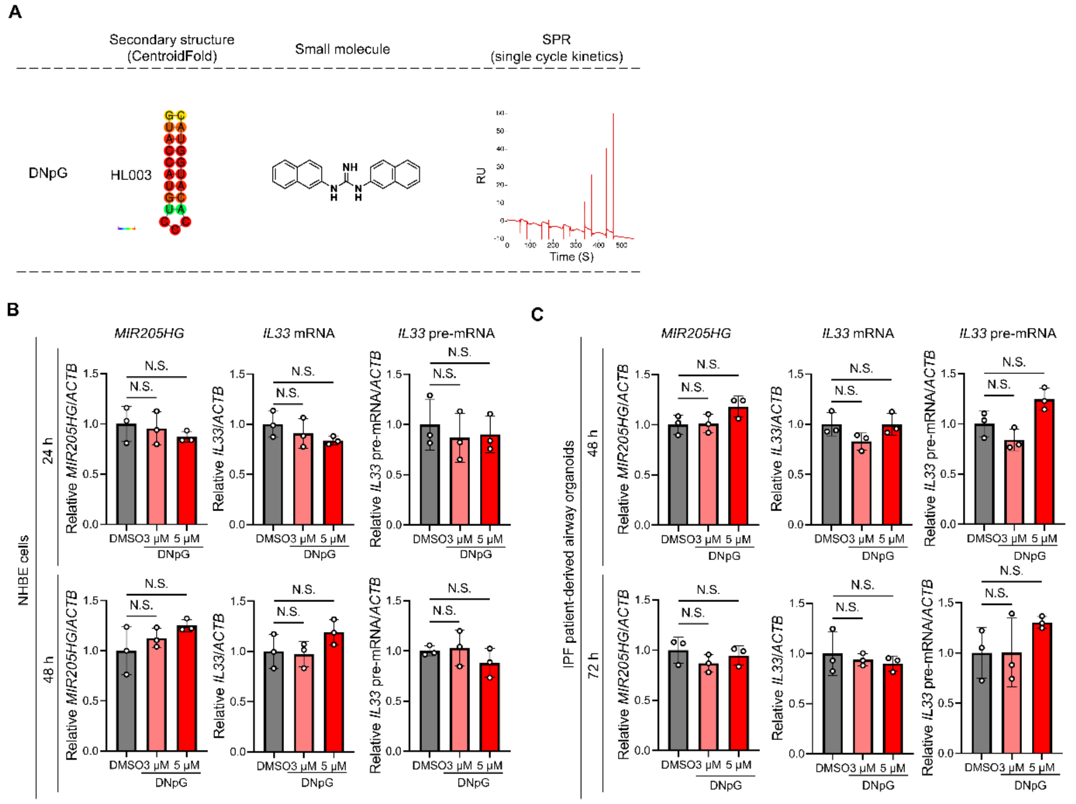

Supplemental Figure 14

Treatment of DNpG, a small molecule that did not bind to *AluJb* element recognized by DQzG (related to Figure 11 and 12)

(A) Structure of the small molecule DNpG. Sensorgrams of DNpG to *MIR205HG* motif RNA (*MIR205HG*_HL003) are shown (single-cycle kinetics).

(B) qRT-PCR showing *MIR205HG*, *IL33* mRNA, and *IL33* pre-mRNA expression in NHBE cells (24 h and 48 h) and IPF patient-derived airway organoids (48 h and 72 h) after 3 μM and 5 μM DNpG treatment. Data represent mean ± SD. N.S., not significant; *P* values were determined by 1-way ANOVA with Holm–Sidak's post-hoc test.

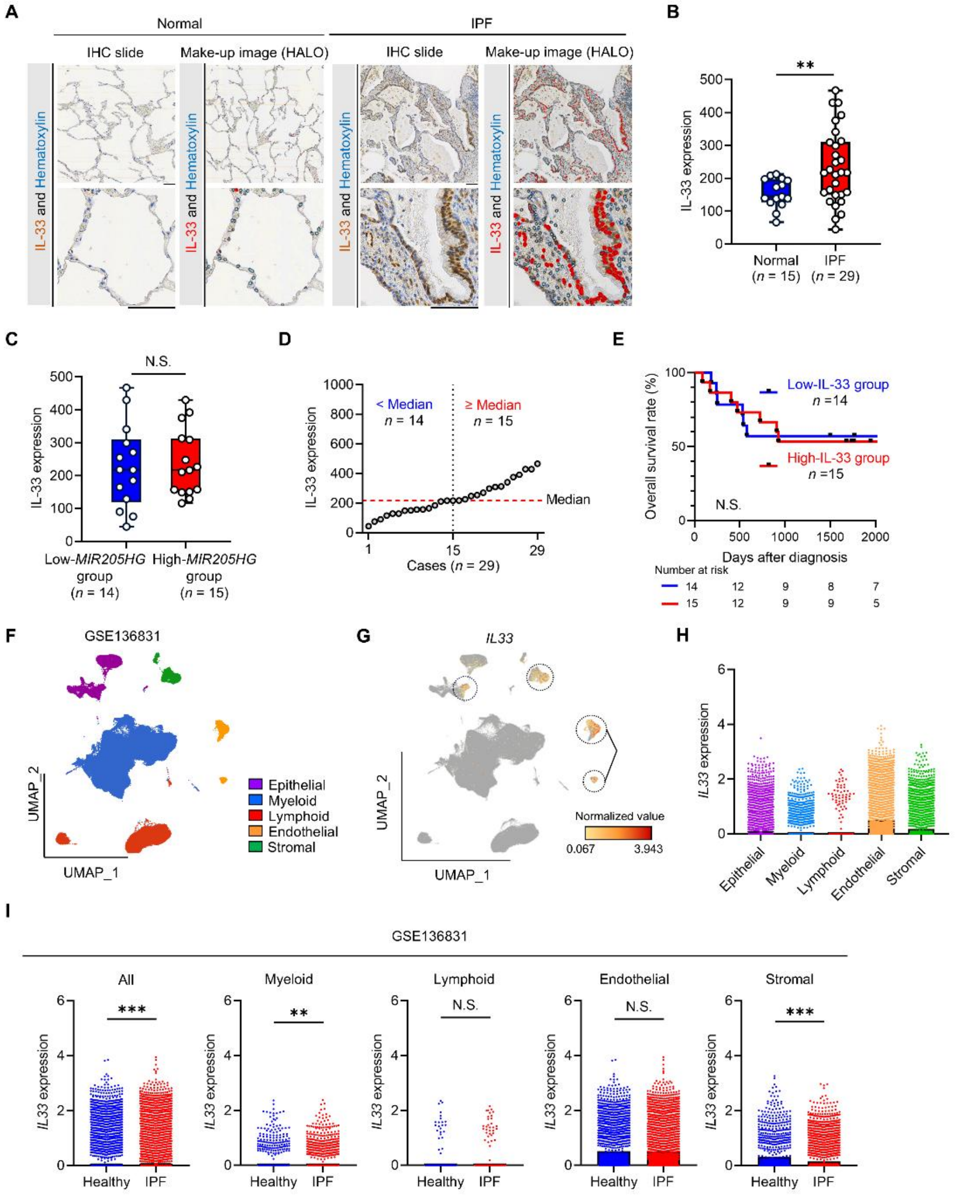

Supplemental Figure 15

Clinical significance of IL-33 expression in IPF patients (related to Figure 13)

(A) Representative image of IL-33 IHC staining in normal lungs (*n* = 15) and IPF patients (*n* = 29, cohort used in Figure 1G). IL-33 IHC staining image (left). Make-up image after HALO software analysis (right). Scale bar: 100 μm.

(B) Plot of IL-33 expression in normal lungs (*n* = 15) and IPF patients (*n* = 29). ***$P < 0.001$; *P* values were determined by 2-tailed Welch's *t*-test.

(C) Plot of IL-33 expression divided into high-*MIR205HG* group ($n = 15$) and low-*MIR205HG* group ($n = 14$). The two groups were based on expression of *MIR205HG* ISH used in Figure 1I. ****P* < 0.001; *P* values were determined by 2-tailed Mann–Whitney *U*-test.

(D) Plots of IL-33 expression in IPF patients ($n = 29$). The median was used as the cut-off value.

(E) Kaplan–Meier curves for overall survival rate (%) in IPF patients ($n = 29$) divided into high-IL-33 group ($n = 15$) and low-IL-33 group ($n = 14$). HR, 1.28; 95% CI, 0.45–3.63; N.S., not significant; *P* values were determined by log-rank test.

(F) UMAP visualization of cell cluster.

(G) UMAP visualization of *IL33* expression. Epithelial, stromal, and endothelial clusters with localized *IL33* expression are highlighted with dotted lines.

(H) Plot of *IL33* expression in all cell cluster.

(I) Plot of *IL33* expression for healthy lungs ($n = 28$) and IPF patients ($n = 32$) in all, myeloid, lymphoid, endothelial, and stromal cell clusters. The individual dots indicate the *IL33* expression in each cell cluster. ****P* < 0.001; *P* values were determined by 2-tailed Mann–Whitney *U*-test.

(F)–**(I)** Public scRNA-seq data (GSE136831) was used for analysis.

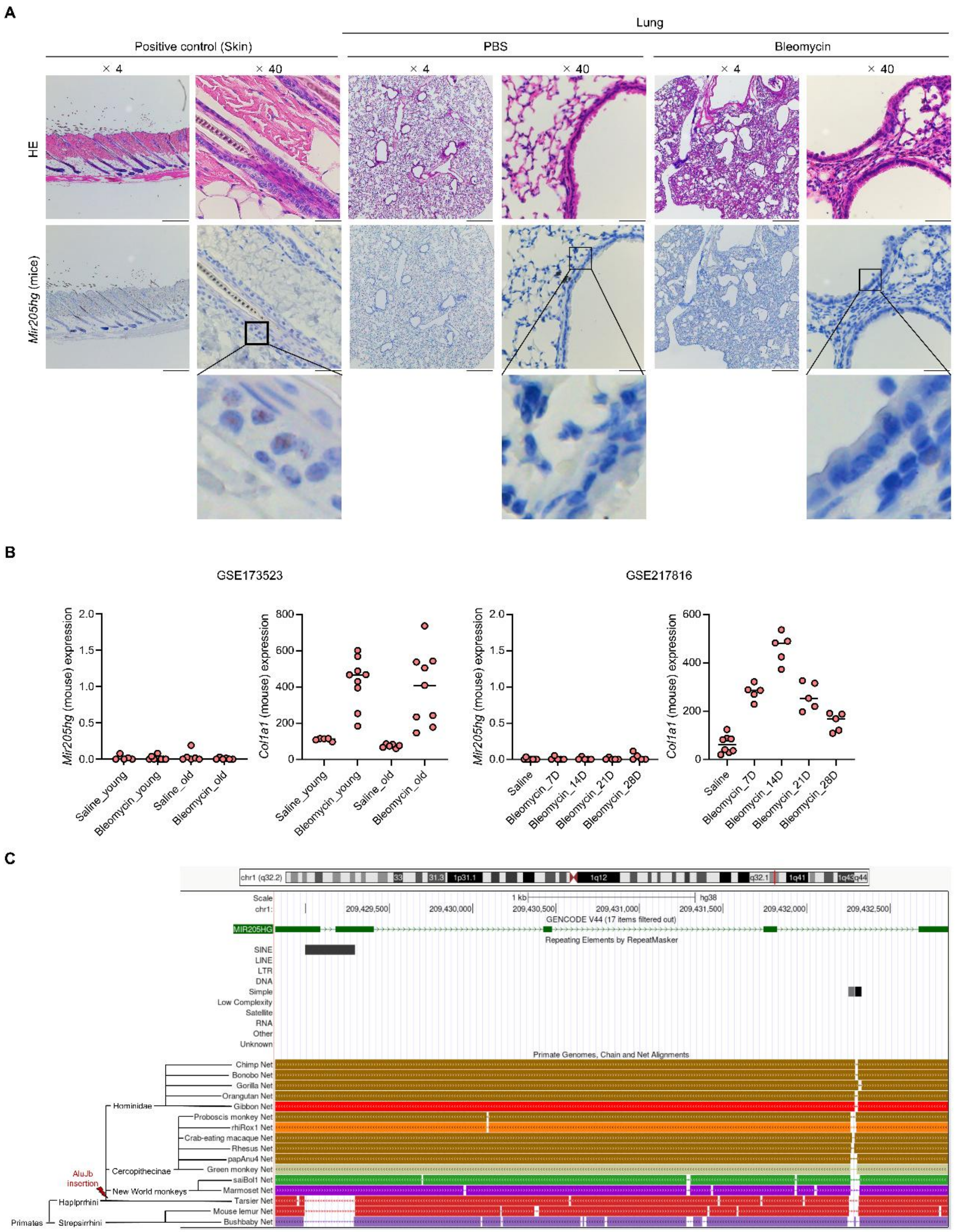

Supplemental Figure 16

Mir205hg in a bleomycin-induced pulmonary fibrosis mouse model

(A) Representative images of HE and *Mir205hg* ISH staining on day 21 after treatment with PBS and bleomycin (5 mg/kg, 60 µl). Mouse skin was used as a positive control.

Scale bars: 500 μm (left) and 50 μm (right).

(B) Expression of *Mir205hg and Col1a1* in saline (control) and bleomycin treated mouse. Public bulk RNA-seq datasets (GSE173523 (1) and GSE217816 (2)) were used.

(C) Primate phylogenetic tree model from the UCSC genome browser shown for *MIR205HG gene*. Hominidae, Cercopithecinae, and New World monkeys confirm the insertion of an *AluJb* element (SINE) into the *MIR205HG* gene.

Supplemental Table 1_Antibody

Supplementary Table 1_Antibody					
		Primary antibody			
**For IHC, IF**					
**Antibody**	**Clone**	**Cat. #**	**Animal Species**	**Dilution**	**Source**
Cytokeratin 5	XM26	ab17130	Mouse	1:200	Abcam
Prosurfactant Protein C	(Polyclonal)	AB3786	Rabbit	1:1000 (IHC) and 1:200 (IF)	Merck-Millipore
EpCAM	Ber-EP4	MA5-12604	Mouse	1:100	Thermo Fisher Scientific
IL-33	(Polyclonal)	HPA024426	Rabbit	1:200	Atlas Antibodies
GATA3	L50-823	390M	Mouse	1:800	Cell Marque
CD127	EPR23747-333	ab259806	Rabbit	1:200	Abcam
TP63	DO-7	NCL-L-p53-DO7	Mouse	1:500	Leoca Biotech
HTII-280	—	TB-27AHT2-280	Mouse	1:50	Terrace Biotech
NGFR	MRQ-21	304M	Mouse	1:200	Cell Marque
MUC5AC	CLH2	sc-33667	Mouse	1:400	Santa Cruz Biotechnology
Collagen1	EPR7785	ab138492	Rabbit	1:1500	Abcam
TTF-1	SP141	ab227652	Rabbit	1:100	Abcam
FUS	(Polyclonal)	11570-1-AP	Rabbit	1:200	Proteintech
Cytokeratin 17	E-4	sc-393002	Mouse	1:400	Santa Cruz Biotechnology
**For WB, IP**					
**Antibody**	**Clone**	**Cat. #**	**Animal Species**	**Dilution**	**Source**
IL-33	(Polyclonal)	HPA024426	Rabbit	1:200	Atlas Antibodies
FUS	(Polyclonal)	11570-1-AP	Rabbit	1:1000	Proteintech
β-actin	13E5	5125	Rabbit	1:1000	Cell Signaling Technology
**For FACS**					
**Antibody**	**Clone**	**Cat. #**	**Animal Species**	**Dilution**	**Source**
Alexa Fluor 647-conjugated anti-human EpCAM	9C4	324212	Mouse	1:50	BioLegend
HTII-280	—	TB-27AHT2-280	Mouse	1:50	Terrace Biotech
PE-conjugated anti-human NGFR	ME20.4	345106	Mouse	1:50	BioLegend
Anti-mouse IgG2b, k isotype control	—	400301	Mouse	1:500	BioLegend
		Secondary antibody			
**For IHC, IF**					
**Antibody**	**Clone**	**Cat. #**	**Animal Species**	**Dilution**	**Source**
Anti-Mouse EnVision+System-HRP Labelled Polymer	—	K40001	Goat	Ready to use (4-5 drops)	Agilent Technologies
Anti-Rabbit EnVision+System-HRP Labelled Polymer	—	AB3786	Goat	Ready to use (4-5 drops)	Agilent Technologies
Anti-Rabbit IgG (H+L) , Alexa Fluor 647	—	A27040	Goat	1:200	Invitrogen
Alexa Fluour 488-conjugated anti-mouse IgM	—	715-545-020	Mouse	1:100	Jackson ImmunoResearch
**For WB, IP**					
**Antibody**	**Clone**	**Cat. #**	**Animal Species**	**Dilution**	**Source**
Anti-Rabbit IgG (H+L) , pAb-HRP	—	458	Goat	1:1000	MBL
Anti-Mouse IgG (H+L) , pAb-HRP	—	330	Goat	1:1000	MBL
**For FACS**					
**Antibody**	**Clone**	**Cat. #**	**Animal Species**	**Dilution**	**Source**
Alexa Fluour 488-conjugated anti-mouse IgM	—	715-545-020	Mouse	1:100	Jackson ImmunoResearch
Alexa Fluour 647-conjugated anti-mouse IgG	—	A-31571	Mouse	1:50	Thermo Fisher Scientific
PE-conjugated anti-mouse IgG	—	400112	Mouse	1:50	BioLegend

Supplemental Table 2_ TaqMan® probe list

Ready-made products				
**Target gene**	**Taqman® probe ID**		**Cat. #**	**Source**
MIR205HG	Hs03405498_m1		4331182	Thermo Fisher Scientific
IL33 mRNA	Hs04931857_m1			
FUS	Hs01100224_m1			
SFTPC	Hs00161628_m1			
KRT5	Hs00361185_m1			
KRT17	Hs00356958_m1			
CDKN1A	Hs00355782_m1			
ACTB	Hs99999903_m1		4427975	
miR-205-3p	241040_mat			
miR-205-5p	000509			
RNU6B	001093			
Customised product				
**Target gene**	**Primer_sequence**	**Probe_sequence**	**Cat. #**	**Source**
IL33 pre-mRNA	(Forward) 5′-TGAACTTCCCATTCACATATGGAT-3′<br>(Reverse) 5′-AGGATCAGTCTTGCATTCAAATGA-3′	5'-FAM-TCCTCAGCTCCATAAGT-MGB-3'	4324034	Thermo Fisher Scientific

Supplemental Table 3_ Clinical data of all organoids

Alveolar organoids				
**Patients_#**	**Age**	**Gender**	**Background disease**	**Smoking status (Brinkman Index)**
#1	71	M	Lung Cancer (Squamous cell carcinoma)	750
#2	67	M	Lung Cancer (Adenocarcinoma)	920
#3	77	F	Lung Cancer (Adenocarcinoma)	0
**IPF-patient-derived airway organoids**				
**Patients_#**	**Age**	**Gender**	**Background disease**	**Smoking status (Brinkman Index)**
#1	71	M	Lung Cancer (Squamous cell carcinoma), Interstitial pneumonia	0
#2	60	M	Lung transplantation, , Interstitial pneumonia	400
#3	77	M	Lung Cancer (Adenocarcinoma), , Interstitial pneumonia	> 1000

Supplemental Table 4_ Human lung organoid medium

Reagent name	Supplier	Cat No.	Final concentration
Recombinant human EGF	Gibco	PHG0311	50 ng/mL
Recombinant human IGF-1	Biolegend	590908	100 ng/mL
Recombinant human FGF-basic	Gibco	13256029	20 ng/mL
Recombinant human FGF-10	Peprotech	100-26	100 ng/mL
Recombinant human KGF (FGF-7)	Peprotech	100-19	5 ng/mL
Afamin-Wnt-3a	Medical biological laboratories	J2-001	10% v/v
Recombinant human R-spondin-1	Peprotech	120-38	200 ng/mL
Recombinant human Noggin	Peprotech	120-10C	100 ng/mL
Recombinant human heregulinβ-1	Peprotech	100-03	100 ng/mL
A83-01	Tocris	2939	500 nM
Y-27632	Abmole	036-24023	10 μM
N-Acetyl-L-cysteine	Sigma-Aldrich	A9165	1.25 mM
B-27 Supplement	Gibco	17504044	50× diluted
Penicillin/Streptomycin	Wako/Nacalai tesque	022-18132/32204-92	100/100 U/mL
DMEM/Ham's F-12	Nacalai tesque	08460-95	-

Supplemental Table 5_ ChRIP probes for *MIR205HG*

	Plate Designator	Sequence	Sequence Name	Production Number	Scale	Three Modification
1	*MIR205HG*	agtcaagggtgagcaagagg	*MIR205HG* _1	SS781450-01	50 nmol	Biotin
2	*MIR205HG*	gagagagtttggagtatggt	*MIR205HG* _2	SS781450-02	50 nmol	Biotin
3	*MIR205HG*	gcggaacagaaatgactcct	*MIR205HG* _3	SS781450-03	50 nmol	Biotin
4	*MIR205HG*	tgcagaacagagagatggct	*MIR205HG* _4	SS781450-04	50 nmol	Biotin
5	*MIR205HG*	tccacagatttcagactcac	*MIR205HG* _5	SS781450-05	50 nmol	Biotin
6	*MIR205HG*	gacttgcagtgaatatggca	*MIR205HG* _6	SS781450-06	50 nmol	Biotin
7	*MIR205HG*	ccctggaaatccttgagaaa	*MIR205HG* _7	SS781450-07	50 nmol	Biotin
8	*MIR205HG*	agctacaatcttggtgctta	*MIR205HG* _8	SS781450-08	50 nmol	Biotin
9	*MIR205HG*	cacgtcctaaattttccagt	*MIR205HG* _9	SS781450-09	50 nmol	Biotin
10	*MIR205HG*	atccagtgaccactttcttc	*MIR205HG* _10	SS781450-10	50 nmol	Biotin
11	*MIR205HG*	ccatggttacatgggtctag	*MIR205HG* _11	SS781450-11	50 nmol	Biotin
12	*MIR205HG*	cctgtatcaggagacttgac	*MIR205HG* _12	SS781450-12	50 nmol	Biotin
13	*MIR205HG*	gtgagttctttccttatcac	*MIR205HG* _13	SS781450-13	50 nmol	Biotin
14	*MIR205HG*	tccaagatgggtacttgaga	*MIR205HG* _14	SS781450-14	50 nmol	Biotin
15	*MIR205HG*	acatgtacagtccactagat	*MIR205HG* _15	SS781450-15	50 nmol	Biotin
16	*MIR205HG*	attctttgccttctctttag	*MIR205HG* _16	SS781450-16	50 nmol	Biotin
17	*MIR205HG*	gaagaaagaggccagtctgt	*MIR205HG* _17	SS781450-17	50 nmol	Biotin
18	*MIR205HG*	tgagaggaaaatgcaccctg	*MIR205HG* _18	SS781450-18	50 nmol	Biotin
19	*MIR205HG*	cagagatctgggtagtgagg	*MIR205HG* _19	SS781450-19	50 nmol	Biotin
20	*MIR205HG*	tctagctagtgctgagaagg	*MIR205HG* _20	SS781450-20	50 nmol	Biotin
21	*MIR205HG*	gaggcagtttctcgtatgtg	*MIR205HG* _21	SS781450-21	50 nmol	Biotin
22	*MIR205HG*	cagtgcaagccccataaaag	*MIR205HG* _22	SS781450-22	50 nmol	Biotin
23	*MIR205HG*	aacctggctttcatagactg	*MIR205HG* _23	SS781450-23	50 nmol	Biotin
24	*MIR205HG*	cttatctaggaaggacagcc	*MIR205HG* _24	SS781450-24	50 nmol	Biotin
25	*MIR205HG*	aaaaaccacaactcctggct	*MIR205HG* _25	SS781450-25	50 nmol	Biotin
26	*MIR205HG*	cacaaggcactgtccaaaga	*MIR205HG* _26	SS781450-26	50 nmol	Biotin
27	*MIR205HG*	ggtgttctgcaattgcttta	*MIR205HG* _27	SS781450-27	50 nmol	Biotin
28	*MIR205HG*	ataagtccatgtggaacctc	*MIR205HG* _28	SS781450-28	50 nmol	Biotin
29	*MIR205HG*	cacagacagacagtgctgag	*MIR205HG* _29	SS781450-29	50 nmol	Biotin
30	*MIR205HG*	caaggacctgaatactccta	*MIR205HG* _30	SS781450-30	50 nmol	Biotin
31	*MIR205HG*	gggatgcatgtcaacctcag	*MIR205HG* _31	SS781450-31	50 nmol	Biotin
32	*MIR205HG*	atctggttgggtatgagaca	*MIR205HG* _32	SS781450-32	50 nmol	Biotin
33	*MIR205HG*	ctccagatgtctccttcatt	*MIR205HG* _33	SS781450-33	50 nmol	Biotin
34	*MIR205HG*	acgcaaattttcatagcatc	*MIR205HG* _34	SS781450-34	50 nmol	Biotin

Supplemental Table 6_ RNA sequences used in SPR and NMR experiments

**Name**	**Sequence (5' to 3')**
MIR205HG _IL001	GUACCCCGCAUGUUUCAUGUCAGGUAC
MIR205HG _IL002	GUACGUCAUGUUUCAUGAGCAUGUUUCAUGCGAUUCGUAC
MIR205HG _IL003	GUACGGCAUGUUUCAUGCUCGUAC
MIR205HG _IL004	GUACCCUCACAUGUUUCAUGUCAAGGUAC
MIR205HG _HL001	GUACCAUGUCUCACAUGGUAC
MIR205HG _HL002	GUACCAUGAAUCUUUCAUGGUAC
MIR205HG _HL003	GUACCAUGUCCCACAUGGUAC

References

1. Klee S, Picart-Armada S, Wenger K, Birk G, Quast K, Veyel D, et al. Transcriptomic and proteomic profiling of young and old mice in the bleomycin model reveals high similarity. *Am J Physiol Lung Cell Mol Physiol.* 2023;324(3):L245-L58.
2. Arif M, Basu A, Wolf KM, Park JK, Pommerolle L, Behee M, et al. An Integrative Multiomics Framework for Identification of Therapeutic Targets in Pulmonary Fibrosis. *Adv Sci (Weinh).* 2023;10(16):e2207454.
3. Adams TS, Schupp JC, Poli S, Ayaub EA, Neumark N, Ahangari F, et al. Single-cell RNA-seq reveals ectopic and aberrant lung-resident cell populations in idiopathic pulmonary fibrosis. *Science Advances.* 2020;6(28):eaba1983.
4. Zhang Z, Schwartz S, Wagner L, and Miller W. A Greedy Algorithm for Aligning DNA Sequences. *Journal of Computational Biology.* 2000;7(1-2):203-14.
5. Chen Y, and Wang X. miRDB: an online database for prediction of functional microRNA targets. *Nucleic Acids Res.* 2020;48(D1):D127-D31.
6. Bandyopadhyay S, and Mitra R. TargetMiner: microRNA target prediction with systematic identification of tissue-specific negative examples. *Bioinformatics.* 2009;25(20):2625-31.
7. Wang Y, Song F, Zhang B, Zhang L, Xu J, Kuang D, et al. The 3D Genome Browser: a web-based browser for visualizing 3D genome organization and long-range chromatin interactions. *Genome Biol.* 2018;19(1):151.
8. Andrews RJ, Rouse WB, O'Leary CA, Booher NJ, and Moss WN. ScanFold 2.0: a rapid approach for identifying potential structured RNA targets in genomes and transcriptomes. *PeerJ.* 2022;10:e14361.
9. Gruber AR, Lorenz R, Bernhart SH, Neubock R, and Hofacker IL. The Vienna RNA websuite. *Nucleic Acids Res.* 2008;36(Web Server issue):W70-4.
10. Kerpedjiev P, Hammer S, and Hofacker IL. Forna (force-directed RNA): Simple and effective online RNA secondary structure diagrams. *Bioinformatics.*

2015;31(20):3377-9.

11. Plateau P. Exchangeable proton NMR without base-line distorsion, using new strong-pulse sequences. *J Am Chem Soc* 1982;104:7310–1.

12. DePianto DJ, Heiden JAV, Morshead KB, Sun KH, Modrusan Z, Teng G, et al. Molecular mapping of interstitial lung disease reveals a phenotypically distinct senescent basal epithelial cell population. *JCI Insight.* 2021;6(8).
