# Supplementary material for: Involvement of lncRNA MIR205HG in idiopathic pulmonary fibrosis and IL-33 regulation via Alu elements
Source: JCI Insight. 2025 Mar 10;10(5):e187172. doi: 10.1172/jci.insight.187172 (PMC11949018; doi:10.1172/jci.insight.187172)

Figure 7G

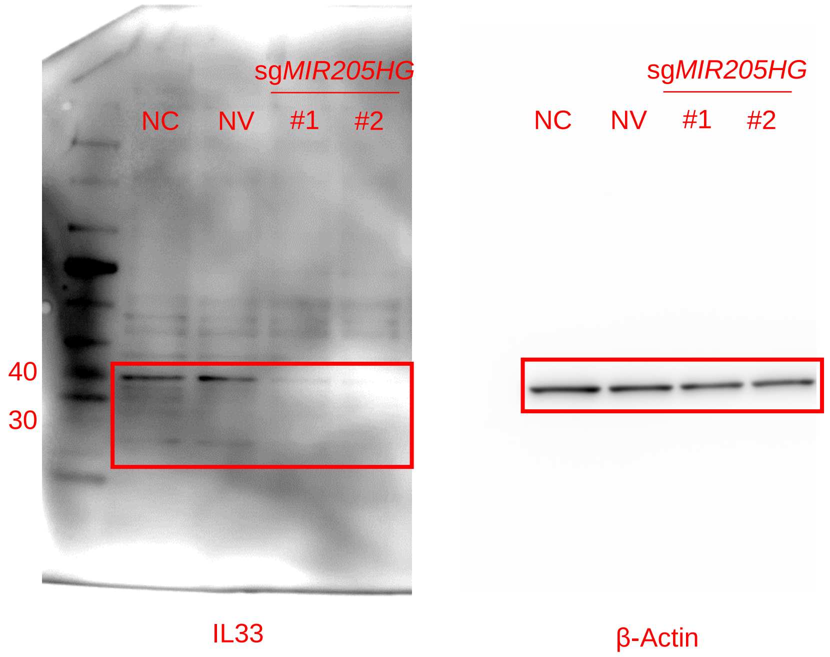

Figure 8D

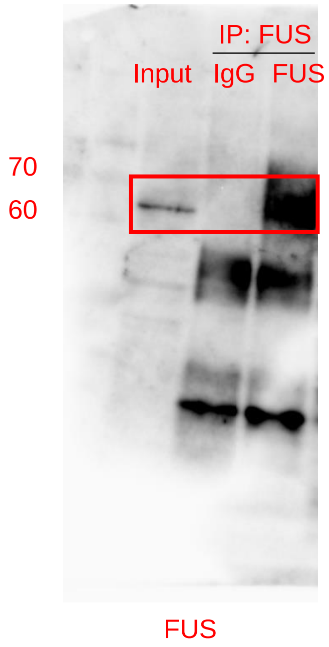

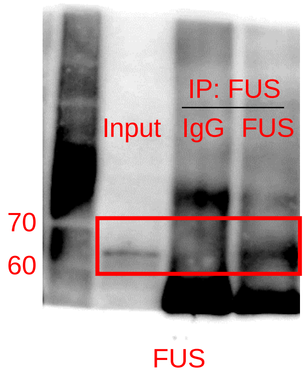

Figure 10E

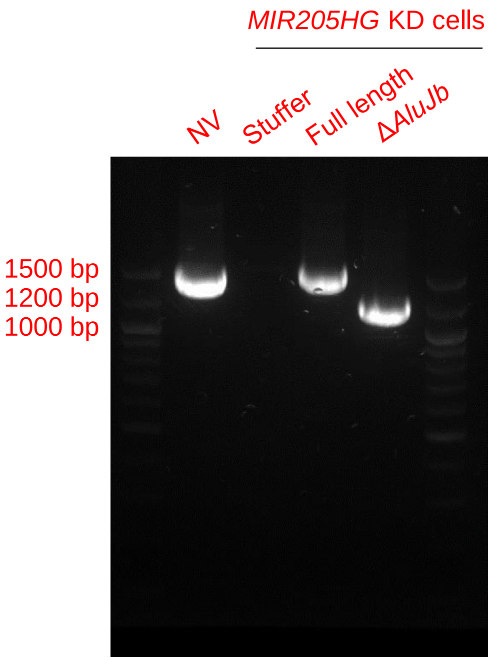

MIR205HG KD cells
NV
Stuffer
Full length
ΔAluJb
1500 bp
1200 bp
1000 bp

Figure 12C

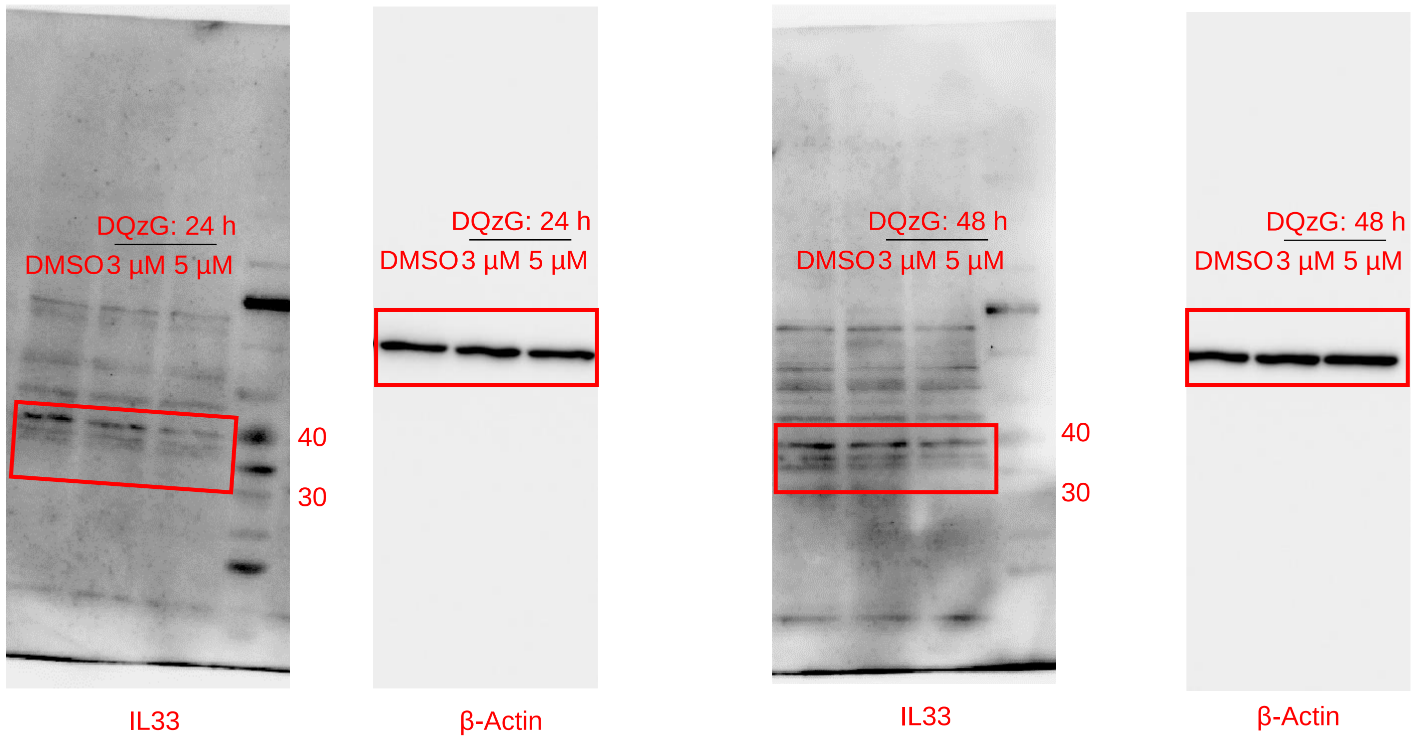
DQzG: 24 h
DMSO 3 µM 5 µM
40
30
IL33
DQzG: 24 h
DMSO 3 µM 5 µM
β-Actin
DQzG: 48 h
DMSO 3 µM 5 µM
40
30
IL33
DQzG: 48 h
DMSO 3 µM 5 µM
β-Actin

Figure 12D

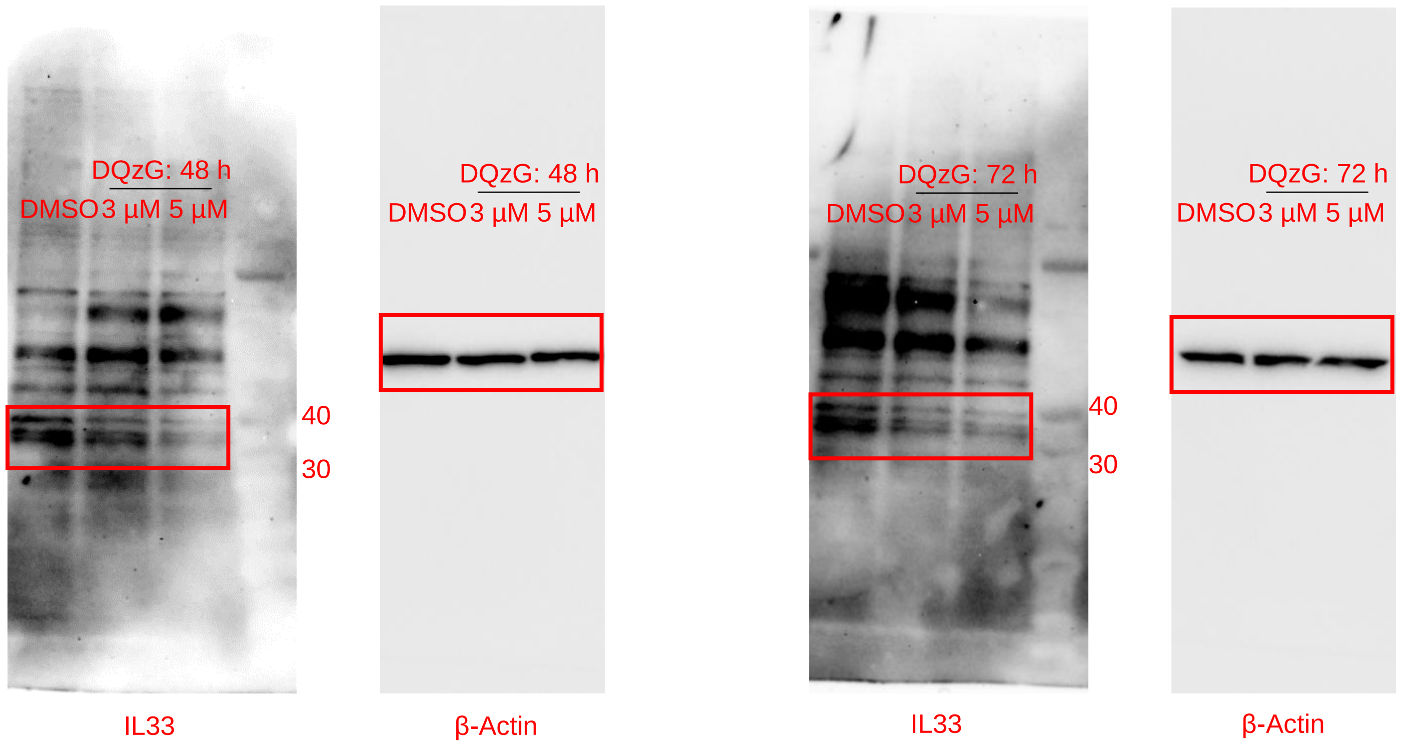
DQzG: 48 h
DMSO 3 μM 5 μM
40
30
IL33
DQzG: 48 h
DMSO 3 μM 5 μM
β-Actin
DQzG: 72 h
DMSO 3 μM 5 μM
40
30
IL33
DQzG: 72 h
DMSO 3 μM 5 μM
β-Actin

Supplemental Figure 9A

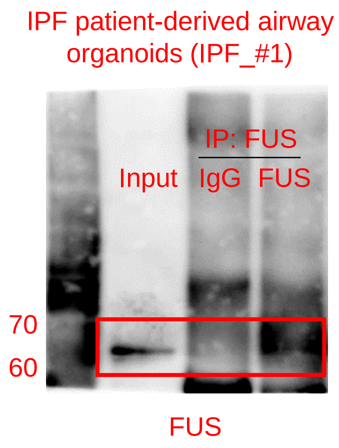

Supplemental Figure 9E

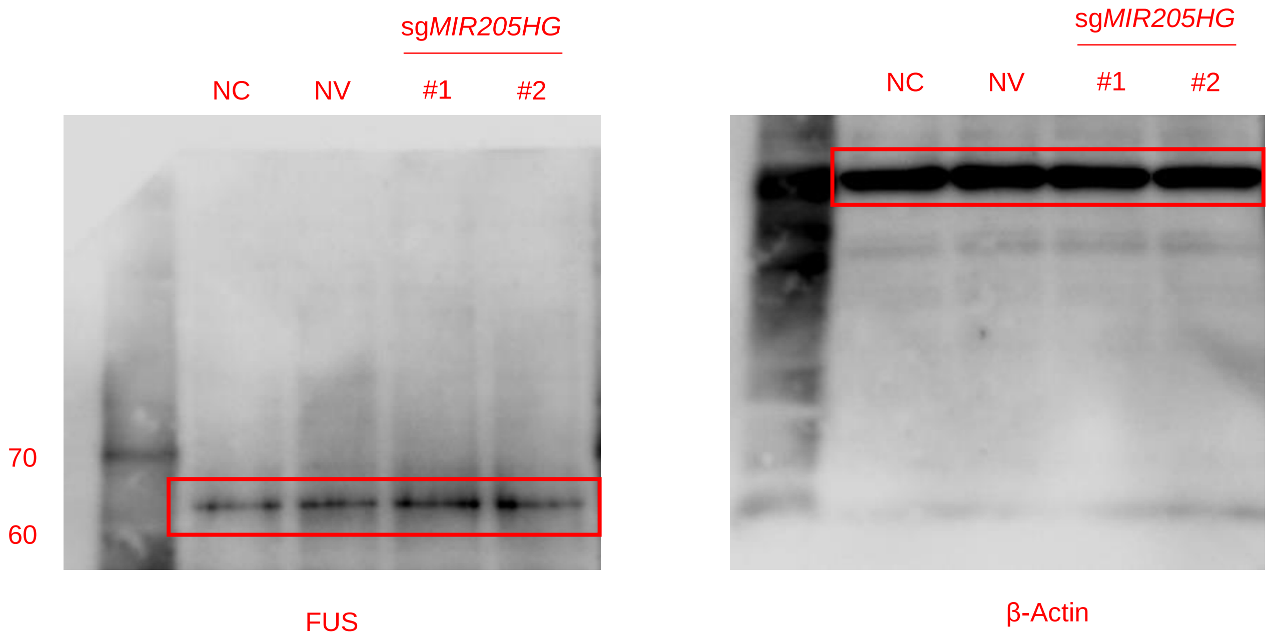

Supplemental Figure 9G

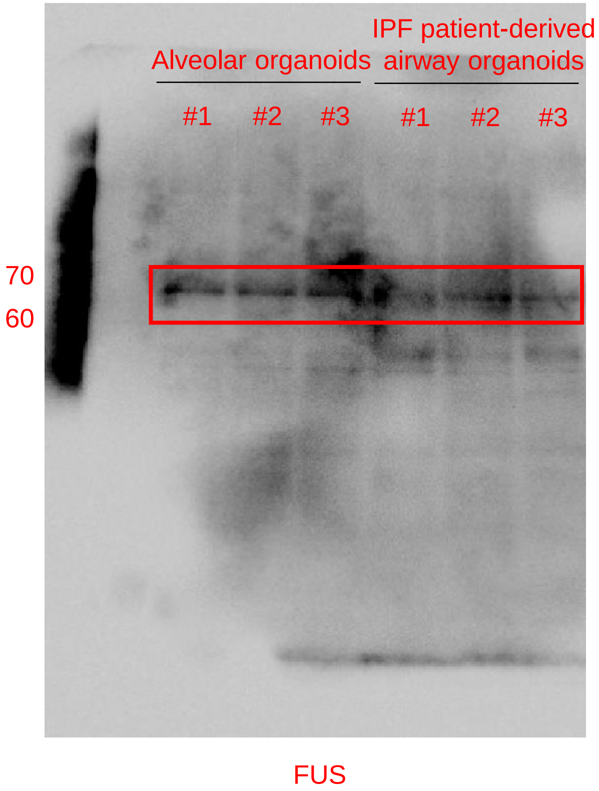

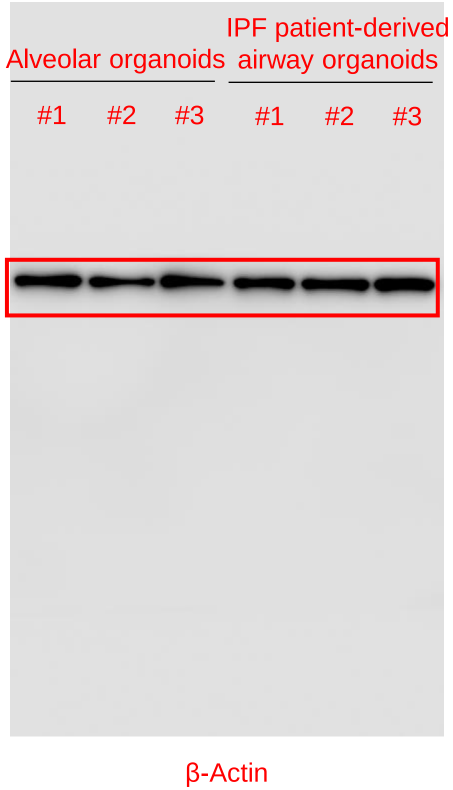

Supplement: Unedited blot and gel images [file jciinsight-10-187172-s027.pdf]
